# Supplementary material for: Mesenchymal tumor organoid models recapitulate rhabdomyosarcoma subtypes
Source: EMBO Mol Med. 2022 Aug 2;14(10):e16001. doi: 10.15252/emmm.202216001 (PMC9549731; doi:10.15252/emmm.202216001)
Supplement: Supplementary file 1 — Appendix [file EMMM-14-e16001-s004.pdf]

Appendix

Table of contents

- Appendix Figure S1: Correlation analysis of Area Under the Curve (AUC) values from technical replicates of drug screenings

Appendix Figure S1

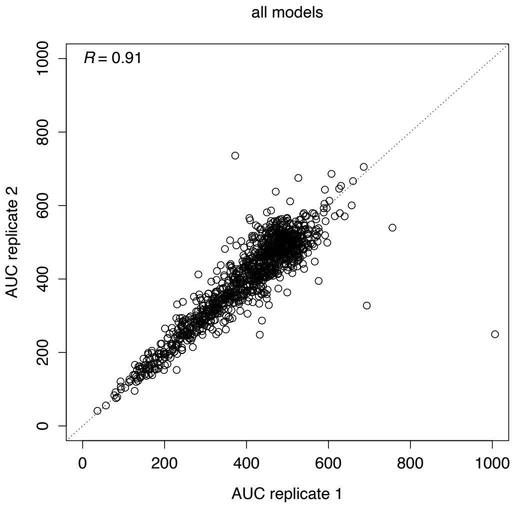

Appendix Figure S1 legend: Correlation analysis of Area Under the Curve (AUC) values (n = 165) from technical replicates of drug screenings of RMS007, RMS109, RMS110, RMS000EEC, RMS000FLV, RMS000HQC, and RMS000HWQ. Overall, the correlation coefficient (*R*) is 0.91 (Pearson).
