# Supplementary material for: Mesenchymal tumor organoid models recapitulate rhabdomyosarcoma subtypes
Source: EMBO Mol Med. 2022 Aug 2;14(10):e16001. doi: 10.15252/emmm.202216001 (PMC9549731; doi:10.15252/emmm.202216001)
Supplement: Supplementary file 2 — Expanded View Figures PDF [file EMMM-14-e16001-s010.pdf]

Expanded View Figures

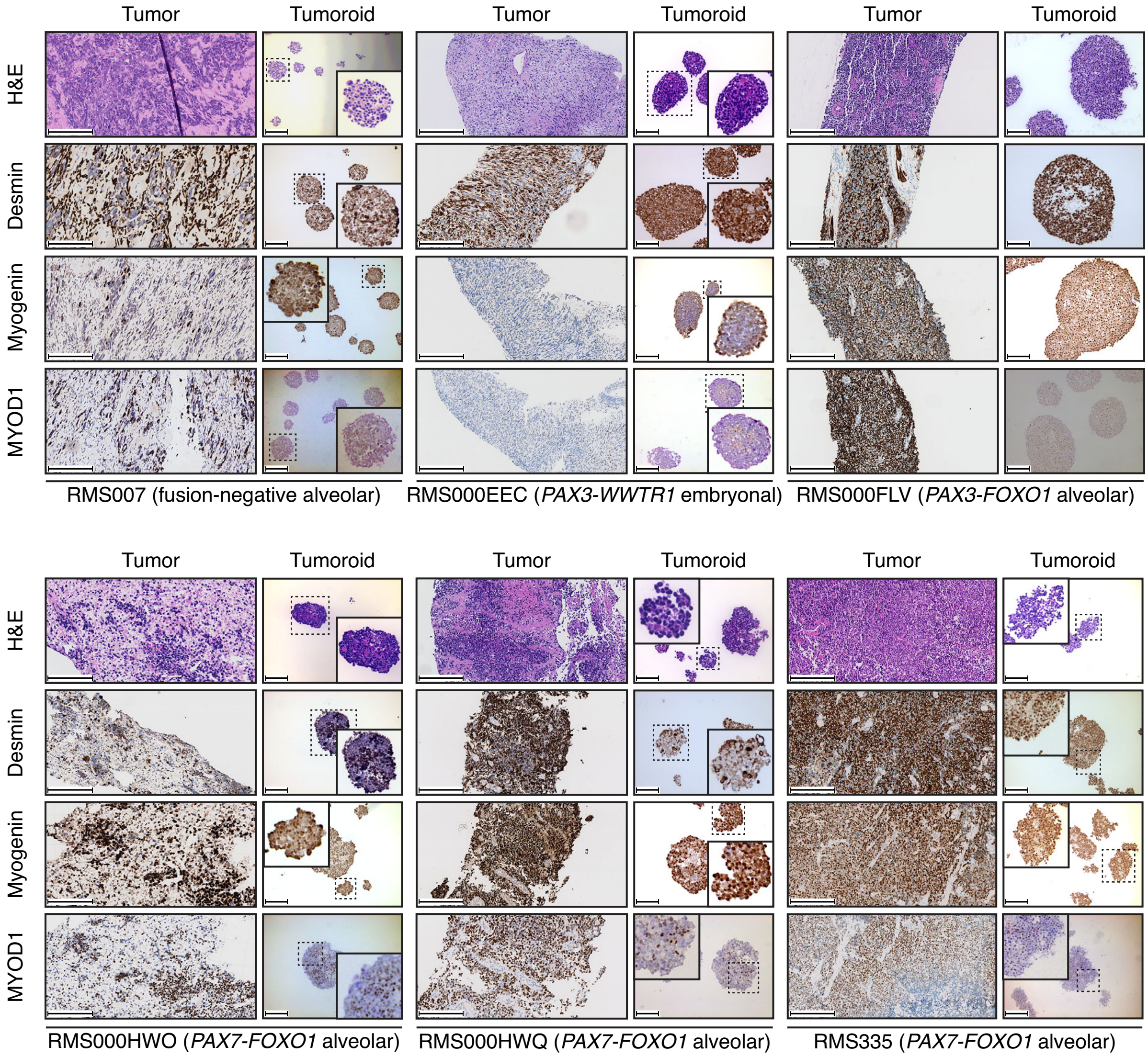

Figure EV1. Retained marker protein expression (additional figures).

Morphological (via H&E) and immunohistochemical (IHC) comparison of RMS tumors and derived RMS tumoroid models shows retained marker protein (Desmin, Myogenin, and MYOD1) expression and cellular morphology. Scale bars equal 200 µm (RMS007, RMS000HWO, RMS335) or 100 µm (RMS000EEC, RMS000FLV, RMS000HWQ). For RMS tumoroid models that form small spheres a zoom-in image of a sphere was inserted.

 © 2022 The Authors

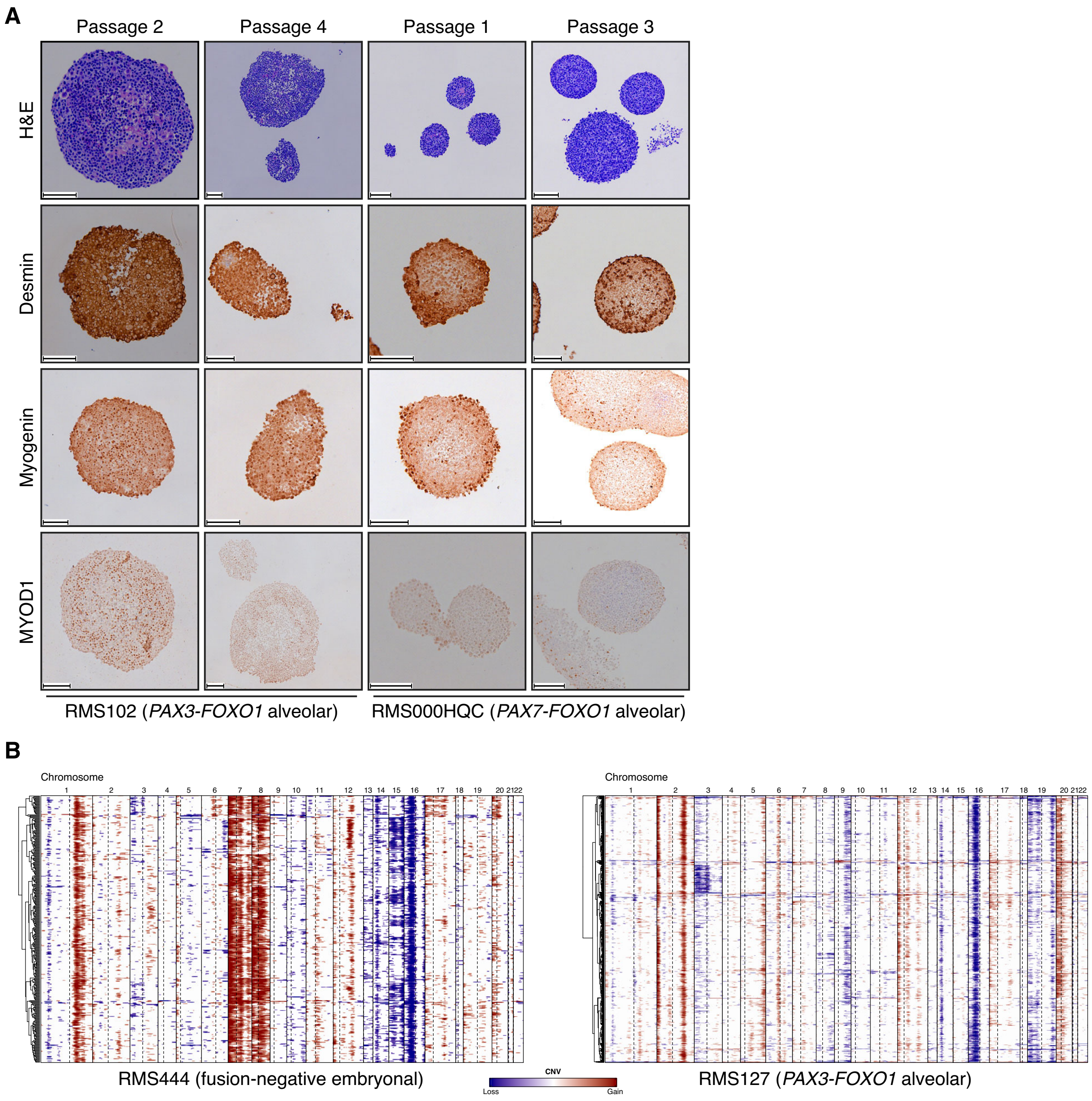

Figure EV2. Marker protein expression during early passages of culturing and single-cell RNA-seq inferred per cell CNV profiles.

A H&E and immunohistochemistry (IHC) stainings of early passage RMS102 (passage 2 and passage 4 – passage 6 used for drug screening) and RMS000HQC (passage 1 and passage 3 – passage 5 used for drug screening). Scale bars equal 100 μm.

B Heatmaps showing the inferred CNV profiles of single cells (*y*-axis) from the RMS127 and RMS444 tumoroid models, respectively. Chromosome arms are delineated by dotted lines where applicable.

Figure EV3. RMS tumoroid models molecularly resemble the tumor they are derived from (additional figures).

A Examples of circular copy number plots of fusion-negative RMS with embryonal (RMS000ETY) or alveolar (RMS007) histology. Outer circle depicts the tumoroid model, inner circle depicts the tumor.

B Examples of circular copy number plots of fusion-positive RMS with alveolar histology and *PAX3-FOXO1* fusion (RMS410) or *PAX7-FOXO1* fusion (RMS000HQC). Outer circle depicts the tumoroid model, inner circle depicts the tumor.

C Contribution of averaged single base substitution (SBS) profiles for RMS tumors (upper row) and tumoroid models (lower row).

D Clustered correlogram of SBS profiles detected in RMS tumors (T) and tumoroid models (O).

E Comparison of total number of mutations (synonymous and nonsynonymous) in RMS tumors and tumoroid models per fusion-type (fusion-negative $n = 4$, fusion-positive = 15, Wilcoxon test, $P = 0.15$ for tumors, $P = 0.02$ for tumoroid models). Data are shown as boxplots: The central line in the box represents the median; the upper and lower limits of the box represent the third and first quartile, respectively; the whiskers represent the minimum (bottom) and maximum (top) values excluding outliers, which are plotted as individual points.

© 2022 The Authors

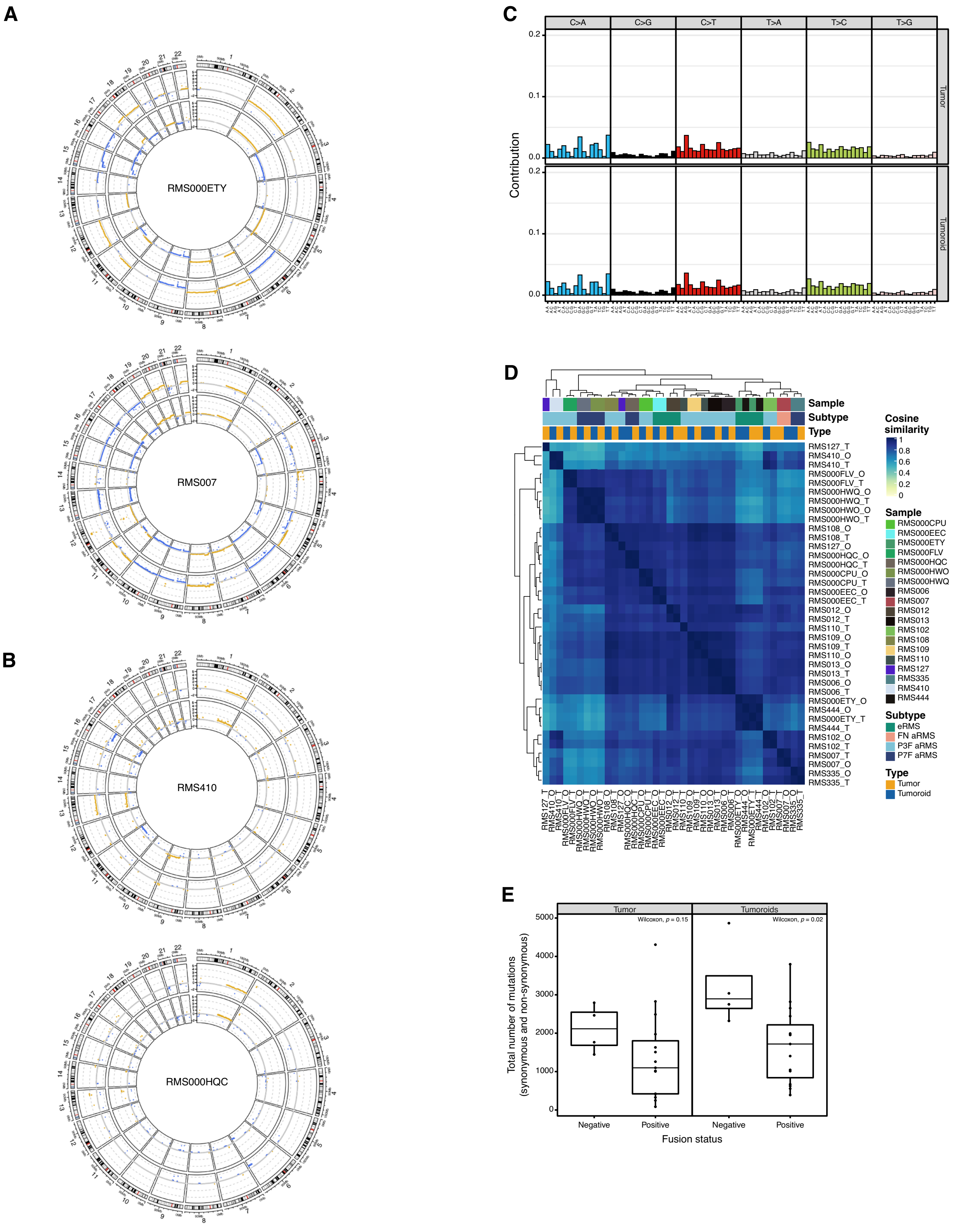
A
RMS000ETY
RMS007
B
RMS410
RMS000HQC
C
C>A
C>G
C>T
T>A
T>C
T>G
Contribution
Tumor
Tumoroid
D
Sample
Subtype
Type
RMS127_T
RMS410_O
RMS410_T
RMS000FLV_O
RMS000FLV_T
RMS000HWQ_O
RMS000HWQ_T
RMS000HWO_O
RMS000HWO_T
RMS108_O
RMS108_T
RMS127_O
RMS000HQC_O
RMS000HQC_T
RMS000CPU_O
RMS000CPU_T
RMS000EEC_O
RMS000EEC_T
RMS012_O
RMS012_T
RMS110_T
RMS109_O
RMS109_T
RMS110_O
RMS013_O
RMS013_T
RMS006_O
RMS006_T
RMS000ETY_O
RMS444_O
RMS000ETY_T
RMS444_T
RMS102_O
RMS102_T
RMS007_T
RMS007_O
RMS335_O
RMS335_T
Cosine similarity
1
0.8
0.6
0.4
0.2
0
Sample
RMS000CPU
RMS000EEC
RMS000ETY
RMS000FLV
RMS000HQC
RMS000HWO
RMS000HWQ
RMS006
RMS007
RMS012
RMS013
RMS102
RMS108
RMS109
RMS110
RMS127
RMS335
RMS410
RMS444
Subtype
eRMS
FN aRMS
P3F aRMS
P7F aRMS
Type
Tumor
Tumoroid
E
Tumor
Tumoroids
Wilcoxon, p = 0.15
Wilcoxon, p = 0.02
Total number of mutations (synonymous and non-synonymous)
5000
4000
3000
2000
1000
0
Negative
Positive
Negative
Positive
Fusion status

Figure EV3.

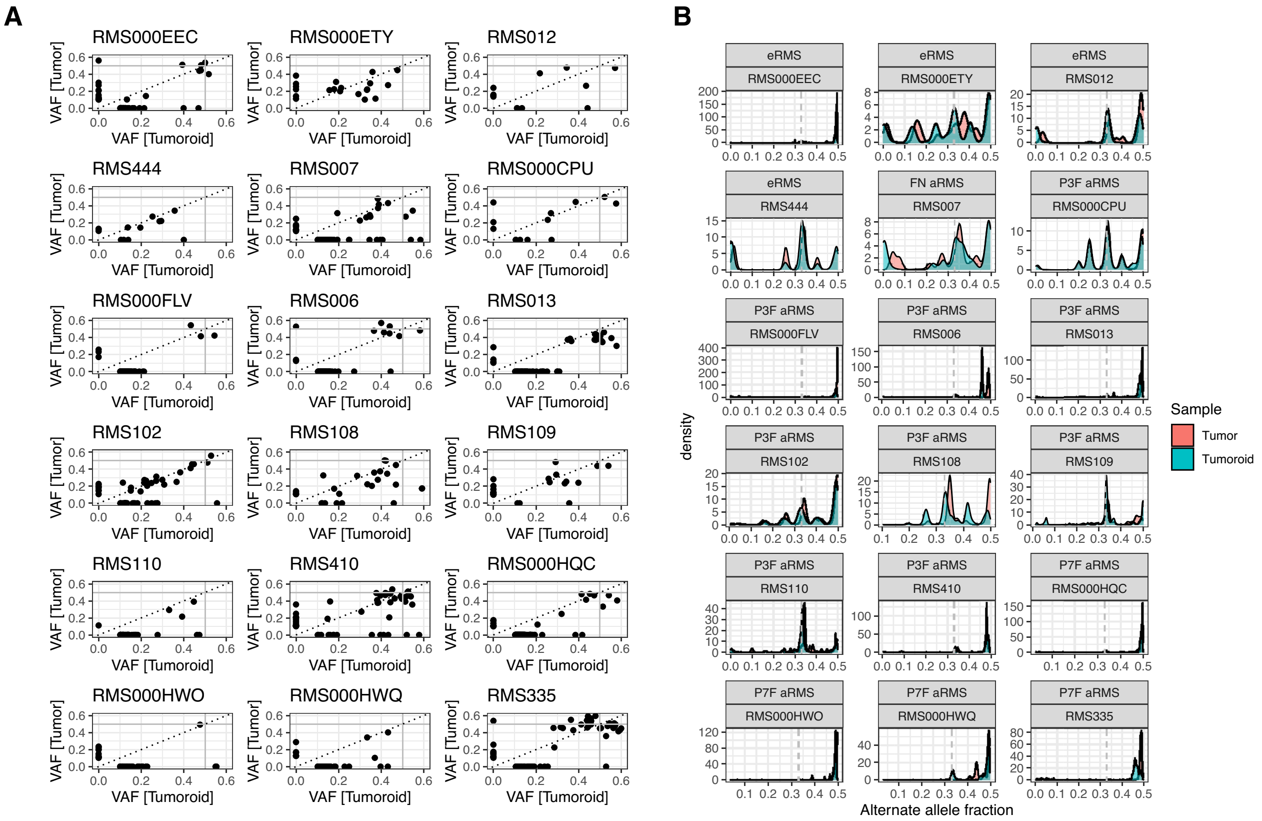

Figure EV4. Established RMS tumoroid models maintain the clonal composition of the RMS tumor they were derived from to a large extent.

A Correlogram of variant allele fraction (VAF) of single-nucleotide variants (SNVs) detected in RMS tumors and derived tumoroid models per pair.

B Density plots of alternate allele (B-allele) fraction over the entire genome per RMS tumor (red) and derived RMS tumoroid model (blue) per pair.

 © 2022 The Authors

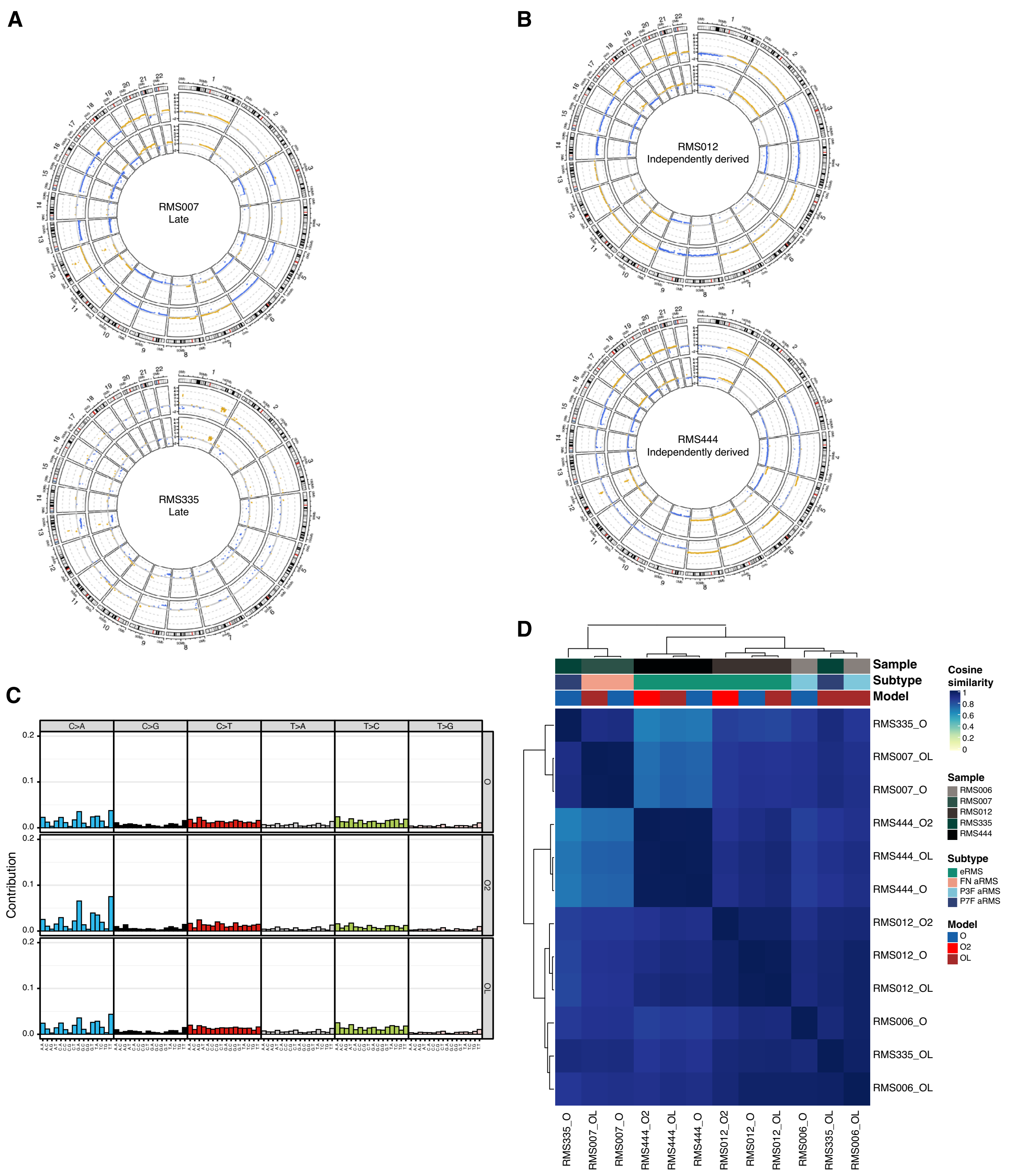

Figure EV5. Genetic and transcriptional stability of tumoroid models over time (additional figures).

A Circular copy number plots of standard and late passage RMS tumoroid models of a fusion-negative alveolar (RMS007) and a *PAX7-FOXO1* positive alveolar (RMS335) RMS. Outer circle depicts the standard passage tumoroid, inner circle depicts the late passage tumoroid model.

B Circular copy number plots of standard passage and independently derived RMS tumoroid models of two fusion-negative embryonal RMS (RMS012 and RMS444). Outer circle depicts the standard passage tumoroid, inner circle depicts the independently derived tumoroid model.

C Contribution of averaged single base substitution (SBS) profiles for standard (O), independently derived (O2), and late passage (OL) RMS tumoroid models.

D Clustered correlogram of SBS profiles detected in standard (O), independently derived (O2), and late passage (OL) RMS tumoroid models.

© 2022 The Authors

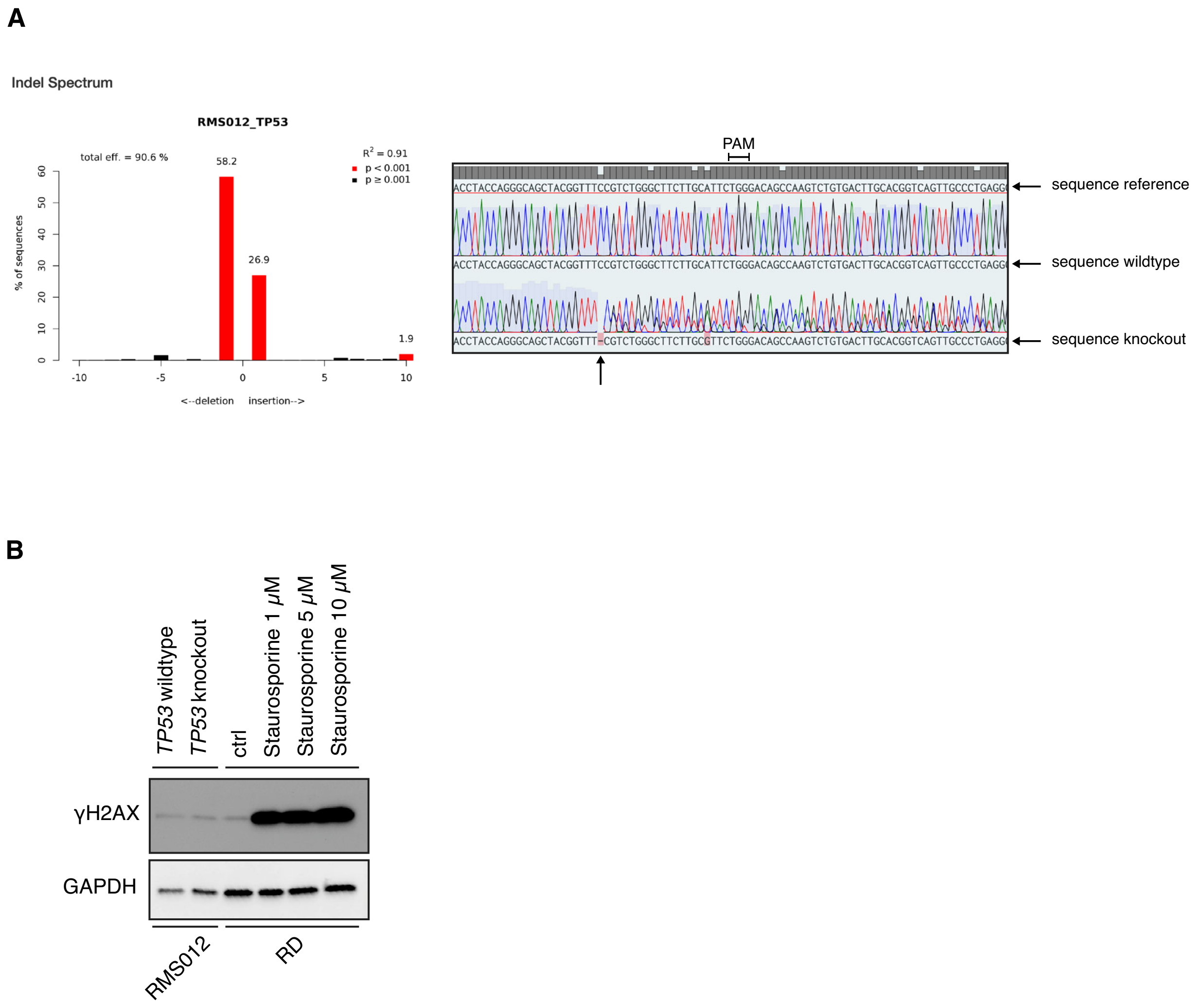

Figure EV6. RMS tumoroid models can be molecularly edited using CRISPR/Cas9 with P53-deficient eRMS being more sensitive to the checkpoint kinase inhibitor prexasertib (additional figures).

A Left panel: Inferred Indel Spectrum from Sanger sequencing using the TIDE tool (https://tide.nki.nl). Right panel: Sanger sequencing around Cas9 cut site (arrow at the bottom) in *TP53* gene. Protospacer Adjacent Motif (PAM) sequence indicated.

B Western Blotting analysis of γH2AX (Ser-139) as proxy for DNA double-strand breaks in untreated RMS012 tumoroid cells (*TP53* wildtype and *TP53* knockout). RMS cell line RD treated with staurosporine served as positive control while GAPDH served as loading control.

Source data are available online for this figure.

 © 2022 The Authors
