# Supplementary material for: Mesenchymal tumor organoid models recapitulate rhabdomyosarcoma subtypes
Source: EMBO Mol Med. 2022 Aug 2;14(10):e16001. doi: 10.15252/emmm.202216001 (PMC9549731; doi:10.15252/emmm.202216001)
Supplement: Supplementary file 5 — Source Data for Expanded View [file EMMM-14-e16001-s003.zip › figure_EV6/EV6B/EV6B_readme.rtf]

H2AX: replicate 1 (left lanes) and replicate 2 (right lanes)GAPHD: replicate 1 (left lanes) and replicate 2 (right lanes)
