# Supplementary figures and images for: Mesenchymal tumor organoid models recapitulate rhabdomyosarcoma subtypes
Source: EMBO Mol Med. 2022 Aug 2;14(10):e16001. doi: 10.15252/emmm.202216001 (PMC9549731; doi:10.15252/emmm.202216001)

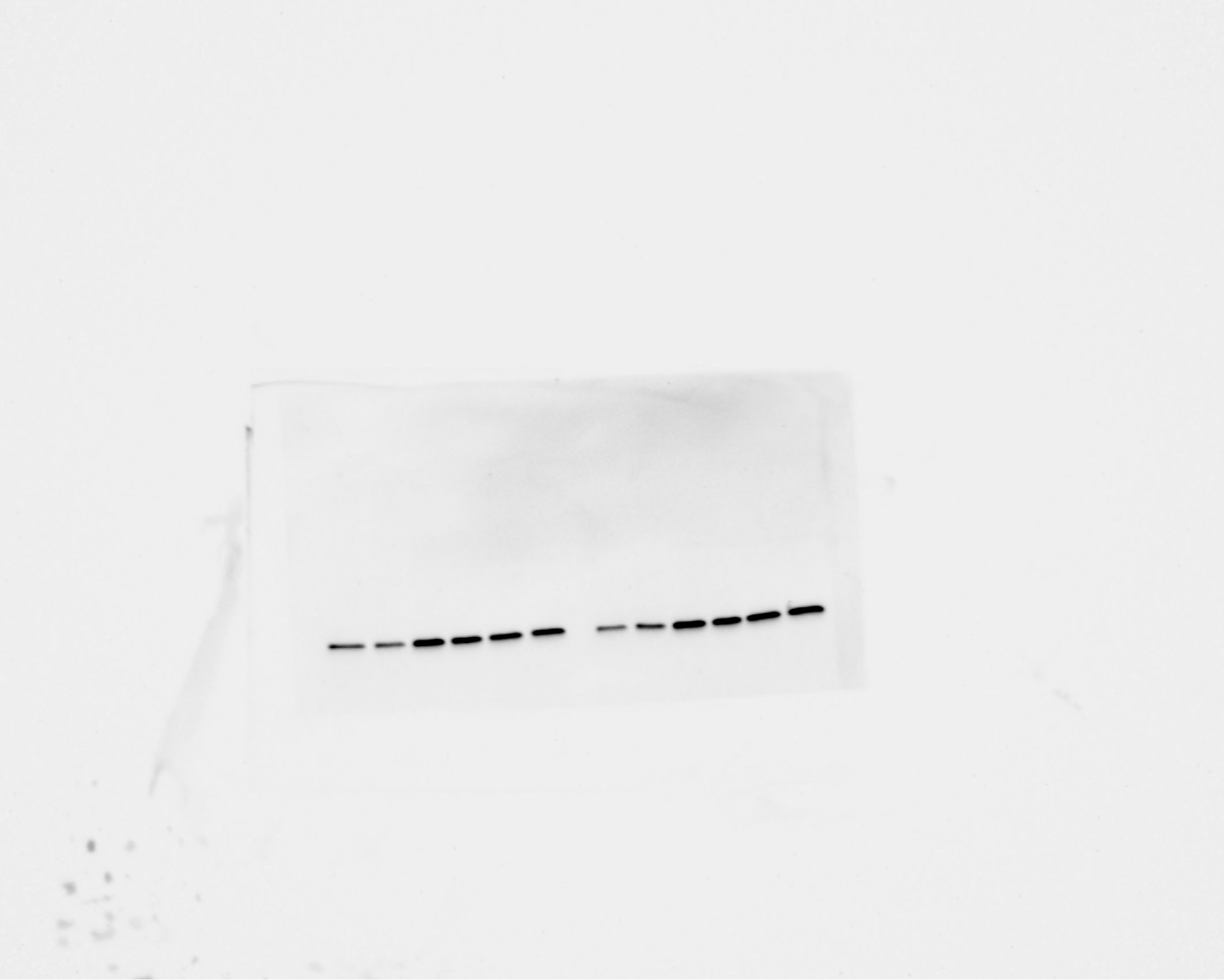

Supplement: Supplementary file 5 — Source Data for Expanded View [file EMMM-14-e16001-s003.zip › figure_EV6/EV6B/EV6B_GAPDH.tif]

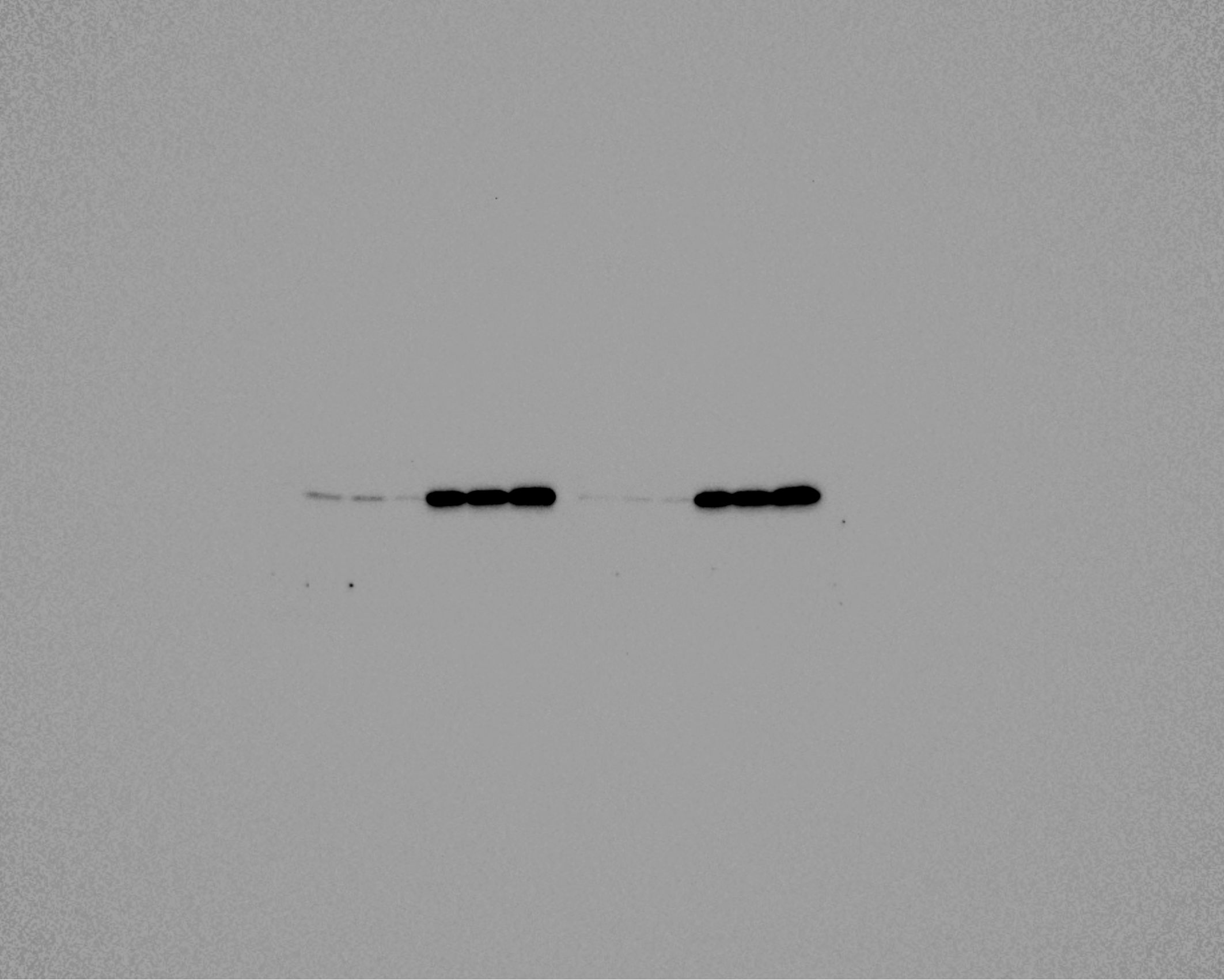

Supplement: Supplementary file 5 — Source Data for Expanded View [file EMMM-14-e16001-s003.zip › figure_EV6/EV6B/EV6B_H2AX.tif]

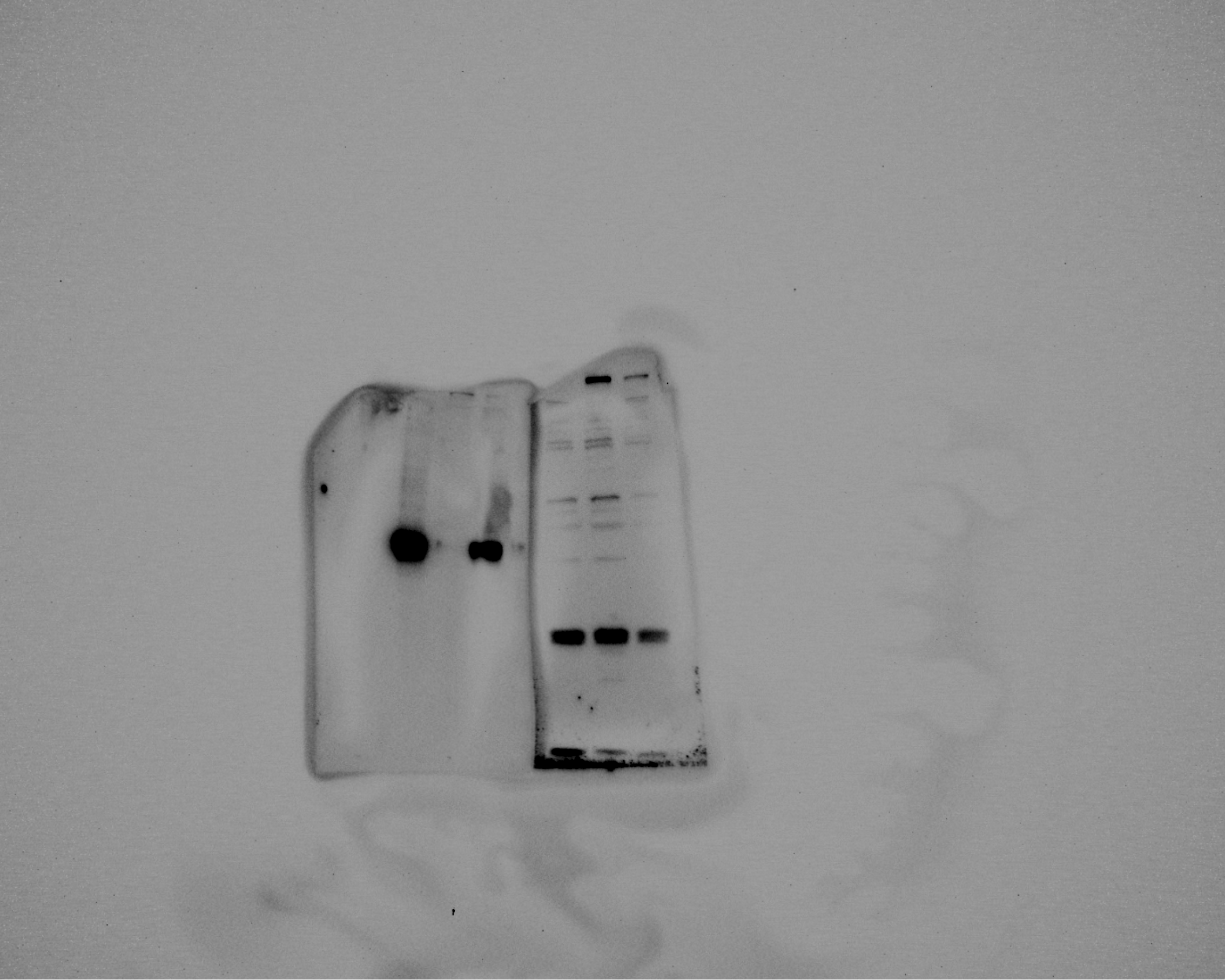

Supplement: Supplementary file 9 — Source Data for Figure 6 [file EMMM-14-e16001-s002.zip › figure_6/6B/6B_WB_rep2.tif]

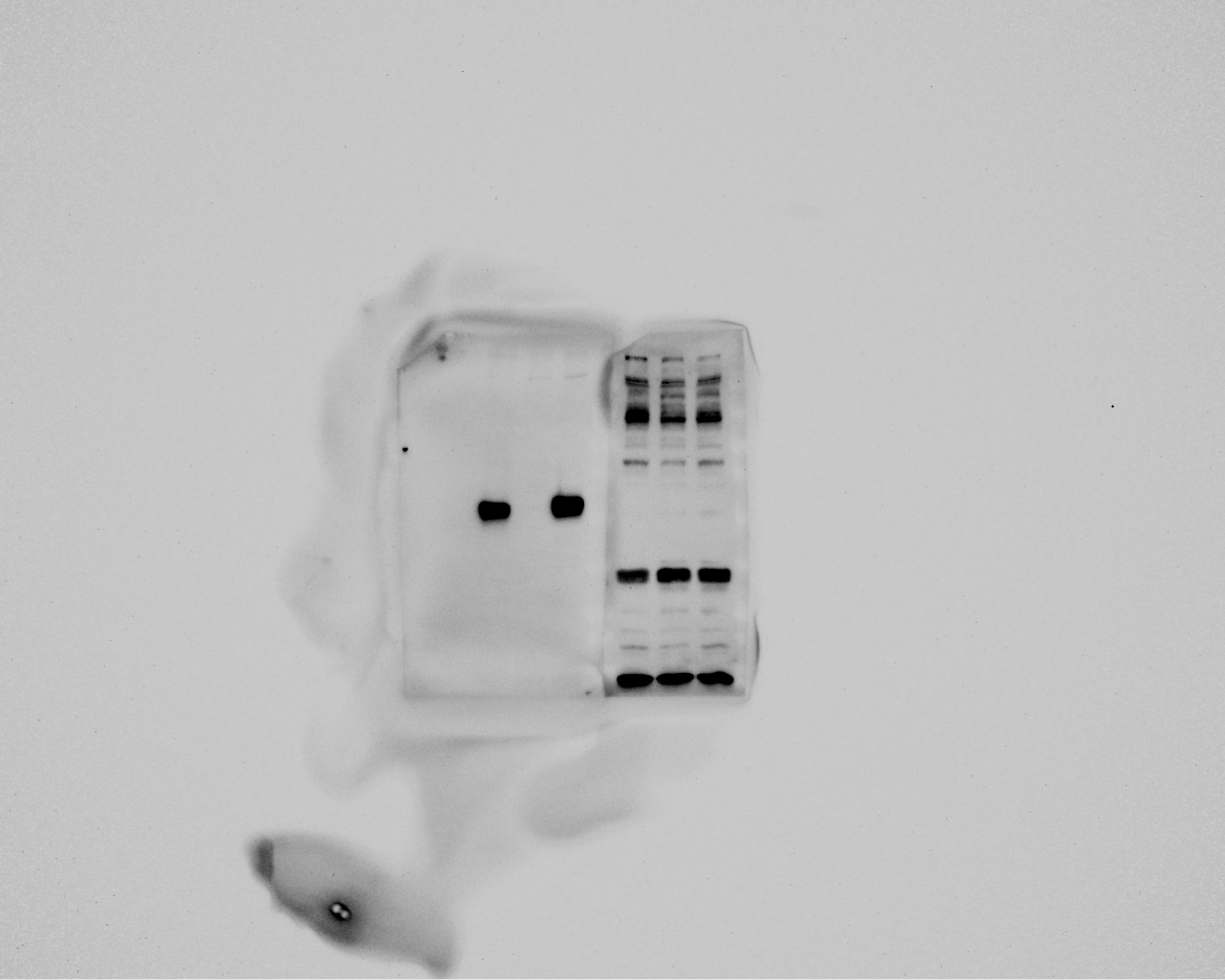

Supplement: Supplementary file 9 — Source Data for Figure 6 [file EMMM-14-e16001-s002.zip › figure_6/6B/6B_WB_rep1.tif]
