# Supplementary material for: Mesenchymal tumor organoid models recapitulate rhabdomyosarcoma subtypes
Source: EMBO Mol Med. 2022 Aug 2;14(10):e16001. doi: 10.15252/emmm.202216001 (PMC9549731; doi:10.15252/emmm.202216001)
Supplement: Supplementary file 6 — PDF+ [file EMMM-14-e16001-s005.pdf]

Article

SOURCE DATA TRANSPARENT PROCESS OPEN ACCESS

EMBO Molecular Medicine

Mesenchymal tumor organoid models recapitulate rhabdomyosarcoma subtypes

Michael T Meister[1,2], Marian J A Groot Koerkamp[1,2], Terezinha de Souza[1,2], Willemijn B Breunis[1,3], Ewa Frazer-Mendelewska[1,2], Mariël Brok[1,2], Jeff DeMartino[1,2], Freek Manders[1,2], Camilla Calandrini[1,2], Hinri H D Kerstens[1], Alex Janse[1], M Emmy M Dolman[1,4,5], Selma Eising[1], Karin P S Langenberg[1], Marc van Tuil[1], Rutger R G Knops[1], Sheila Terwisscha van Scheltinga[1], Laura S Hiemcke-Jiwa[1], Uta Flucke[1], Johannes H M Merks[1], Max M van Noesel[1], Bastiaan B J Tops[1], Jayne Y Hehir-Kwa[1], Patrick Kemmeren[1,6], Jan J Molenaar[1], Marc van de Wetering[1,2], Ruben van Boxtel[1,2], Jarno Drost[1,2,*] & Frank C P Holstege[1,6,**]

Abstract

Rhabdomyosarcomas (RMS) are mesenchyme-derived tumors and the most common childhood soft tissue sarcomas. Treatment is intense, with a nevertheless poor prognosis for high-risk patients. Discovery of new therapies would benefit from additional preclinical models. Here, we describe the generation of a collection of 19 pediatric RMS tumor organoid (tumoroid) models (success rate of 41%) comprising all major subtypes. For aggressive tumors, tumoroid models can often be established within 4–8 weeks, indicating the feasibility of personalized drug screening. Molecular, genetic, and histological characterization show that the models closely resemble the original tumors, with genetic stability over extended culture periods of up to 6 months. Importantly, drug screening reflects established sensitivities and the models can be modified by CRISPR/Cas9 with *TP53* knockout in an embryonal RMS model resulting in replicative stress drug sensitivity. Tumors of mesenchymal origin can therefore be used to generate organoid models, relevant for a variety of preclinical and clinical research questions.

Keywords rhabdomyosarcoma; mesenchymal; tumoroid; drug screening; CRISPR/Cas9
Subject Categories Cancer; Methods & Resources
DOI 10.15252/emmm.202216001 | Received 11 March 2022 | Revised 11 July 2022 | Accepted 12 July 2022 | Published online 2 August 2022
EMBO Mol Med (2022) 14: e16001

Introduction

Rhabdomyosarcoma (RMS) is a type of malignant tumor of mesenchymal origin (Yang *et al*, 2014) and forms the most common soft tissue sarcoma in children and adolescents (Li *et al*, 2008). Historically, RMS has been divided into two main subtypes based on histology. Whereas embryonal RMS (eRMS) displays cellular heterogeneity and hallmarks of immature skeletal myoblasts (Patton & Horn, 1962), alveolar RMS (aRMS) cells are distributed around an open central space, thereby resembling pulmonary alveoli (Enterline & Horn, 1958). eRMS is more frequently observed in children under 10, accounting for two-thirds of all RMS cases, and generally has a better prognosis than aRMS, which is more common in adolescents and young adults (Perez *et al*, 2011). In aRMS, a sole genetic driver alteration is usually observed, caused by a chromosomal translocation resulting in a fusion gene between either *PAX3* or *PAX7* and *FOXO1*. In contrast, eRMS is genetically more heterogeneous, harboring mutations in several common oncogenes or tumor suppressor genes (Shern *et al*, 2014). Other subtypes of RMS have recently been recognized (WHO, 2020). RMS treatment is guided by protocols developed by multinational collaborative groups and includes systemic chemotherapy in addition to local therapy (radiotherapy and/or surgery; Skapek *et al*, 2019). The prognosis of RMS has improved over the last decades (Bisogno *et al*, 2019). For patients with high-risk, refractory, or relapsed disease, prognosis remains poor however, despite an immense treatment burden (Pappo *et al*, 1999; Mascarenhas *et al*, 2019). Thus, development of new therapeutic options is of critical importance for these patients.

1 Princess Máxima Center for Pediatric Oncology, Utrecht, The Netherlands
2 Oncode Institute, Utrecht, The Netherlands
3 Department of Oncology and Children's Research Center, University Children's Hospital Zürich, Zürich, Switzerland
4 Children's Cancer Institute, Lowy Cancer Centre, UNSW Sydney, Kensington, NSW, Australia
5 School of Women's and Children's Health, Faculty of Medicine, UNSW Sydney, Kensington, NSW, Australia
6 Center for Molecular Medicine, UMC Utrecht and Utrecht University, Utrecht, The Netherlands
*Corresponding author. Tel: +31 88 9727684; E-mail: j.drost@prinsesmaximacentrum.nl
**Corresponding author. Tel: +31 88 972 72 72; E-mail: f.c.p.holstege@prinsesmaximacentrum.nl

© 2022 The Authors. Published under the terms of the CC BY 4.0 license

Development of such treatment options requires *in vitro* models and may therefore benefit from application of organoid technology. The basis of this technology is that given a suitable growth environment, tissue stem cells self-renew as well as give rise to natural progeny which organize according to their preferred growth modality without the need for artificial cell immortalization. The technology was first established in healthy epithelial tissue from mouse small intestine (Sato *et al*, 2009) and soon adapted to various other healthy and diseased epithelial tissues, including cancer (Clevers, 2016). Tumor organoid (tumoroid) systems are proving useful in cancer research as they display genetic stability over extended culture periods, retaining the molecular characteristics of the tumor they are derived from. While dedicated co-culturing tumoroid systems of tumor and nontumor cells are starting to be developed (Yuki *et al*, 2020), the majority of tumoroid systems consist only of tumor cells. Tumoroid models can be expanded, facilitating high-throughput screening approaches such as small molecule or CRISPR/Cas9-knockout screening (Bleijs *et al*, 2019).

To date, tumoroid approaches have been primarily applied to cancers derived from epithelial cells (i.e., carcinomas). Recent studies demonstrate that deriving tumoroid models from nonepithelial cancer is feasible but this has as yet not been achieved for pure mesenchymal cancers (Fusco *et al*, 2019; Jacob *et al*, 2020; Saltsman *et al*, 2020; Abdullah *et al*, 2021; Yamazaki *et al*, 2021). Application to tumors of mesenchymal origin such as RMS would be of obvious benefit. Tumoroid models of pediatric nephroblastoma (Wilms' tumor) have been described, which, depending on the subtype, can contain stromal cells (Calandrini *et al*, 2020). In addition, cells derived from synovial sarcoma and other adult soft tissue sarcomas can grow to a limited extent on fetal calf serum, which, although undefined in terms of the required essential growth factors, also indicates feasibility (Brodin *et al*, 2019; Boulay *et al*, 2021). Furthermore, *in vitro* propagation of RMS tumor cells derived from patient-derived xenograft (PDX) mouse models has recently been shown (Manzella *et al*, 2020). Although these results are encouraging, no directly patient-derived collection of tumoroid models of malignant tumors of pure mesenchymal origin (i.e., sarcomas) has been generated and studied after growth for extensive periods in well-defined media components. In this study, we therefore set out to develop and apply approaches for generating a collection of tumoroid models that covers the major RMS subtypes, a pediatric cancer of mesenchymal origin with poor outcome for high-risk patients. Besides generating and extensively characterizing the tumoroid collection, we also investigated applicability for drug screening and genetic modification (Fig 1A).

Results

A protocol to collect and process RMS tumor samples for tumoroid model establishment and propagation

Before starting to generate a collection of RMS tumoroid models, we first optimized sample acquisition and logistics between surgery, pathology, and organoid culture labs (Materials and Methods). In parallel to optimizing sample acquisition, we also optimized sample processing, including testing different formulations of growth media by a combination of systematic and trial and error approaches (Discussion). RMS tumor samples are diverse. Most samples are small needle biopsies (i.e., 16-gauge tru-cut), as large resection specimens are mostly restricted to pretreated RMS or to treatment-naïve paratesticular fusion-negative eRMS (FN-eRMS). In addition, a subset of samples (4% here) are not solid, being acquired as bone marrow aspirates of infiltrating tumor cells (Fig 1B). Samples are plated as minced pieces embedded in a droplet of extracellular matrix (ECM) substitute (Basement-Membrane Extract, BME) and as single-cell suspensions in BME-supplemented medium. Outgrowth of tumor cells to tumoroid models can occur from both modalities. In the case of successful outgrowth of initially plated cells, cells organize as two-dimensional monolayers (Fig 1C). This appears to be the cells' preferred growth modality, as plating them as single-cell suspensions in BME droplets results in cells escaping the surrounding matrix and sinking to the bottom of the culture plate from which they continue to grow in a monolayer. Therefore, cells are further propagated and expanded in this way. We considered an RMS tumoroid model to be successfully established if, over the course of culturing, the expression of specific tumor markers is retained and the culture expansion is at least sufficient for drug screening, all as described below (Table 1).

Early detection of tumor cells during culturing

Tumors consist of a variety of different cell types. These include normal cell types that can grow as well or even better in the provided culture conditions, possibly outcompeting tumor cells (Dijkstra *et al*, 2020). It would therefore be useful to test for the presence of tumor cells early during culturing to omit the unnecessary propagation of cultures lacking any. At early time points, material is limited, impacting the range of applicable assays. The establishment protocol therefore utilizes an RT-qPCR assay after the first or second passage of cells with probes for standard RMS histopathology markers, that is, *DES*, *MYOG*, *MYOD1* (WHO, 2020), and the fusion transcript in fusion-positive RMS (FP-RMS; Ponce-Castañeda *et al*, 2014). We considered a sample positive for tumor cells if at least one of the three genes, plus for FP-RMS the fusion transcript, tests positive. All samples that successfully yield tumoroid models, show positivity for at least one marker gene at this stage, while most models (17 out of 19) are positive for all three marker genes and the fusion transcript if applicable (Fig 2A and B). The RT-qPCR-based approach is therefore a useful tool to determine feasibility at an early stage.

RMS tumoroid models retain marker protein expression and display heterogeneity in gene expression

A hallmark of RMS tumors is the expression of proteins associated with nonterminally differentiated muscle (i.e., Desmin, Myogenin, and MYOD1). Expression of these proteins differs between RMS subtypes (Dias *et al*, 2000) and can be associated with prognosis (Heerema-Mckenney *et al*, 2008). To properly reflect the original tumors, RMS tumoroid models should therefore retain the expression patterns of these proteins. The RMS tumoroid establishment protocol therefore includes a morphological (H&E) and immunohistochemical (IHC) assessment at the time of successful establishment (i.e., drug screening). To enable comparison between tumoroid models and the tumors they were derived from, models are grown

 © 2022 The Authors

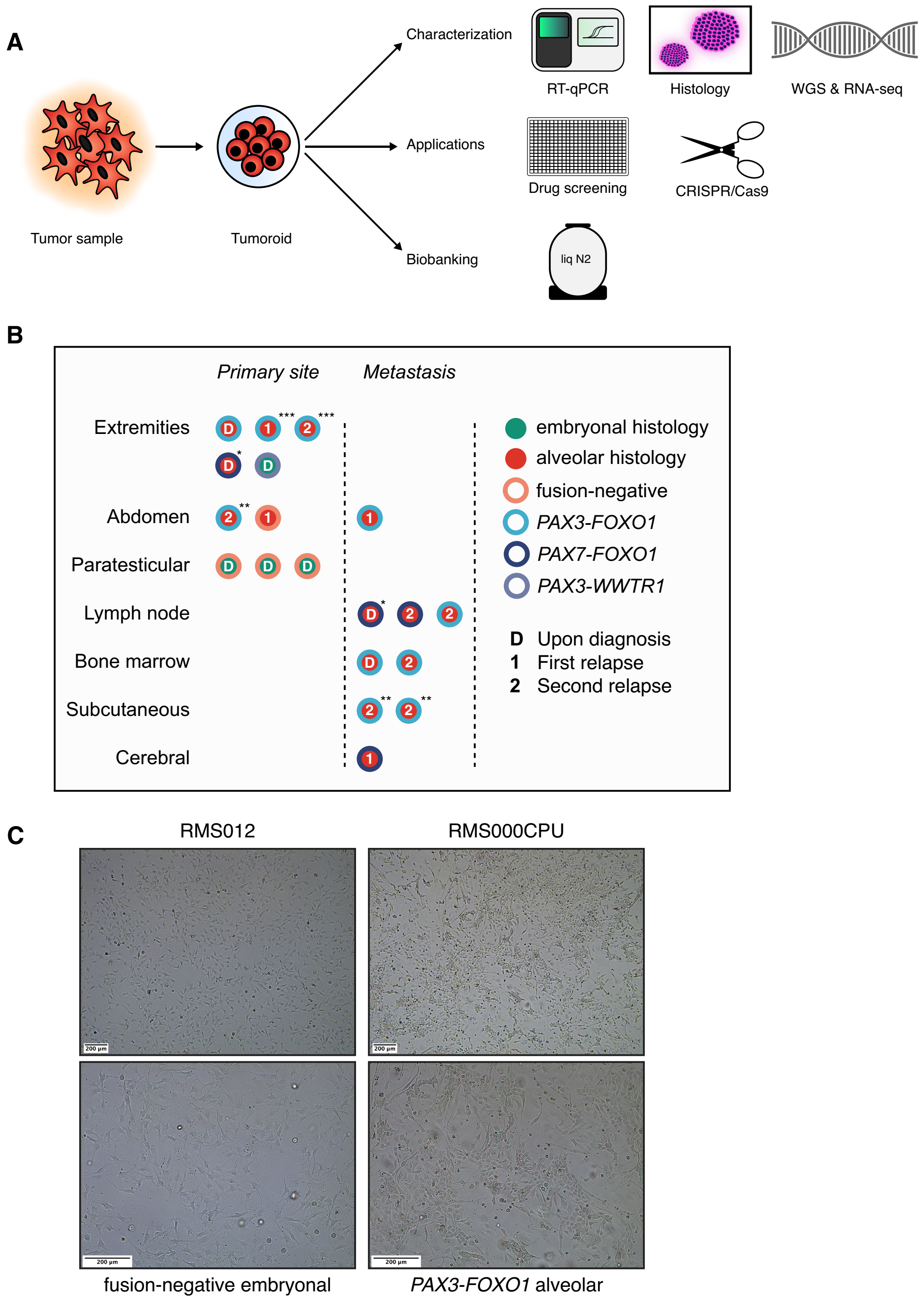

Figure 1. A collection of RMS tumoroid models that represent the diverse clinical presentation of RMS.

A Tumor organoid (tumoroid) pipeline. WGS, whole-genome sequencing; RNA-seq, mRNA sequencing; liq N2, liquid nitrogen.

B Overview of available RMS tumoroid models in the collection separated by primary versus metastatic site and exact tumor location. The color of the inner circle indicates the histological subtype while the color of the outer circle indicates the presence or absence of a fusion transcript. Letters within the circle indicate disease instance. Asterisks mark tumoroid models derived from the same patient but from distinct tumor samples.

C Brightfield microscopy images of two representative RMS tumoroid models from a fusion-negative embryonal and a *PAX3-FOXO1* fusion-positive alveolar tumoroid model grown in a two-dimensional monolayer in two magnifications as indicated by the scale bars.

© 2022 The Authors

Table 1. Overview of available RMS tumoroid models in the collection. Patients are numbered to allow identification of RMS tumoroid models derived from the same patient. For additional clinical parameters see Dataset EV1.

Tumoroid model	Patient	Patient birth year	Histology	Fusion transcript	Patient sex	Disease instance of tumoroid establishment	Body site of sample used for tumoroid establishment
RMS007	1	2011	Alveolar	negative	Male	First relapse	Abdomen
RMS102	2	2000	Alveolar	*PAX3-FOXO1*	Male	Second relapse	Lymph node metastasis (clavicle)
RMS410	3	2001	Alveolar	*PAX3-FOXO1*	Male	Second relapse	Bone marrow metastasis
RMS000CPU	4	2001	Alveolar	*PAX3-FOXO1*	Male	First relapse	Kidney
RMS127	5	2003	Alveolar	*PAX3-FOXO1*	Male	Primary disease	Bone marrow metastasis
RMS006	6	2004	Alveolar	*PAX3-FOXO1*	Male	First relapse	Calf
RMS013	6	2004	Alveolar	*PAX3-FOXO1*	Male	Second relapse	Calf
RMS108	7	2004	Alveolar	*PAX3-FOXO1*	Female	Second relapse	Bladder
RMS109	7	2004	Alveolar	*PAX3-FOXO1*	Female	Second relapse	Subcutaneous metastasis
RMS110	7	2004	Alveolar	*PAX3-FOXO1*	Female	Second relapse	Subcutaneous metastasis
RMS000FLV	8	2018	Alveolar	*PAX3-FOXO1*	Female	Primary disease	Arm
RMS335	9	2010	Alveolar	*PAX7-FOXO1*	Female	Second relapse	Lymph node metastasis (groin)
RMS000HQC	10	2011	Alveolar	*PAX7-FOXO1*	Male	First relapse	Cerebral metastasis
RMS000HWO	11	2014	Alveolar	*PAX7-FOXO1*	Female	Primary disease	Leg
RMS000HWQ	11	2014	Alveolar	*PAX7-FOXO1*	Female	Primary disease	Lymph node metastasis (groin)
RMS444	12	2003	Embryonal	Negative	Male	Primary disease	Paratesticular
RMS012	13	2013	Embryonal	Negative	Male	Primary disease	Paratesticular
RMS000ETY	14	2014	Embryonal	Negative	Male	Primary disease	Paratesticular
RMS000EEC	15	2005	Embryonal	*PAX3-WWTR1*	Male	Primary disease	Shoulder

as three-dimensional spheres to mimic the three-dimensional architecture of tumors (Fig 2C). When evaluated in this manner, RMS tumoroid models show retained expression patterns of Desmin, Myogenin, and MYOD1 at the time of successful establishment (i.e., drug screening), comparable to parental tissue. Cellular morphology is also retained, with aRMS models displaying homogenous, primitive cells, with large nuclei, and minimal cytoplasm, and eRMS models displaying more heterogeneous tumor cells with variable maturation (Figs 2C and EV1A), as in the tumors (, 2020). Lastly, additional H&E and IHC staining performed on two different passages of two tumoroid models (i.e., after the acquisition of the tumor sample and before the drug screening was performed;

Figure 2. Early detection of tumor cells during culturing, retained marker protein expression and heterogeneity in gene expression.

A RT-qPCR of early passage RMS tumoroid models shows positivity for at least one gene used in standard-of-care pathology analysis (*DES*, *MYOG*, or *MYOD1*). Conventional RMS cell lines (RD and RH30) were used as positive controls, while two Synovial Sarcoma (SS000DAZ and SS077) tumoroid models were used as negative controls. Gene expression was normalized to the expression of a house-keeping gene and human reference RNA (HREF) via the ΔΔCq method. Each tumoroid line was measured once with four technical replicates with the error bars representing the standard deviation of said technical replicates.

B RT-qPCR of early passage RMS tumoroid models reliably detects the aberrant fusion transcripts. Fusion gene expression was normalized to the expression of a house-keeping gene via the ΔCq method. Each tumoroid line was measured once with four technical replicates with the error bars representing the standard deviation of said technical replicates.

C Morphological (via H&E) and immunohistochemical (IHC) comparison of RMS tumors and derived RMS tumoroid models shows retained marker protein (Desmin, Myogenin and MYOD1) expression and cellular morphology. Scale bars equal 200 μm (RMS012, RMS102) or 100 μm (RMS000HQC).

D t-SNE projection of single-cell transcriptomes from the RMS127 and RMS444 tumoroid models. Plots on the right show the normalized expression values, per single cell, of *MYOG*, *MYOD1*, *DES*, and *MKI67*, respectively.

Source data are available online for this figure.

© 2022 The Authors

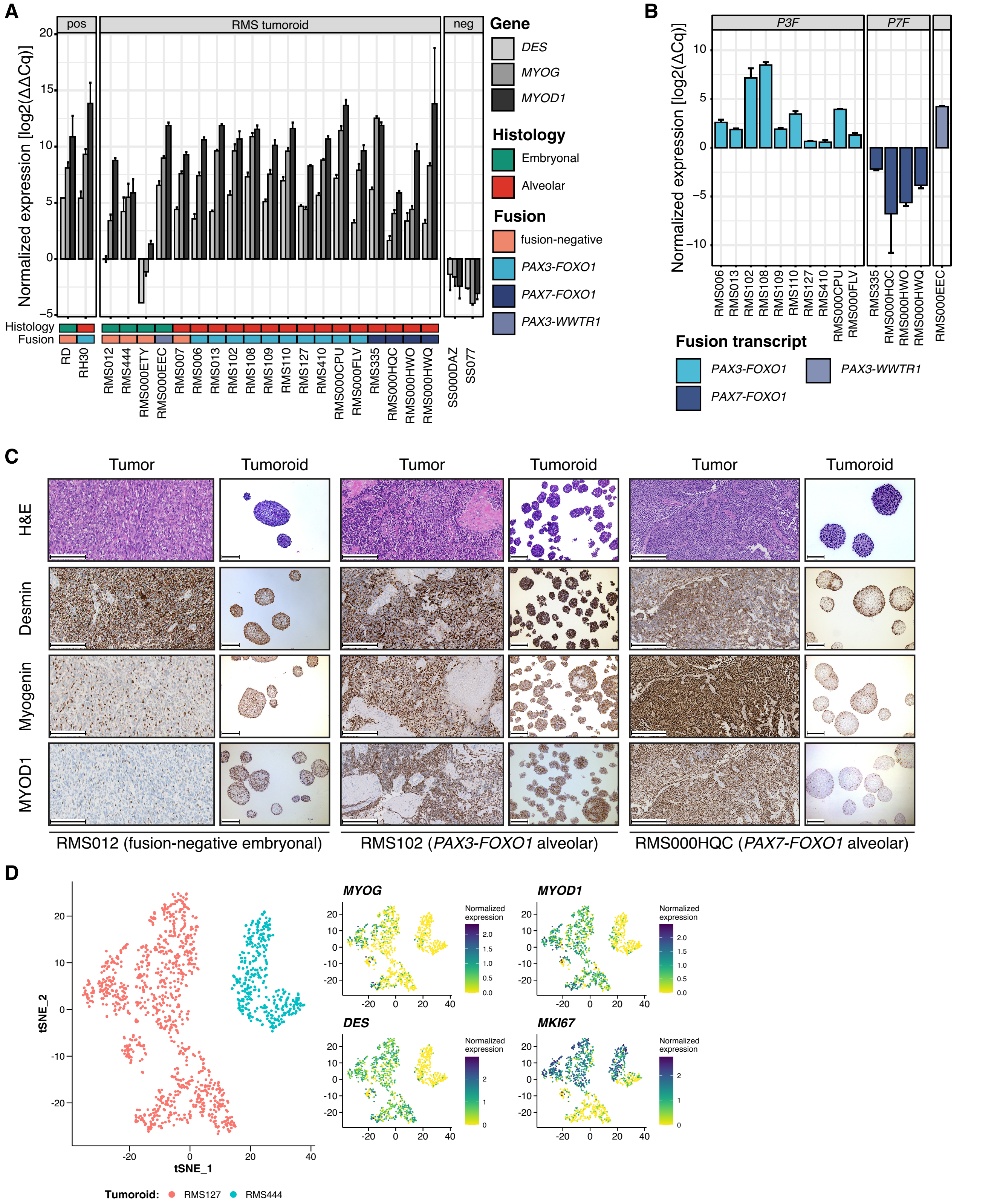

Figure 2.

Fig EV2A) show a high concordance concerning the heterogeneity of marker protein expression when compared to the primary tumor and the tumoroid model at the time of drug screening (Fig 2C). This indicates that there are no major changes concerning the composition of tumor cells acquired over the course of expanding the culture for drug screening.

In line with the heterogeneous expression of these marker proteins, single-cell RNA sequencing (scRNA-seq) shows that RMS tumoroid models retain heterogeneity in the expression of the corresponding genes (i.e., *MYOG*, *MYOD1*, and *DES*; Fig 2D). Importantly, this heterogeneity is not due to differences in cell cycle activity as differential gene expression patterns do not correspond with the expression of the cell cycle marker *MKI67* (Fig 2D). Single-cell RNA-seq inferred per cell copy-number plots also suggest genomic heterogeneity, and furthermore show that RMS tumoroid models consist only of tumor cells as all analyzed cells in the samples show copy-number alterations in agreement with whole-genome sequencing inferred copy-number plots (Fig EV2B). In summary, RMS tumoroid models retain histopathological hallmarks of RMS tumors as well as display transcriptional heterogeneity in line with heterogeneous protein expression at the time of drug screening, giving a first indication that they reflect the tumors they were derived from to a high extent.

A collection of RMS tumoroid models that represent the diverse clinical presentation of RMS

Having established protocols for the acquisition, processing, initial growth, and characterization of RMS tumoroid models, we applied this to 46 consecutive samples from pediatric RMS patients treated in the Netherlands, resulting in a collection of 19 RMS tumoroid models (41% efficiency) (Table 1). These models are derived from tumors comprising both main histological subtypes (embryonal and alveolar), different fusion types (fusion positive *PAX3-FOXO1*, *PAX7-FOXO1*, a novel fusion *PAX3-WWTR1*, as well as fusion negative), various risk groups, locations, and are derived from primary as well as relapsed disease (Fig 1B and Dataset EV1). Outgrowth of cells from highly aggressive RMS subtypes shows a higher success rate, as indicated by 83% successful establishment for FP-RMS versus 16% for FN-RMS, and 61% success in relapsed, versus 30% in primary RMS tumors, respectively. There was no statistically significant difference in the success rate between recently treated and untreated tumors (33% versus 43%, respectively, $P = 0.59$, chi-square test). Similar differences between relapsed and primary tumors in establishment rates have been observed for orthotopic PDX models (O-PDX) of RMS (Stewart *et al*, 2017). Compared to O-PDX models of RMS, the overall success rate is lower for establishing RMS tumoroid models (41% versus 65%, respectively). While the success rate is lower, RMS tumoroid models can be more rapidly established. Engraftment of O-PDX RMS models takes 1–5.5 months before tumor growth is first observed in mice, while RMS tumoroid models can be fully established, that is, characterized and subjected to drug screening, in as little as 27 days for highly aggressive tumors. Overall, the median time from acquisition of the tumor sample to successful drug screening was 81 days (with seven models being screened in less than 2 months). This indicates that our approach could potentially be applied in a personalized medicine setting where it is crucial to obtain results as fast as possible to provide information on treatment options.

A subset of the tumoroid models were derived from the same patient, but at different points during treatment and/or from different body sites (marked with one or more asterisks in Fig 1B). This potentially facilitates studies of tumor evolution or acquired treatment resistance. Furthermore, the collection contains a model of an eRMS with a fusion between *PAX3* and *WWTR1*, which has not previously been described in RMS. Such fusions have been reported as rare events in biphenotypic sinonasal sarcomas, which usually harbor *PAX3-MAML3* fusions (Le Loarer *et al*, 2019). Taken together, the protocol efficiently yields tumoroid models from highly aggressive as well as from extremely rare RMS subtypes, resulting in an initial collection covering a broad spectrum of subtypes.

RMS tumoroid models molecularly resemble the tumor they are derived from

Given that the present tumoroid collection is the first to be established from tumors of purely mesenchymal origin, we asked to what extent the models further resemble the tumors they were derived from besides the retained hallmark protein expression levels and patterns described above. To this end, whole-genome sequencing (WGS) and bulk mRNA sequencing (RNA-seq) of the tumor and tumoroid model at the time of drug screening (i.e., successful establishment), were performed.

Copy number profiles were first compared between tumors and tumoroid models showing that profiles are indeed highly concordant (Fig 3A). The detected copy number alterations included those commonly observed in RMS, with genomic gains in chromosome 8 in FN-RMS and gains in chromosome 1 and 12 in FP-RMS (Weber-Hall *et al*, 1996; Shern *et al*, 2014). In addition, copy number profiles of individual RMS tumors and derived tumoroid models show a high concordance (Fig EV3A and B). This indicates that the established models resemble the tumors they were derived from on a more global genomic level.

Various mutational processes are active in cells, which cause distinct somatic mutational signatures. These signatures are characterized by specific patterns of single base substitutions (SBS) in the context of their two flanking bases (Alexandrov *et al*, 2020). The presence of certain somatic mutational signatures in a cell can be associated with the underlying mutational processes. These processes are not restricted to *in vivo* systems, but can also occur during culture (Petljak *et al*, 2019), forming a potential source of genomic destabilization. To test whether the somatic mutational signatures and thus the underlying mutational processes present in the tumors ("T") are concordant with those in the derived tumoroid models ("O"), we first measured the relative contributions of different signatures per sample. The main signatures observed are signatures associated with cellular aging (SBS1 and SBS5), a signature associated with increased oxidative stress (SBS18; Alexandrov *et al*, 2020), and a signature associated with exposure to the chemotherapeutic Temozolomide (TMZ; Kucab *et al*, 2019) used in the treatment of RMS (Defachelles *et al*, 2021; Fig 3B). Signatures associated with cellular aging (SBS1 and SBS5) show a significant correlation with patient age only for SBS1 and only in RMS tumoroid models ($P = 0.02$) but not tumors ($P = 0.17$). Furthermore, FP-RMS tumors and tumoroid models show a significantly higher contribution of SBS1 (but not SBS5) to their overall somatic mutation frequency compared to FN-RMS tumors and tumoroid models ($P = 0.02$ and $P = 0.006$, respectively). However, patients with FP-RMS tumors were older than patients with FN-RMS tumors (median 14 years versus median 6 years, respectively), which may have influenced this. Importantly, the signatures detected in the tumoroid models are highly concordant with those detected in the original

 © 2022 The Authors

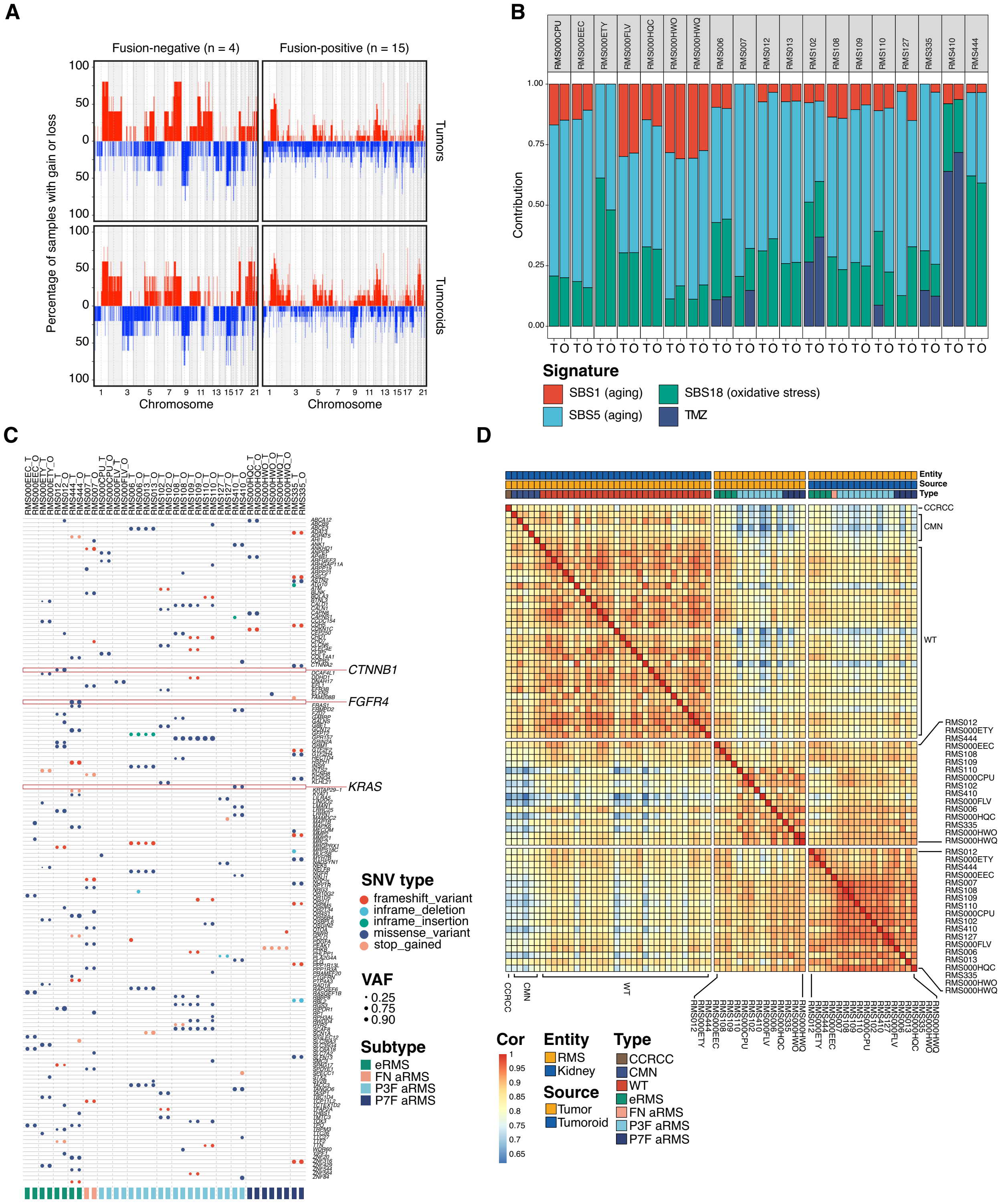

Figure 3. RMS tumoroid models molecularly resemble the tumor they are derived from.

A Copy number frequency plots of RMS tumors (upper row) and derived RMS tumoroid models (lower row) divided by fusion-status (columns). Chromosomes are annotated on the *x*-axis from left to right while the *y*-axis shows the percentage of samples in this group carrying a gain (red) or loss (blue) in this genomic region.

B Contribution of somatic mutational signatures per tumor and tumoroid model. SBS, single base substitution; TMZ, temozolomide; T, tumor; O, tumoroid.

C Table depicting pathogenic single-nucleotide variants (SNVs) in RMS tumors (T) and tumoroid models (O). Circle color indicates SNV type while circle size indicates variant allele fraction (VAF). Vertical dotted lines separate samples derived from individual patients. Highlighted are genes previously reported for this RMS subtype.

D Correlogram of bulk mRNA sequencing expression profiles of pediatric kidney tumors (controls) as well as RMS tumoroid models and RMS tumors. CCRCC, Clear Cell Renal Cell Carcinoma; CMN, Congenital Mesoblastic Nephroma; WT, Wilms' Tumor; Cor, correlation.

tumors (Fig 3B). In line with this, no global differences in SBS profiles between RMS tumors and derived tumoroid models are detected when analyzed collectively (Fig EV3C). Lastly, we calculated the similarities of the individual SBS profiles of all tested tumor and tumoroid model samples. Tumoroid models cluster with the tumors they are derived from, indicating that the mutational landscape is retained in the models (Fig EV3D). Only the tumor and tumoroid model of RMS127 do not cluster closely, likely due to derivation from a bone marrow aspirate with low tumor cell infiltration (5–10% as estimated by pathology). Overall, there is a high concordance between the somatic mutational signatures of RMS tumors and the derived tumoroid models, again illustrating that the established models resemble the patient tumors.

While FP-RMS are driven by the prototypical fusion genes, FN-RMS are characterized by SNVs in known oncogenes such as *TP53* or *RAS* family members (Shern *et al*, 2014). Currently available preclinical models of RMS do not cover the full spectrum of these SNVs, thus limiting the applicability to test novel targeted drugs in RMS. To investigate whether the established RMS tumoroid models harbor any of these SNVs and whether these were already present in the tumor, all SNVs predicted to be pathogenic for protein function and with a variant allele fraction (VAF) of above 0.3 in either tumor and/or tumoroid were evaluated. As already indicated by the mutational signature analyses, RMS tumoroid models retain SNVs present in the tumor to a high degree (Fig 3C). Similarly, samples from the same patient but from different sites or acquired from different relapses also show a high overlap in SNVs (Fig 3C). In agreement with a recent study investigating mutational frequency in different tumor types (Casey *et al*, 2020), the FN-RMS tumoroid models show a higher somatic mutation frequency than the FP-RMS tumoroid models ($P = 0.02$; Fig EV3E) indicating that the models are representative for this characteristic, as well.

Two of the FN-eRMS tumoroid models harbor previously described oncogenic mutations for FN-eRMS, that is, *CTNNB1* (p.T41A) in RMS012 and *FGFR4* (p.V550L) in RMS444 (Shern *et al*, 2014). To our knowledge, RMS012 is the first preclinical RMS model harboring this specific mutation. Moreover, one *PAX3-FOXO1* FP-aRMS (RMS410) displays an oncogenic mutation in *KRAS* (p.G12A), which is uncommon in FP-RMS (Shern *et al*, 2014). Lastly, two FN-RMS tumoroid models (RMS007 and RMS012) display non-annotated frameshift mutations in the *BCOR* gene with a high VAF of above 0.9. Mutations in *BCOR* have been reported in RMS with a higher prevalence in FN-RMS compared to FP-RMS (Shern *et al*, 2014). The mutation analysis shows that RMS tumoroid models not only retain specific SNVs already present in the tumor but that these models also contain mutations for which no preclinical model was previously available.

Tumors are composed of different tumor cell clones that can undergo processes such as clonal expansion, genetic diversification as well as clonal selection (Greaves & Maley, 2012). To assess to what degree established RMS tumoroid models reflect the clonal composition of the tumor they were derived from, we performed two analyses on the matching WGS data.

First, we compared the VAFs of SNVs in coding regions which showed no copy-number alterations or loss of heterozygosity (LOH; Fig EV4A). For the majority of samples, SNVs with a VAF of around 0.5 (indicating a major clone in the population given the filtering criteria for SNVs described above) can be detected in tumor and tumoroid, indicating that major clones are retained. Furthermore, the presence of SNVs with VAFs of below 0.5 indicates subclonal populations. Again, such SNVs with matching VAFs in tumor and tumoroid samples can be detected, indicating that subclonal populations are also largely retained in the established RMS tumoroid models. In addition, a subset of SNVs present in the tumoroid with a high VAF is not present in the tumor with the chosen cut-offs, indicating that our method may enrich for such clones. Importantly, none of these SNVs are found in reported oncogenic driver genes, indicating that these are likely passenger mutations.

In the second analysis performed on the matching WGS data, fractions of alternate alleles (B-alleles) were compared between matching tumors and tumoroid models (Fig EV4B). Importantly, patterns present in the tumor are retained in the derived tumoroid, indicating that the relative contributions of clones with varying B-allele fractions are retained in the established models. Minor shifts between the peaks are likely the result of the tumor sample being impure, that is, containing normal cells, whereas the tumoroid models consist purely of tumor cells.

Taken together, these analyses indicate that established RMS tumoroid models maintain the genetic characteristics including the clonal composition of the RMS tumor they were derived from to a large extent.

mRNA expression profiles of FN- and FP-RMS are fundamentally different, mainly due to the transcriptome-wide impact of the fusion transcript in FP-RMS (Wachtel *et al*, 2004). Additionally, the transcriptional program of *in vitro* cultured organoids is influenced by the culture conditions and can deviate from the transcriptional program of the parent tissue (Lu *et al*, 2021). Given that FN-RMS and FP-RMS tumoroid models are cultured in the same medium, we asked whether the transcriptional differences observed between the original tumors are retained in the models. Analysis of RNA-seq shows a high correlation between the expression profiles of RMS tumoroid models with the same fusion status (i.e., FP-RMS versus FN-RMS, Fig 3D), as has previously been shown for primary RMS tumor samples (Wachtel *et al*, 2004). Furthermore, correlation between the expression profiles of RMS tumor and tumoroid models of the same fusion-type is high, while correlation with control kidney tumors is low, showing that the fundamental differences in the expression profiles of FN- and FP-RMS are retained in culture (Fig 3D). In summary, the early tumor cell detection by RT-qPCR, the morphological and marker protein analyses, WGS for copy number profiles, for somatic mutational signatures, for individual SNVs, and for assessing the clonal composition, as well as the comparative transcriptomic analyses, indicate that the RMS tumoroid models resemble the original RMS tumor they are derived from.

Genetic and transcriptional stability of tumoroid models over time

We next asked whether the models remain genetically and transcriptionally stable after culture over extended periods. To investigate stability, a subset of RMS tumoroid models were kept in culture over 3–6 months until they reached passage 40 ("OL" for late passage, as compared to "O", the standard passage analyzed here and sufficient for drug screening) and characterized again by WGS and RNA-seq. Furthermore, two independently derived tumoroid models (i.e., established from the same tumor piece of which a part was

© 2022 The Authors

cryo-preserved after the first tumoroid establishment) were included in this comparison ("O2") to assess the robustness of the establishment protocol.

RMS tumoroid models show long-term propagation potential with five out of seven lines tested reaching passage 40 and two lines dropping out at passages 17 and 20, respectively. As drug screening is usually performed between passages 6 and 12, this shows that models can be readily used after such an initial screening. Comparison of individual copy number profiles of standard and late passage (Fig EV5A), as well as independently derived paired tumoroid models (Fig EV5B) shows no major copy number differences between the respective models. Furthermore, analysis of somatic mutational signatures shows that the contributions of identified somatic mutational signatures (Fig 4A) as well as the contributions of individual SBSs between models (Fig EV5C) are highly similar. Lastly, analysis based on SBS profiles shows clustering of models derived from individual patients (Fig EV5D). The exception to this is RMS335 "OL" which may be due to a mutation in the DNA damage response gene *ATR* in both the tumor and the tumoroid sample, potentially resulting in the gain of new mutations during culturing. The similarity between SBS profiles of RMS335 "O" and "OL" was nevertheless high. Taken together, these analyses show that RMS tumoroid models generally remain stable, even over extended periods of culturing and that the establishment protocol is robust, yielding highly similar models when independently derived from a single tumor sample.

As before (see Fig 3C), individual pathogenic SNVs were also evaluated. Importantly, the majority of SNVs are retained after long-term culturing, with the models acquiring only a few additional SNVs (Fig 4B). In addition, the models independently derived from the same tumor show a high overlap of pathogenic SNVs (Fig 4B). Notably, the afore-mentioned oncogenic mutations in *CTNNB1* and *FGFR4* are detected in all related samples (Fig 4B). This further indicates that the models do not lose key mutations and that the establishment protocol results in the outgrowth of representative tumor cells harboring these mutations. RNA-seq expression profiles from the standard passage, late passage, as well as the independently derived tumoroid models were compared to test whether the core transcriptional program of RMS is retained. Principal component analysis shows that global expression profiles of tumoroid models derived from the same patient cluster together (Fig 4C). This suggests that our models are not only genetically, but also transcriptionally stable over time.

RMS tumoroid drug screening reflects established drug sensitivities

Having established that the RMS tumoroid models resemble the tumors they are derived from with stability during prolonged culturing, their suitability for research was further investigated in two specific ways, that is, via drug screening and CRISPR/Cas9 genome editing. Development of novel RMS treatments has been hampered by a lack of preclinical models that can efficiently be subjected to drug screening. To be of use for such screening approaches, it is imperative that new models reflect drug sensitivities already known for these tumors. This was investigated with a custom pediatric cancer library of 165 compounds, comprising standard of care chemotherapeutics as well as early-phase clinical trial targeted compounds. To mimic the three-dimensional configuration of tumors, cell plating was optimized so that tumoroid cells form homogenous 3D spheres in 384-well plates. The protocol also included prior growth curve determination of the number of cells that must be plated for each individual tumoroid model to prevent overgrowth during screening (Materials and Methods).

Vincristine and actinomycin D are routinely used in RMS treatment and indeed show broad efficacy in all models tested (Fig 5A, bottom highlighted box). Furthermore, the proteasome inhibitor bortezomib shows a similarly high efficacy in all tested models. This is in line with previous studies showing that both major histological RMS subtypes are susceptible to bortezomib treatment *in vitro* (Bersani *et al*, 2008) and *in vivo* (Manzella *et al*, 2020), suggesting that RMS tumoroid models indeed reflect drug sensitivities known for RMS tumors.

Clustering of RMS tumoroid models based on drug efficacy shows two main groups, comprising 12 and 4 models, respectively, with one unclustered model (RMS000FLV). This model nevertheless shows high sensitivity to the afore-mentioned drugs (bottom of Fig 5A). The outlier behavior is not caused by differences in growth during the experiment (see Table EV1) and may be explained by the fact that this is the only treatment-naïve FP-aRMS model in the collection (Fig 1B). The group of four models, that contains all successfully screened FN-RMS tumoroid models, is more sensitive to all tested inhibitors of MEK/ERK (MAPK signaling pathway) as well as the two inhibitors of γ-secretase (NOTCH signaling pathway) in the drug panel, when compared to the other group containing only FP-RMS tumoroid models (Fig 5A, top highlighted box, and Fig 5B and C). Importantly, sensitivity of FN-RMS against inhibitors of MAPK and NOTCH signaling has previously been reported (Belyea *et al*, 2011; Yohe *et al*, 2018). This group also contains the FP-aRMS tumoroid model RMS000HQC which shows a very low expression of its fusion transcript (Fig 2B), which potentially resulted in it displaying sensitivities more commonly observed in FN-RMS. Taken together, our results indicate that drug sensitivities observed in RMS tumoroid models reflect those known in primary RMS tumors. This shows the potential these models hold for testing novel drugs. As drug screening could be performed as early as 27 days after sample acquisition, with a median time to drug screening of 81 days, this highlights their relevance for personalized approaches.

RMS tumoroid models can be molecularly edited using CRISPR/Cas9

The applicability of preclinical models would be further enhanced by the possibility of genetic modification. To test this, we used CRISPR/Cas9 (Jinek *et al*, 2012) to knock out the well-known tumor suppressor gene *TP53*. This choice is based on the recent report that loss of functional P53 protein confers a worse prognosis in RMS (Shern *et al*, 2021). RMS012 FN-eRMS tumoroid cells, with wild-type *TP53* as determined by WGS, were transfected with a plasmid harboring a *TP53* targeting sgRNA as well as Cas9. Successfully edited cells were selected with nutlin-3 (Drost *et al*, 2015; Fig 6A), resulting in cells with complete loss of P53 protein as confirmed by Western Blot (Fig 6B). Sanger sequencing shows a spectrum of Indels consistent with a polyclonal population of P53-deficient cells (Fig EV6A).

© 2022 The Authors

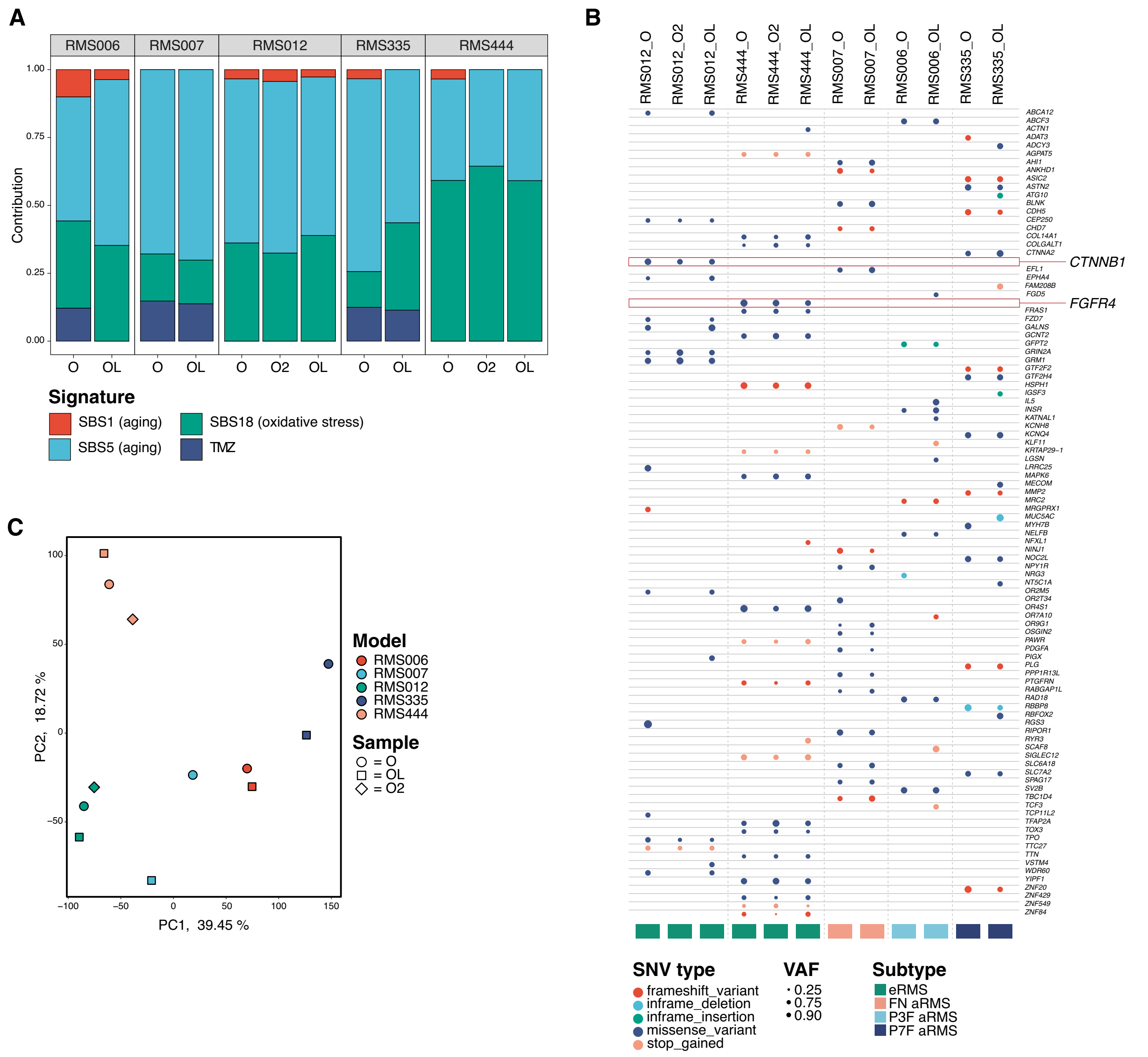

Figure 4. Genetic and transcriptional stability of tumoroid models over time.

A Contribution of somatic mutational signatures per tumor and tumoroid model. SBS, single base substitution; TMZ, temozolomide; T, tumor; O, standard passage tumoroid model; OL, late passage tumoroid model; O2, independently derived tumoroid model.

B Table depicting pathogenic single-nucleotide variants (SNVs) in different RMS tumoroid models derived from the same tumor sample. Circle color indicates SNV type while circle size indicates variant allele fraction (VAF). Highlighted are genes previously reported for this RMS subtype.

C Principal component analysis on bulk mRNA sequencing derived global gene expression. Color indicates the RMS tumoroid model while the symbol indicates the sample. O, standard passage tumoroid model; OL, late passage tumoroid model; O2, independently derived tumoroid model.

Discussion

A tumoroid collection of purely mesenchymal origin

To date, organoid technology has primarily been employed to generate models of malignant tumors of epithelial origin (i.e., carcinomas; Bleijs *et al*, 2019). Feasibility to use this technology on nonepithelial cancer has only recently been shown (Fusco *et al*, 2019; Jacob *et al*, 2020; Saltsman *et al*, 2020; Abdullah *et al*, 2021; Yamazaki *et al*, 2021). Here, we extend the tumor organoid approach, demonstrating applicability to tumors of entirely mesenchymal origin (i.e., sarcomas), resulting in only the second thoroughly characterized tumoroid collection specific for pediatric cancer.

Two factors likely contributed to the delayed adaptation of organoid technology to sarcomas. First, the technology was developed for healthy epithelial tissue, followed later by translation to the corresponding cancer entity, as in the case of colorectal carcinoma (Sato *et al*, 2009, 2011; Van De Wetering *et al*, 2015). The cell of origin of

 © 2022 The Authors

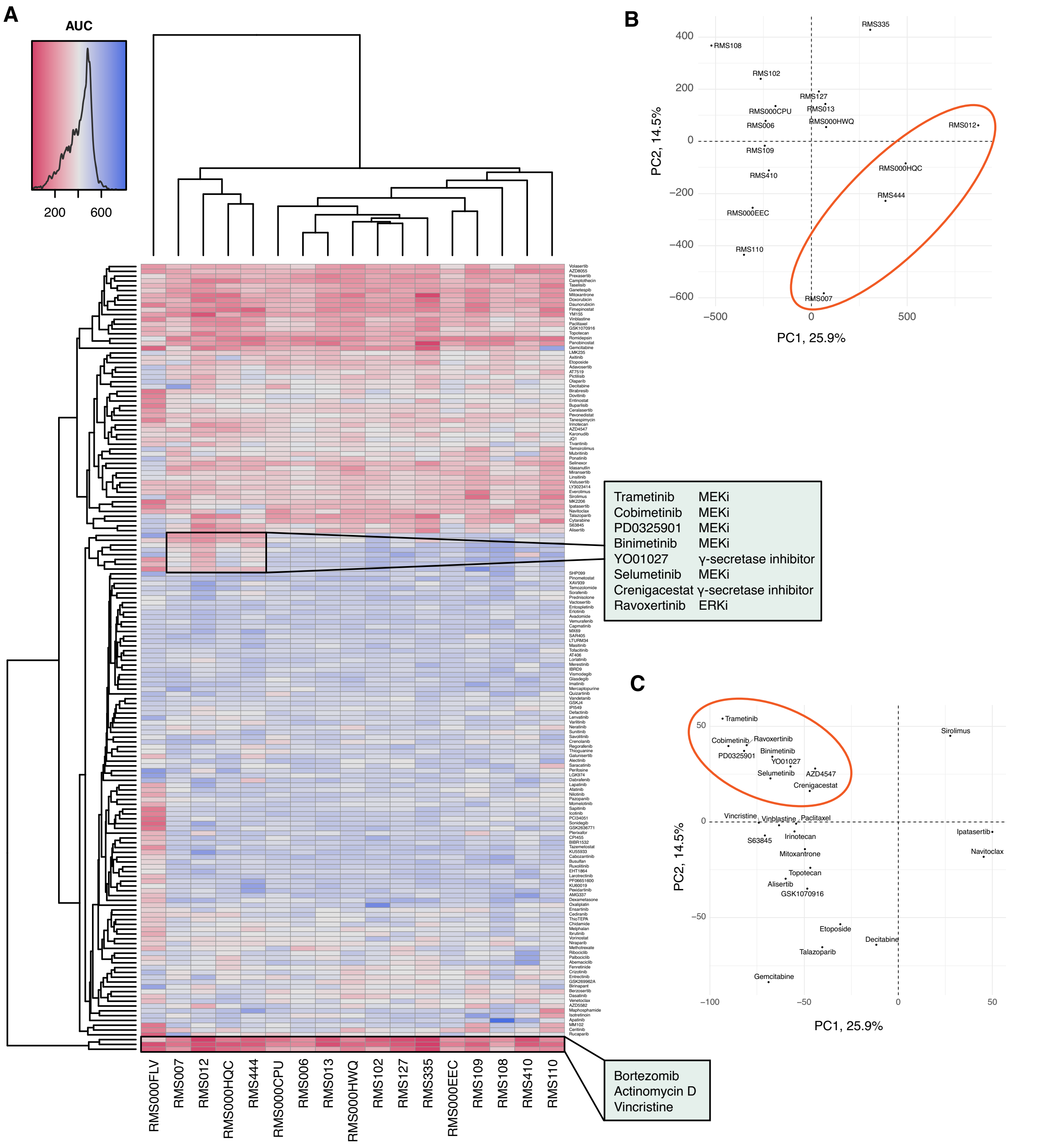

Figure 5. RMS tumoroid drug screening reflects established drug sensitivities.

A Clustered heatmap of viability measurements per RMS tumoroid model (*x* axis) and drug (*y* axis), showing the Area Under the Curve (AUC) after treatment of the cells for 120 h with a dose-range of 0.1 nM to 10 μM. Low AUC (red) indicates high drug efficacy while high AUC (blue) indicates low drug efficacy. Annotated clusters of (1) MEK/ERK and γ-secretase inhibitors showing specific efficacy in RMS tumoroid models without (RMS007, RMS012, RMS444) or low (RMS000HQC) fusion transcript expression, and (2) drugs that show broad efficacy across RMS tumoroid models.

B Principal component analysis of drug screening AUC values of the RMS tumoroid models (RMS000FLV omitted due to outlier behavior as discussed in the main text). The ellipse indicates the cluster that shows specific sensitivity against MEK/ERK and γ-secretase inhibitors.

C Principal component analysis of the top 25 contributing drugs that influence variance. The ellipse indicates the MEK/ERK and γ-secretase inhibitors as well as AZD4547 (RMS000FLV omitted as described above).

Source data are available online for this figure.

© 2022 The Authors

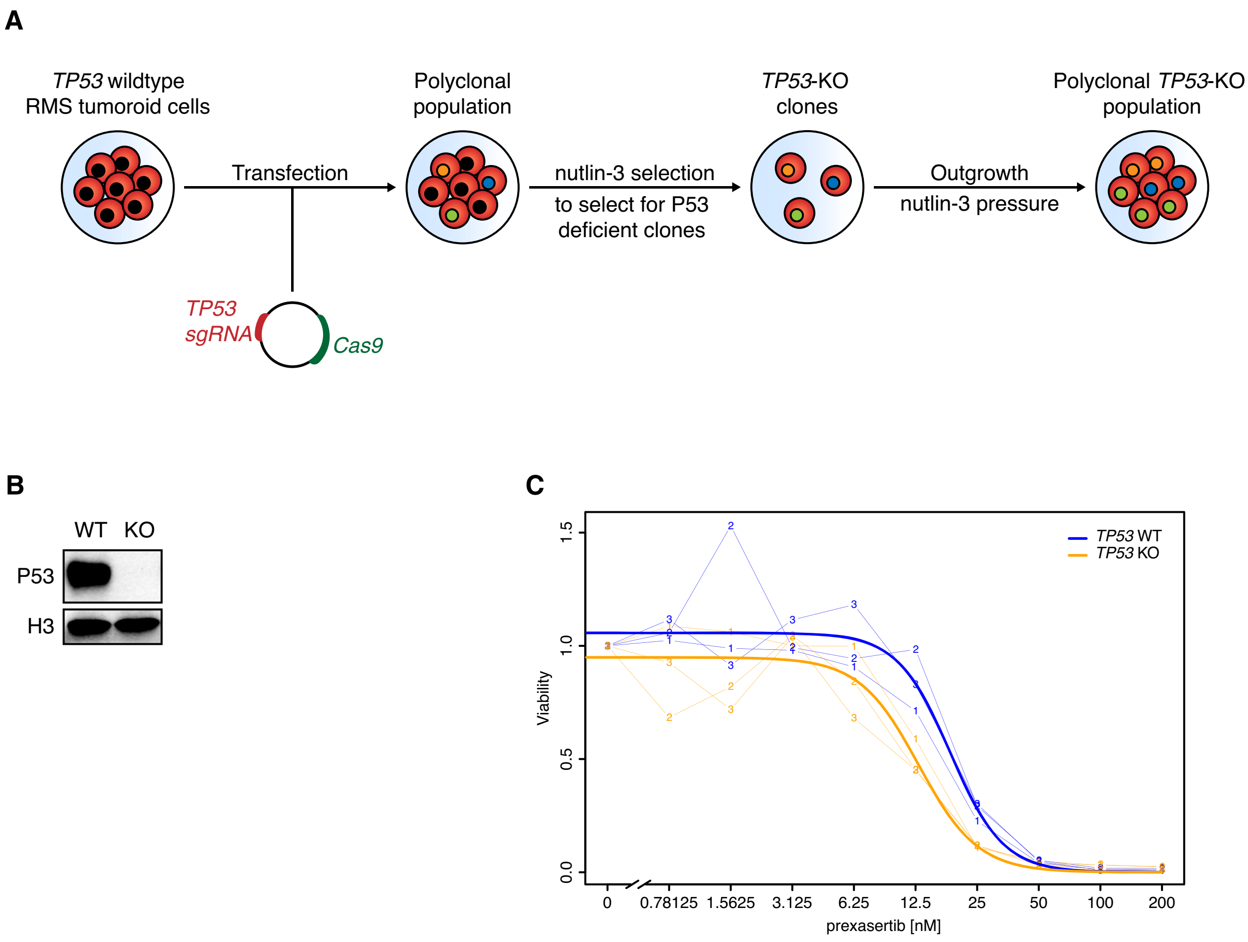

Figure 6. RMS tumoroid models can be molecularly edited using CRISPR/Cas9 with P53-deficient eRMS being more sensitive to the checkpoint kinase inhibitor prexasertib.

A Transfection and selection strategy to achieve *TP53* knockout in a *TP53* wild-type tumoroid model.

B Western Blot analysis of *TP53* wild-type (WT) and knockout (KO) RMS tumoroid line RMS012. Histone 3 (H3) served as loading control.

C Dose-response curve of *TP53* WT and KO cells treated with the Chk-1 inhibitor prexasertib. Thin lines with numbers indicate individual biological replicates (n = 3) while thick lines indicate fitted lines over all replicates. The statistical significance of the differences in fitted IC_{50} values between WT and KO were obtained using a two-sided t-test (P = 0.008).

Source data are available online for this figure.

RMS is still not fully characterized (Hettmer & Wagers, 2010). Therefore, extensive culture optimization of healthy tissue first, with translation to cancerous tissue later, has not been feasible for RMS. Consequently, optimization had to be conducted on tumor samples, which are not readily available. Second, and related to the issue of tissue availability, sarcomas are far less common than carcinomas, accounting for less than 1% of all solid adult malignancies (Burningham *et al*, 2012). In pediatric cancer however, sarcomas are much more common, encompassing 21% of all solid tumors in children (Burningham *et al*, 2012). This further highlights the importance of the currently described approach and collection.

Representation of a broad spectrum of RMS subtypes

The tumoroid models include representatives of both major histological subtypes, all major fusion types, different age groups, both sexes, treatment-naïve as well as pretreated, primary as well as metastatic tumors (Fig 1B, Table 1 and Dataset EV1). Rarer subtypes such as sclerosing/spindle-cell RMS (Rudzinski *et al*, 2015) will be exciting to include, as also indicated by a recent case report (Acanda De La Rocha *et al*, 2021). Compared to patient incidence rates (Glosli *et al*, 2021), the collection has an underrepresentation of head and neck RMS. Although RMS tumor samples from this region were acquired, models from such samples failed, regardless of subtype, clinical stage, sample quantity, or quality. Interestingly, this indicates that RMS arising in the head and neck may depend on specific factors that have not yet been identified. Studies in genetically engineered mice indicate that aberrant Hedgehog signaling can give rise to FN-RMS from nonmyogenic endothelial progenitors in the head and neck (Drummond *et al*, 2018). Here, activation of Hedgehog signaling by Smoothened agonists did not facilitate establishment of head and neck

 © 2022 The Authors

RMS tumoroid models, indicating that additional factors may be necessary. Regardless of such future developments, the protocol described here yields models that can be rapidly established from a broad range of quite different RMS subtypes.

New models for RMS research

Currently available preclinical models of RMS include conventional cell lines, various genetically engineered animal models (GEMs), as well as patient-derived xenograft models (PDX or O-PDX when transplanted orthotopically; Kashi *et al*, 2015; Imle *et al*, 2021). Each of these systems possesses specific benefits and drawbacks, resulting in suitabilities for different research questions or stages in drug development (Kim *et al*, 2020). While conventional cell lines are easy to use, with low costs, large-scale screening potential, and ease of genetic modification, due to prolonged culturing they often do not recapitulate many basic features of the genetic and molecular background of the tumor they were derived from, thus possessing only limited predictive value (Hinson *et al*, 2013). GEMs, on the other hand, are well-suited for cell of origin studies and can provide valuable insights into cancer onset mechanisms. GEMs are usually not suitable for high-throughput screening, mostly due to the low tumor penetrance or the intricacies of animal studies (Kersten *et al*, 2017). In contrast to GEMs, in O-PDX models, tumor samples are transplanted onto immune-deficient animals (usually mice, although zebrafish has recently been reported (Yan *et al*, 2019)). This allows for engraftment, growth, and later propagation of the tumor tissue from animal to animal. Tumors propagated in this manner are thought to be genetically stable over time and to reflect the patient tumor they were derived from, giving them predictive value concerning preclinical drug testing (Gao *et al*, 2015). Disadvantages include the necessary use of animals as hosts and the tumor entity-specific engraftment time which can take up to several months (Pompili *et al*, 2016). Furthermore, there is evidence that not all PDX models are genetically stable (Petljak *et al*, 2019).

The RMS tumoroid models combine several of the above-mentioned benefits. They can be rapidly established and expanded like conventional cell lines, enabling drug screening. Importantly, established drug sensitivities of RMS in general and of RMS subtypes specifically (i.e., inhibitors of MAPK and NOTCH signaling in FN-RMS), are retained in tumoroid models, showing their applicability as models in translational RMS research. In addition, tumoroid models in general are suitable for transplantation into mice (Fumagalli *et al*, 2018; Grassi *et al*, 2019; Dekkers *et al*, 2021), which enables pharmacokinetic studies, further broadening their use in translational research. As with cell lines, molecular editing to mimic certain disease backgrounds is possible using CRISPR/Cas9. Unlike cell lines however, tumoroid models depend on defined media including recombinant growth factors as well as an ECM substitute, resulting in higher costs. RMS tumoroid models molecularly resemble the patient tumor they are derived from, with tumor sample-specific SNV patterns being retained in matching tumoroid models, thereby also recapitulating differences between tumor samples derived from the same patient (e.g., RMS108, RMS109, and RMS110 showing overlap but also differences in SNVs in the tumor samples which are retained in the corresponding tumoroid models). A subset of SNVs present in the tumor are not detected in the established RMS tumoroid which can be due to (i) this clone not being present in the tissue piece that was used for the establishment of the tumoroid, and/or (ii) the respective clone was present in the piece but got lost over the course of culturing and/or (iii) the sequencing depth for the tumoroid model was not sufficient to pick up this mutation (30X coverage in the tumoroid model versus 90X coverage in the tumor, respectively). RMS tumoroid models share this resemblance with the patient tumor they were derived from with O-PDX mouse models, while possessing the above-mentioned advantages of rapid establishment and expansion. Compared to O-PDX mouse models, establishment success is lower in RMS tumoroid models, indicating that niche factors in the host mice are important for facilitating establishment. This may be especially important in particular subtypes such as RMS from the head and neck region. On the other hand, RMS tumoroid models are less intricate in their propagation. Lastly, while GEMs are considered essential for cell of origin studies, recent advances have shown that by genetic editing, such studies can now also be performed in tumoroid models (Custers *et al*, 2021). Lastly, the scRNA-seq-based analysis of two RMS tumoroid models indicates that tumor cell heterogeneity, an important hallmark of plasticity, are maintained *in vitro*. To fully assess to what extent this heterogeneity reflects the heterogeneity in the tumor the models were derived from, an extensive analysis of matching primary RMS tumor samples would be necessary, which will be exciting to explore in future studies.

In conclusion, we established a well-characterized, well-annotated collection of RMS tumoroid models, being the first such collection of tumoroid models derived from purely mesenchymal malignant tumors (i.e., sarcomas) and only the second comprehensive tumoroid model collection derived from pediatric cancer (Calandrini *et al*, 2020). This collection contains all major subtypes of RMS and the models can be used for drug screening as well as molecular editing. An interactive, browser-based companion Shiny app (https://rmsdata.rms-biobank.eu/) that makes all the described data easily accessible, accompanies this paper. The RMS tumoroid models will be a useful complementary system to study the biology of RMS and to improve treatment.

Materials and Methods

Tumor sample acquisition

Tumor samples of RMS were obtained via an established tumor sample acquisition route from patients treated at the Emma Children's Hospital Amsterdam (Amsterdam UMC; RMS006, RMS007, RMS013) or as part of the biobank initiative of the Princess Máxima Center for Pediatric Oncology, Utrecht, the Netherlands (PMC; remaining tumor samples). Ethics approval was granted for the biobanking initiative, and the PMC biobank committee granted approval for this project. All patients and/or their legal representatives signed informed consent to have tumor samples taken for biobank usage. Experiments conformed to the principles set out in the WMA Declaration of Helsinki and the Department of Health and Human Services Belmont Report.

A subset of patients was furthermore enrolled in a local personalized medicine trial (i.e., iTHER study) through which a subset of DNA and RNA specimens from RMS tumors were obtained.

© 2022 The Authors

Tumor sample preparation for establishment of RMS tumoroid models

Solid tumor samples (i.e., needle biopsies or resection specimens) were transferred to collection medium (see below) to retain viability of cells. After pathological examination, suitable samples (i.e., containing tumor cells) were processed in a sterile work environment as follows: the sample was transferred to a sterile dish and covered with a droplet of BM1* culture medium (see below) before being minced to fine pieces using scalpels. If the tumor sample was of sufficient size, a portion of this minced mixture was stored viably (see below). The remainder of the mixture was put on a prewet 70 μM strainer, scrapped with a cell scraper to obtain a single-cell suspension and collected in a tube (A, single-cell fraction, cultured in 2D). Tissue fragments left on the strainer were collected in another tube (B, strainer fraction, cultured in 3D).

A: The single-cell fraction was now spun down (300 *g*, 5 min, 4°C) and the supernatant was removed from the resulting pellet. If the pellet was of grey color (i.e., not containing a high percentage of red blood cells, RBCs), the pellet was resuspended in 1 ml of BM1* medium. If the pellet contained a high percentage of RBCs (i.e., by displaying a red stain), the cell pellet was resuspended in red blood cell lysis buffer (Roche) and incubated at room temperature for 5 min. Thereafter, the reaction was stopped by adding collection medium and the mixture was spun down again (as above). Again, supernatant was removed and now the pellet was resuspended in 1 ml of BM1* medium (without Basement Membrane Extract, BME, see below). In each case, cells were now counted using a TC20 Automated Cell Counter (BioRad) to get a rough estimate on viability and cell numbers. Cells were plated with a sufficient density (at least 10,000 viable cells per 1 cm^2 of surface area), supplemented with 0.3–0.5% cold BME type 2 (see below).

B: The strainer fraction was dissolved in pure cold BME (roughly one-third volume strainer fraction and two-third volume BME) and mixed thoroughly. Droplets of 5–10 μl of this mixture were formed on 24- or 48-well prewarmed cell culture plates and incubated for 5 min at room temperature to allow the BME to solidify. Thereafter, the cell culture plates were incubated upside-down for another 25 min at 37°C to allow the material to "sink" to the top of the droplet. Upon completed solidification, BM1* medium (at room temperature (RT) and without BME) was added to the wells so that droplets were very fully submerged in medium.

In the case of bone marrow aspirates as tumor samples (RMS410 and RMS127), sample tubes were spun down (300 g, 5 min, 4°C, slow break) to separate RBCs and nucleated cells (white clot at the bottom of the tube). In the case of RMS410, the normal hematopoietic system was almost entirely superseded by infiltrating tumor cells so that the clot consisted mainly of tumor cells which could directly be plated (in BM1* with BME), which resulted in rapid outgrowth of the model. In the case of RMS127, the percentage of infiltrating tumor cells was estimated by pathology to be low at circa 5–10%. Therefore, initial cultures (plated in BM1* and BME) from the white clot also contained normal nucleated bone marrow cells which, however, were eventually outcompeted by the rapidly growing tumor cells which overtook the culture.

Tumoroid model culturing and propagation

Growing RMS tumoroid models were inspected regularly and showed adequate growth behavior under conventional cell culture conditions (i.e., 37°C, 5% CO_2). All models were regularly tested negative for mycoplasma contamination. Estimated division times ranged from 24 to 72 h for most models. Models were passaged once or twice per week at a confluency of 70 to 80%. For passaging, old culture medium was aspirated and cells were briefly washed with sterile DPBS (Gibco, cat no. 14190144). Cell detachment was performed using TrypLE Express Enzyme (1×, phenol red, Gibco, cat no. 12605010). Depending on the model and the BME percentage, detachment took between 3 and 10 min (higher BME concentrations resulting in longer detachment time). Cells were collected by flushing the well or flask with collection medium and the resulting mixture was spun down (300 *g*, 5 min, 4°C). Thereafter, the supernatant was removed, and the pellet was resuspended in 1 ml of BM1* and cells were counted using a TC20 Automated Cell Counter (BioRad). Single-cell suspensions were mostly re-seeded at the same density as their parental/previous generation/passage.

Cell culture media

Base medium (BM)
To prepare a 500 ml bottle of BM, Glutamax (5 ml, Gibco, cat no. 35050061), Penicillin/Streptomycin (10,000 U/ml, 5 ml, Gibco, cat no. 15140122), and B27 (without vitamin A, 10 ml, Gibco, cat no. 12587010) were added to a full bottle of advanced DMEM/F12 (500 ml, Gibco, cat no. 12634010). BM was stored at 4°C and was used within 2 months.

Complete culture medium (BM1)*
To prepare the complete culture medium BM1*, 47.5 ml of the above-mentioned base medium (BM) were taken and pipetted into 50-ml tube. Thereafter, the components below were added (no specific order). BM1* was stored at 4°C and was used within 7–10 days (thereafter, the stability of the growth factors may be compromised).

Components:

N2	500 μl	Gibco, cat no. 17502048
N-acetylcysteine (500 mM)	125 μl	Sigma, cat no. A9165
MEM nonessential amino acids	500 μl	Gibco, cat no. 11140035
Sodium pyruvate (100 mM)	500 μl	Gibco, cat no. 11360070
Heparin (5,000 U/ml)	5 μl	Sigma, cat no. H3149-10KU
hEGF (2 μg/ml)	500 μl	Peprotech, cat no. AF-100-15
hFGF-basic (40 μg/ml)	50 μl	Peprotech, cat no. 100-18B
hIGF1 (100 μg/ml)	10 μl	Peprotech, cat no. 100-11
RKi (Y-27632, 100 mM)	5 μl	AbMole Bioscience, cat no. M1817
A83-01 (5 mM)	50 μl	Tocris Bioscience, cat no. 2939

Collection medium
To prepare a 500 ml bottle of collection medium, Glutamax (5 ml, Gibco, cat no. 35050061), Penicillin/Streptomycin (10,000 U/ml, 5 ml, Gibco, cat no. 15140122), and HEPES (1 M, 5 ml, Gibco, cat

 © 2022 The Authors

no. 15630049) were added to a full bottle of advanced DMEM/F12 (500 ml, Gibco, cat no. 12634010). Collection medium was stored at 4°C and was used within 2 months.

Basement membrane extract type 2 (BME)

To facilitate attachment of cells, culture medium was supplemented with 0.1–0.5% BME (Cultrex Reduced Growth Factor Basement Membrane Extract, type 2, Pathclear, R&D Systems, 3533-005-02). We observed batch-to-batch variations of this product, resulting occasionally in suboptimal attachment of cells when the BME concentration was too low. On average, 0.2–0.3% BME supplementation was sufficient for stably established RMS tumoroid models. However, upon encountering suboptimal cell attachment, BME concentrations were raised to 0.5% in established cultures. During the initial establishment process, higher concentrations of BME (0.3–0.5%) showed increased attachment rates of cells.

Freezing and storing procedure

RMS tumor samples as well as established RMS tumoroid cultures were viably frozen as follows: a sufficient number of viable cells (at least 0.5 × 10E6, mostly 1 × 10E6) were diluted in 0.5 ml BM1* medium in a cryo tube. Then, an equal volume of freeze-mixture consisting of 80% fetal calf serum (FCS) and 20% DMSO was added dropwise, resulting in final concentrations of 50% BM1* medium, 40% FCS, and 10% DMSO. Samples were frozen slowly using a freezing container in a −80°C freezer. For long-term storage, frozen vials were transferred to liquid nitrogen.

Thawing procedure

Cryo-preserved RMS tumoroid cell suspensions were quickly defrosted in a water bath at 37°C and then immediately dissolved in washing medium (to at least 5 ml of total volume to dilute the DMSO). Samples were then spun down (300 *g*, 5 min, 4°C), supernatant was removed, and the cell pellet was resuspended in 1 ml of BM1* medium. Thereafter, cells were counted using a TC20 Automated Cell Counter (BioRad). Cell viability was on average 20–50% lower compared to viability at the time of freezing. Cells were then plated at a proper density in BME-supplemented BM1* medium and needed 1–2 weeks to recover before being stable enough for further experiments. Restarting tumoroid cultures from cryo vials was possible for all tumoroid models.

Early tumor validation by RT-qPCR

Early during tumoroid establishment (upon first or second passaging), a portion of cells was set aside for evaluation of marker gene expression. For this, cells were spun down (500 *g*, 5 min, 4°C), the supernatant was removed from the pellet, the pellet was dissolved in Trizol (10 min incubation at RT) and was immediately processed or snap-frozen and stored at −80°C until further processing. Upon processing, first the organic and aqueous phases were separated by addition of 20% chloroform, followed by spinning down (maximum centrifugation speed, 15 min, 4°C). The (upper) aqueous phase was further processed using the Direct-zol RNA MiniPrep Kit (Zymo Research) according to the manufacturer's protocol, including the recommended DNAse I treatment. Quality and quantity of isolated RNA were measured using a NanoDrop OneC (Thermo Fisher Scientific). Synthesis of cDNA from isolated RNA as well as a Universal Human Reference RNA that was used as negative control or for normalization (HREF, Stratagene/Agilent # 740000) was performed using an oligo-dT primed SuperScript III Reverse Transcriptase (Invitrogen)-based reaction according to the manufacturer's protocol. RT-qPCR was performed with the obtained cDNAs testing for expression levels of *G6PD* (forward: 5′-ACGGCAACAGATACAAG AAC-3′, reverse: 5′-CGAAGTGCATCTGGCTCC-3′; product size: 86 bp; Amary *et al*, 2007), *DES* (forward: 5′-CCGTGGTCTCTTAC TTTCCTTT-3′, reverse: 5′-CCCACTTTCTCTCCTTCTCAATC-3′; product size: 119 bp), *MYOG* (forward: 5′-TGCCCAACTGAGATTG TCTTC-3′, reverse: 5′-CTGCTACAGAAGTAGTGGCATC-3′, product size: 81 bp), *MYOD1* (forward: 5′-GTAGCAGGTGTAACCGTAACC-3′, reverse: 5′-CACACCATGCCTCAGAGATAAA-3′, product size: 148 bp), the *PAX-FOXO1* fusion transcript (forward: 5′-CCGACAG CAGCTCTGCCTAC-3′, reverse: 5′-TGAACTTGCTGTGTAGGGACAG-3′, product size: 171 bp for *PAX3-FOXO1* and 159 bp for *PAX7-FOXO1*; Ponce-Castañeda *et al*, 2014) as well as the *PAX3-WWTR1* fusion transcript (forward: 5′-AGCACCAGGCATGGATTT-3′, reverse: 5′-TTCGAGGTCTGTGTCTAGGT-5′, product size: 192 bp). Expression levels of *DES*, *MYOG*, *MYOD1*, and *PAX-FOXO1* were normalized to *G6PD* (housekeeping gene) and referenced to the corresponding expression levels in the HREF using the ΔΔCq method (for the fusion transcript only normalization to *G6PD* as the lack of a fusion expression in HREF did not permit a further reference step).

Immunohistochemistry (IHC) and H&E stainings

To perform IHC, tumoroid models were grown as floating 3D spheres. For this, 1–3 × 10E6 viable cells were put into an ultra-low attachment culture flask (Corning Ultra-Low Attachment 75 cm^2 U-Flask, Corning, cat no. 3814) in BM1* but without BME supplementation. Establishment of spheres of sufficient size took between 7 and 12 days, depending on the growth characteristics of the respective tumoroid model. Spheres were harvested by carefully transferring the sphere-containing medium from the flask to a 15-ml tube and sedimenting on ice for 10 min. Thereafter, the supernatant was removed, and the sphere-containing pellet was resuspended in cold PBS to wash off any remaining medium. The mixture was again sedimented (see above), and PBS was aspirated. Spheres were now fixed using formalin 10% (v/v), (= 4% (w/v) HISTO GRADE, neutralized (pH 7.0 ± 0.2), J.T. Baker, 3933.9020 VWR) for 96 h at 4°C after carefully transferring them to a glass vial. The fixed spheres were then washed twice with PBS and dehydration was performed by adding ethanol solutions with increasing percentages (25% EtOH for 15 min, 50% EtOH for 15 min, 70% EtOH for 15 min—after this step, spheres were stored at 4°C and further processed in batches). Now, spheres were stained with 0.8 g/l Eosin Y dissolved in 96% EtOH (Sigma, E4009) for 30 min and subsequently incubated three times with 100% EtOH for 30 min each. Thereafter, spheres were incubated in n-Butanol (three times 30 min) and melted paraffin (three times) before they were Paraffin-embedded using the HistoCore Arcadia H (Leica Biosystems) following the manufacturer's protocol in a medium size mold. Hardened paraffin blocks were cut into 4 μm slices using a microtome (HM 355S Automatic

Microtome, Thermo Scientific). Slides were further processed by deparaffinization (incubation with xylene three times for 3 min, then 100% EtOH two times for 3 min, 95% EtOH two times for 1 min, 70% EtOH once for 1 min, and 50% EtOH once for 1 min) and rehydration (tap water). Thereafter, antigen retrieval was performed by boiling samples for 20 min in citrate buffer (pH 6, for Desmin staining) or Tris-EDTA-Tween buffer (pH 9, for Myogenin and MYOD1). After washing (in TBS-0.025% Triton, twice 5 min) and blocking (1.5 h in TBS-1%BSA), slides were incubated with primary antibody (α-Desmin 1:400, Abcam ab15200 rabbit antibody; α-Myogenin 1:400, Santa Cruz 5FD mouse antibody; α-MYOD1 1:200, Cell Marque EP212 rabbit antibody) overnight at 4°C. The next day, slides were washed twice with PBS and incubated with secondary antibodies (Desmin and MYOD1: anti-rabbit-HRP, BioRad 170-6515; Myogenin: anti-mouse HRP, BioRad 170-6516) 1:500 in PBS-1% BSA. Stainings were visualized using Liquid DAB$^+$ 2-component system (3,3′-diaminobenzidine, DAKO, Agilent K3467) following the manufacturer's protocol and washed three times with TBS. Counterstaining was performed by incubation with thionine (0.05% for 20 min). After subsequent incubation with 96% EtOH, 100% EtOH and xylene, slides were mounted using Permount mounting medium (Fisher Scientific SP15-100) and visualized using a Leica DMi6 microscope.

H&E stainings were performed manually (steps: xylene three times for 5 min, 100% EtOH twice for 1 min, 95% EtOH twice for 30 s, 70% EtOH for 30 s, washed in demi-water, incubation with hematoxylin (Hematox 7211) for 2:45 min, washed with demi-water, brief incubations with acidic EtOH, washed with demi-water, 95% EtOH for 30 s, eosin incubation for 2:45 min, EtOH 70% for 30 s, EtOH 95% for 30 s, EtOH 100% twice for 30 s, xylene three times for 1 min) or were performed at the in-house pathology department following standard protocols. Visualization was performed as described above.

The quality of stainings was evaluated by an in-house pathologist. Representative images of stained spheres are shown and were compared to H&E as well as IHC stainings obtained in pathology for the RMS tumor sample the tumoroid was derived from (representative images chosen by the pathologist).

Single-cell mRNA sequencing of RMS tumoroid models

Sample processing

For each tumoroid model (RMS127 and RMS444), viably frozen cells were thawed and resuspended in BM1* medium. Prior to sorting, 4′,6-diamidino-2-phenylindole (DAPI, Sigma-Aldrich, #D9542) and DRAQ5 (Thermo Fisher, #65-0880-92) were added to the single-cell suspensions to achieve final concentrations of 1 and 5 μM, respectively. Viable single-cells (DAPI$^-$, DRAQ5$^+$, FSC/SSC) were then sorted into 384-well plates (BioRad, #HSP3801) containing 10 μl of mineral oil (Sigma, #M5310) and 50 nl of barcoded RT primers using a SONY SH800S Cell Sorter. Libraries were prepared according to the SORT-seq protocol (Muraro *et al*, 2016) and sequenced on the Illumina NextSeq500 (paired-end, 75 bp).

Analysis of single-cell mRNA-sequencing data

Sequencing reads were demultiplexed, mapped to the Genome Reference Consortium human build 38 genome, and transcript counts were generated using a custom implementation of the zUMIs pipeline (Parekh *et al*, 2018). Count tables from each plate were read into *R* (v4.1.0), merged and metadata fields were compiled. The merged count and metadata tables were used to initialize a Seurat (v4.0.3) object (Hao *et al*, 2021), and cells with < 500 expressed genes, < 800 or > 50,000 unique transcripts, a percentage of mitochondrial transcripts > 50%, a percentage of hemoglobin genes > 1% or a ratio of intergenic to genic transcripts > 2 were excluded from further analysis. The filtered object was then log-normalized, using a scaling factor of 10,000, and the top 2,000 most variable genes in the dataset were defined by calling the FindVariableFeatures Seurat function (default parameters). These genes were scaled, centered and used as input for running principal component analysis (PCA). The top five principal components were then used to project the data in two-dimensions using *t*-distributed stochastic neighbor embedding (t-SNE). Single-cell CNV profiles were constructed using the InferCNV *R* package (v1.8.0; Tickle *et al*, 2019). In addition to the default parameters, an average expression threshold per gene of 0.3 and a standard deviation filter of 2 was used to denoise the results. A dataset of cord blood mononuclear cells (CBMCs) generated in-house was used as the "normal" reference sample.

RNA and DNA isolation from tumor and derived tumoroid samples for whole-genome sequencing (WGS) and bulk mRNA sequencing (RNA-seq)

Tumoroid models

To isolate RNA and DNA for WGS and RNA-seq, tumoroid cells were collected as a pellet, snap-frozen, and stored at −80°C. Isolation of RNA and DNA from the same pellet was performed using the AllPrep DNA/RNA/miRNA Universal Kit (Qiagen # 80224) according to the manufacturer's protocol. Lysis in RLT buffer was followed by homogenization using a Qiashredder column as described. The flow-through fraction in RLT buffer was used for the Allprep DNA and RNA isolations. Quality and quantity of isolated RNA and DNA were measured by using the NanoDrop OneC (Thermo Fisher Scientific), Bioanalyzer 2100 (Agilent), Qubit Fluorometer (Thermo Fisher Scientific), and marker-checks using RT-qPCR (as above).

Tumor samples and germline control samples (EDTA blood)

Resected tissue and/or biopsies were processed within 10 min after removal from the patient. DNA and RNA were isolated from the same piece of fresh frozen tissue using the AllPrep DNA/RNA/miRNA Universal Kit (Qiagen # 80224) using the QIAcube Connect (Qiagen). Reference DNA was isolated from peripheral white blood cells (EDTA blood) using the same method.

Whole-genome sequencing (WGS) of tumor and tumoroid samples

Sequencing

One hundred and fifty nanograms of total DNA were used for library preparation using the KAPA HyperPlus kit (Roche), according to manufacturer's instructions. Libraries from tumor and normal tissue were pooled in a 3:1 ratio, with a total of 7 tumor/normal pairs per S4 sequencing kit. Libraries were sequenced using 2 × 150 cycles on a NovaSeq 6000 (Illumina).

 © 2022 The Authors

Preprocessing, alignment, and annotation

The WGS sequencing data were processed as per the GATK 4.0 best practices workflow for variant calling, using a wdl and cromwell-based workflow. Reads were aligned to GRCh38 using bwamem (v0.7.13), and quality control (QC) was performed using FastQC (v0.11.5) and picardTools (v2.20.1). Somatic variants were identified using Mutect2 from GATK v4.1 and annotated using Vep (v92). Likewise, copy-number alterations (CNAs) were identified using GATK v4.1.

Identification of nonsynonymous single-nucleotide variants (SNVs)

Raw VCF files from germline, tumor, and tumoroid samples were processed with vcftools (v0.1.13; Danecek *et al*, 2011). Only variants that met the criteria (passed all quality filters in addition to PHRED quality score 100 (250 for indels) and minimum read depth of 10) were kept for further analyses. Indels were also filtered out if the minimum mapping quality (MQ) was below 60. Bcftools (https://github.com/samtools/bcftools) was used to remove all common snps (VAF ≥ 1%) present in dbSNP (https://www.ncbi.nlm.nih.gov/snp/, v151 downloaded in August 2020). Filtered files were then loaded into *R* (v4.0.2) and processed with the package VariantAnnotation (Obenchain *et al*, 2014) and packages from the tidyverse. Variants in tumor and tumoroid models also present in the germline sample were removed from further analyses; remaining variants were filtered for VAFs > 0.3 and those tagged as missense variant, stop gained, stop lost, start lost, in-frame insertion, in-frame deletion, and frameshift variant were selected as nonsynonymous somatic mutations. Figures were generated with ggplot2 (v.3.3.2).

Signature analysis

Somatic signature analysis was performed using the *R* programming language (v3.6.3) and the *R* packages MutationalPatterns (v3.2.0; Manders *et al*, 2022) and VariantAnnotation (v1.32.0; Obenchain *et al*, 2014; Blokzijl *et al*, 2018). Somatic variants were filtered on both the variant allele fraction (VAF) and depth (DP). For both the control and the tumor/tumoroid samples we used DP ≥ 20. For the tumor/tumoroid samples we used VAF > 0.3, while in control samples the VAF had to be 0. Samples with 50 or less mutations would have been excluded, but this was not the case for any samples in our cohort. A mutation matrix was generated that shows how often each of the 96 types of base substitutions occurred in each sample. A variational Bayesian non-negative matrix factorization (NMF) was performed on this matrix to extract four *de novo* mutational signatures for base substitutions. The cosine similarities were then calculated between these *de novo* signatures and a set of signatures consisting of both the COSMIC signatures (v3.2, GRCh38) and the SIGNAL exposure signatures (Kucab *et al*, 2019; Alexandrov *et al*, 2020). The four signatures most similar to the *de novo* signatures (SBS1, SBS5, SBS18, and temozolomide [TMZ]) were then used for signature refitting. Signature refitting was performed using the *fit_to_signatures_strict* function using the *best_subset* method with a *max_delta* of 0.004. Our approach of first performing *de novo* signature extraction followed by refitting is similar to the approach suggested by Maura *et al* (2019).

To determine the similarity between the samples, the cosine similarities of their base substitution profiles were calculated. This resulted in a cosine similarity matrix that was used to calculate the distance between samples, which was then used for hierarchical clustering.

Evaluation of clonal heterogeneity using WGS data

To evaluate clonal heterogeneity in our RMS tumoroid models, two approaches were used based on the WGS data from each tumor/tumoroid pair. First, we compared the VAFs of somatic mutations in both tumor and tumoroid models by selecting variants from coding regions with a VAF of at least 0.1 in either tumor or tumoroid model. To ensure the VAFs were representative of the ratio of cell populations in the culture, we removed all SNVs from noncopy number neutral regions or tagged as sites with loss of heterozygosity (LOH) as detected by the GATK pipeline. VAFs of SNVs matching those criteria were plotted a in correlogram using *R*.

Second, we investigated the alternate allele (B-allele) fractions as they can indicate the presence of clonal and/or subclonal populations based on estimated allelic ratios. To this end, we extracted the median alternate allele fraction from the modeled segments from each sample; to ensure comparability across region segments, a consensus region .bed file was generated prior to generating the density plots. Frequencies were then plotted per tumor/tumoroid pair as density plots using *R*.

For both analyses, RMS127 was excluded due the low tumor cell percentage of the tumor sample.

Bulk mRNA (RNA-seq) of tumor and tumoroid samples: transcriptional profile and gene fusions

Sequencing

Three hundred nanograms of total RNA were used for library preparation using the KAPA RNA HyperPrep kit with RiboErase (Roche), according to manufacturer's instructions. The protocol was optimized to achieve an insert size of ~300–400 nt.

RNA libraries were pooled with a total of 25 samples per S1 kit or 60 samples per S2 kit. Libraries were sequenced using 2 × 150 cycles on a NovaSeq 6000 (Illumina).

Preprocessing: alignment, annotation, and detection of gene fusions

The RNA sequencing data were processed as per the GATK 4.0 best practices workflow for variant calling, using a wdl and Cromwell-based workflow (https://gatk.broadinstitute.org/hc/en-us/sections/360007226651-Best-Practices-Workflows). This included performing quality control with Fastqc (v0.11.5) to calculate the number of sequencing reads and the insert size (Andrews S., 2010. FastQC: a quality control tool for high throughput sequence data, http://www.bioinformatics.babraham.ac.uk/projects/fastqc). Picard (v2.20.1) for RNA metrics output and MarkDuplicates ("Picard Tools." Broad Institute. http://broadinstitute.github.io/picard/). The raw sequencing reads were aligned using Star (v2.7.0f) to GRCh38 and gencode version 31. Gene fusion detection was performed using Star fusion (v1.6.0; Haas *et al*, 2019). Finally, expression counts were determined at exon and gene level using Subread Counts (Liao *et al*, 2019).

Processing of transcriptome data

Raw count tables were loaded into *R* (v4.0.2) and processed with the packages from the tidyverse. Count matrices were transformed

© 2022 The Authors

into log2-scale transcripts per million (log2 TPM) tables and genes annotated with the same gene symbol merged.

Comparison of transcriptional profiles across samples (kidney and rhabdomyosarcomas) was performed via correlation performed in *R* using the base package.

Growth curve experiments

Growth behavior in 3D sphere cultures prior to drug screenings (see below) was tested via performing growth curve experiments. Tumoroid cells were plated at different densities (typically between 250 and 4,000 viable cells per well) in 384-well round bottom ultra-low attachment spheroid microplates (Corning, cat no. 3830). To facilitate 3D sphere formation, cells were grown in BM1* medium without BME and after dispensing them into the plates, plates were spun (350 *g*, 5 min, slow break) to concentrate cells in the center of the wells. Plates were incubated at standard conditions and cells were granted a recovery period of 48 h. Thereafter, readouts were performed using CellTiter-Glo 3D Cell Viability Assay (CTG3D, Promega, cat no. G9683) according to the manufacturer's protocol at three time points: immediately (T0), 48 or 72 h (T48 or T72), and 120 h (T120). Measured luminescence (via FLUOstar Omega, BMG Labtech) was averaged per readout per cell density and the corresponding background signal from medium was subtracted. Obtained measurements from T48/T72 to T120 were normalized to T0 to calculate the relative growth over the period of 120 h. For the following drug screening experiments (see below), a cell density was chosen that showed logarithmic growth behavior in growth curve experiments. Defined starting number of cells facilitated the establishment of a sphere and were optimized for intrinsic growth factor levels and space depletion in the well over the course of the experiment. Growth curve experiments were performed once per model but with at least ten technical replicates per number of cells plated.

Drug screenings

For drug screenings, tumoroid models were processed according to the same protocol as for the growth curve experiments, with a number of cells seeded that was determined in those experiments (see above). Drugs, dissolved in DMSA or water, were added 48 h after seeding of the tumoroid spheres fully automated via a robotics system: Up until 2019, this was facilitated via a Caliper Sciclone-Robotic Liquid Handler using a dilution of the drugs in medium and transferring this dilution to the cells by pipetting. From 2020 onward, screenings were performed at the high-throughput screening (HTS) facility of the Princess Máxima Center with a Biomek i7 liquid handler, using the acoustic liquid handler Echo550 for direct drug transfers. Before the screening, the 384-well working plates containing the dissolved drugs are shaken (30 min, RT) and centrifuged (1 min, 350 *g*). Tumoroid spheres were treated with a tenfold dilution series of the drugs on the library plate (0.1 nM to 10 μM). Positive control samples were treated with DMSO, negative control samples with staurosporine (final concentration of 10 μM). Readouts were performed using CellTiter-Glo 3D Cell Viability Assay (CTG3D, Promega, cat no. G9683) according to the manufacturer's protocol at T0 (before addition of drugs–control) and at T120 (120 h after addition of drugs–readout). Dose-response was estimated per drug and concentration in relation to the DMSO-treated cells (set to 100%) and empty controls (set to 0%). Quality of the screenings was approved after assessment of the cell growth (absorbance signal of T120 over T0), the negative, positive, and empty controls and, if applicable, the amount of variability between the duplicates.

Depending on the available number of cells at drug screening, a subset of models (RMS007, RMS109, RMS110, RMS000EEC, RMS000ETY, RMS000FLV, RMS000HQC, RMS000HWO, RMS000HWQ) could be screened in technical duplicates while for the other models (RMS006, RMS012, RMS013, RMS102, RMS108, RMS127, RMS335, RMS410, RMS444, RMS000CPU) only screening without technical duplicates was feasible. To test reproducibility, we analyzed the correlation between the technical duplicates in the tumoroid models for which those were available (Appendix Fig S1). As we observed a very high correlation there ($R = 0.91$), indicating a high reproducibility, we deemed it appropriate to combine the analysis of samples tested in duplicate and those not tested in duplicate given the scope of the assay was to assess whether we could obtain biologically meaningful results (any "hit" should have been further validated). RMS tumoroid models RMS000ETY and RMS000HWO were excluded from the analysis as they did not show an increase of CTG3D signal between T0 and T120 (Table EV1), indicating that these two models did not grow sufficiently under the screening conditions (while they displayed a minor increase in signal during the growth curve experiments).

Further data analysis was performed using *R* (v3.6.3). Area Under the Curve (AUC) values were calculated for every drug per tumoroid model and replicate (in the case of the tumoroid models with technical replicates) using the *auc* function of the MESS package (v0.5.6). In the case of RMS tumoroid models with technical duplicates, the two resulting AUC values per drug were correlated (using the *cor* function of base *R*) to calculate the correlation between replicates and plotted using the *plot* function of base *R* (see above). For the further downstream analysis, these replicate AUC values were averaged, resulting in a matrix with one AUC value per drug per RMS tumoroid model. On this matrix, unsupervised clustering was performed using the *get_dist* function from the factoextra package (v1.0.7) using the arguments "pearson" for RMS tumoroid models and "euclidean" for drugs to measure dissimilarity with further clustering using the *hclust* function from base *R* using the "average" argument for linkage. Data were visualized using the *heatmap.2* function from the gplots package (v3.0.3) using dendrograms generated from the established clustering. Principal component analysis (PCA) was performed using the *prcomp* function from base *R* with exclusion of the RMS tumoroid model RMS000FLV due to its outlier behavior. PCAs were visualized using the *fviz_pca_ind* and *fviz_pca_var* functions from the above-mentioned factoextra package.

CRISPR/Cas9 knockout of *TP53* and functional evaluation

RMS012 tumoroid cells were kept under standard conditions (BM1* supplemented with 0.1% BME) prior to the experiment. Upon reaching 70% confluency, tumoroid cells were passaged as usual and seeded with high density into wells of a 24-well plate. After 24 h of recovery, transfection was performed: Nucleic acid–Lipofectamine 2000 complexes were prepared according to the standard Lipofectamine 2000 protocol (Invitrogen). Four μl of Lipofectamine 2000

 © 2022 The Authors

reagent in 50 μl Opti-MEM medium (Gibco) and 1.5 μg of DNA (pSpCas9(BB)-2A-GFP control or sgRNA *TP53* plasmid in 50 μl Opti-MEM medium) were mixed, incubated for 5 min, and added to the cells. Plasmids were kindly shared by Jarno Drost (PMC, NL). For the plasmid sequence refer to Drost *et al* (2015). Twenty-four hours after transfection, transfection efficiency was evaluated using fluorescence microscopy, detecting GFP positive cells. Forty-eight hours after transfection, selection with nutlin-3 (10 μM) was started. Four days after the start of selection, first nutlin-3 resistant colonies could be detected in *TP53* knockout plasmid transfected cells whereas cells transfected with control plasmids died due to nutlin-3 exposure. Putative knockout cells were further expanded.

Knockout was confirmed using Western Blotting and Sanger sequencing of genomic DNA: For Western Blotting, snap-frozen tumoroid cell pellets were lysed in Phosphatase-substituted RIPA buffer and run on a 10% precast gel for P53 detection (BioRad). Protein levels of P53 (1:1,000, Santa Cruz DO-1 P53 antibody) were detected while Histone 3 (1:2,000, Abcam ab1791 Pan-H3) served as loading control. For visualization, secondary antibodies (goat anti-rabbit, BioRad 1706515/goat anti-mouse BioRad 1706516) conjugated with horseradish peroxidase (HRP) were used together with ECL substrate (Perkin Elmer) on an imaginer (BioRad ChemiDoc). Western Blotting analysis was performed twice, and a representative blot is shown.

For Sanger sequencing of genomic DNA, a snap-frozen tumoroid cell pellet was lysed using DirectPCR Lysis Reagent for Cells (Viagen) according to the manufacturer's protocol. PCR amplification was performed using Phusion High-Fidelity DNA Polymerase (New England BioLabs) using the following primers: forward 5′-CCCATC TACAGTCCCCCTTG-3′, reverse 5′-CAGGAAGCCAAAGGGTGAAGA-3′. PCR products were cleaned up and concentrated using a DNA Clean-up and Concentration Kit (Zymo Research) according to the manufacturer's protocol. Gel electrophoresis indicated the presence of a specific product which was sent for Sanger sequencing using the following primers: forward 5′-TGGTTCACTGAAGACCCAGG-3′, reverse 5′-GAAGTCTCATGGAAGCCAGCC-3′. Obtained sequences were aligned and inspected using the Benchling browser tool (https://www.benchling.com/). Furthermore, sequencing data was submitted for TIDE (Tracking of Indels by Decomposition, http://tide.nki.nl) analysis to infer the composition of Indels in the knockout population (Brinkman *et al*, 2014).

For detection of DNA double strand breaks, induction of γH2AX was measured using Western Blotting. Fusion-negative embryonal rhabdomyosarcoma cell line RD (kindly shared by Jan Molenaar, PMC, NL) served as a control (either untreated or treated with 1, 5, or 10 μM of staurosporine (Sigma) for 24 h). RD cells (ATCC identifier CCL-136, species human) were cultured under conventional conditions as detailed by the American Type Culture Collection (ATCC) with regular testing for mycoplasma contamination. For Western Blotting, snap-frozen tumoroid (RMS012 *TP53* KO) or tumor (RD) cell pellets were lysed in Phosphatase-substituted RIPA buffer and run on a 15% self-cast gel for γH2AX (p.S139) detection. Protein levels of γH2AX (p.S139, 1:2,000, Abcam ab26350 antibody) were detected while GAPDH (1:1,000, Abcam ab9485 antibody) served as loading control. Visualization was performed as described above for the confirmation of the knockout. As above, Western Blotting analysis was performed twice, and a representative blot is shown.

The paper explained

Problem

Rhabdomyosarcomas (RMS) are the most commonly diagnosed soft tissue sarcomas in children and adolescents. Despite advances in treatment, prognosis for high-risk RMS patients remains poor, in part due to preclinical models that insufficiently recapitulate the disease.

Results

We describe the first collection of well-characterized RMS tumor organoid (tumoroid) preclinical models, which constitutes the first collection of tumoroids derived from purely malignant mesenchymal tumors (i.e., sarcomas) and only the second tumoroid model collection of pediatric cancers. Established models cover all major subtypes and clinical presentations of RMS, and faithfully recapitulate the molecular biology of the primary tumor sample they were derived from. Of note, RMS tumoroid models not only retain key driver mutations but also transcriptional and clonal heterogeneity in vitro. RMS tumoroid models can be rapidly established and expanded to perform drug screening as fast as 27 days after sample acquisition (median 81 days), indicating their potential for precision medicine approaches to inform treatment decisions. Lastly, RMS tumoroid models are amenable to CRISPR/Cas9-based editing to generate biologically relevant mutants like P53-deficient fusion-negative RMS (FN-RMS), recently shown to have worse prognosis than P53 proficient FN-RMS.

Impact

RMS tumoroid models will be valuable additions to the repertoire of preclinical models of RMS to ultimately improve treatment and prognosis of patients suffering from this highly aggressive disease.

To assess differential response to prexasertib (MedchemExpress), RMS012 tumoroid models (*TP53* wildtype and *TP53* knockout) were processed according to the same protocol as for the growth curve experiments (see above), with 500 cells seeded per well. Prexasertib (dilution series from 200 to 0.78125 nM) was added 48 h after seeding of the tumoroid spheres. Control samples were treated with DMSO. Readouts were performed using CellTiter-Glo 3D Cell Viability Assay (CTG3D, Promega, cat no. G9683) according to the manufacturer's protocol at T72 (72 h after addition of drugs). Dose-response was estimated per model and concentration in relation to the DMSO-treated cells (set to 100%). The experiment was performed three times. Further data analysis was performed using *R* (v3.6.3). A sigmoidal fit for the dose-response curve was calculated per replicate for both the knockout and wild-type sample with a three-parameter log-logistics function using the drc package (v3.0-1; Ritz *et al*, 2015). The statistical significance of the differences in fitted IC_{50} values between knockout and wildtype were obtained using a two-sided *t*-test. For visualization purposes, a three-parameter sigmoidal fit per model (not per replicate) was used.

Image postprocessing and figure preparation

Microscopy images from RMS tumoroid IHC and H&E stainings as well as images from Western Blotting were postprocessed according to good scientific practice with Adobe Photoshop 2021 and Fiji (v2.0.0-rc-69/1.52i; Schindelin *et al*, 2012). Images from original RMS tumors (H&E and IHC) were not processed. Figures were prepared using Adobe Illustrator 2021.

© 2022 The Authors

Data availability

Bulk sequencing data (i.e., whole-genome sequencing and bulk mRNA sequencing) have been made openly available at the European Genome-Phenome Archive (EGA) with the identifiers EGAD 00001008466 (https://ega-archive.org/datasets/EGAD00001008466; “WGS soft tissue sarcoma tumoroid biobank”), EGAD00001008467 (https://ega-archive.org/datasets/EGAD00001008467; “RNA-Seq soft tissue sarcoma tumoroid biobank”), and EGAD00001008709 (https://ega-archive.org/datasets/EGAD00001008709; “RNA-Seq of primary pediatric kidney tumor controls for the soft tissue sarcoma tumoroid biobank”). Single-cell mRNA sequencing data of the RMS tumoroid models RMS127 and RMS444 as well as the CBMC controls are similarly available at EGA with the identifier EGAD00001009002 (https://ega-archive.org/datasets/EGAD00001009002; “sc-RNA-Seq soft tissue sarcoma tumoroid biobank”). Code for bulk sequencing data analysis is made openly available at https://github.com/teresouza/rms2018-009.

Expanded View for this article is available online.

Acknowledgements
We thank the patients and their parents for contributing by consenting to this study; the nurses, doctors, and other health care professionals in our institute with whom we work closely; our center’s high-throughput screening facility, in particular Bianca Koopmans, Kimberley Ober, and Sander van Hooff; Philip Lijnzaad, Thanasis Margaritis, and Tito Candelli from our center’s single-cell genomics facility, as well as past and present members of the Holstege group. MTM received financial support from the Deutsche Forschungsgemeinschaft (#408083583). CC was supported by Children Cancer-free Foundation (Stichting Kinderen Kankervrij; #292). JDr received support from the European Research Council (ERC) starting grant #850571 and the Dutch Cancer Society (KWF)/Alpe d’HuZes Bas Mulder award (#10218). We are grateful for the financial support provided by the Foundation Children Cancer-free (Stichting Kinderen Kankervrij core funding).

Author contributions
Michael T Meister: Conceptualization; supervision; investigation; visualization; methodology; writing – original draft; writing – review and editing. **Marian JA Groot Koerkamp:** Data curation; investigation; methodology. **Terezinha de Souza:** Data curation; investigation; visualization. **Willemijn B Breunis:** Investigation; methodology. **Ewa Frazer-Mendelewska:** Investigation. **Mariël Brok:** Investigation. **Jeff DeMartino:** Investigation; visualization. **Freek Manders:** Investigation; visualization. **Camilla Calandrini:** Investigation. **Hinri HD Kerstens:** Data curation. **Alex Janse:** Data curation. **M Emmy M Dolman:** Resources. **Selma Eising:** Resources. **Karin PS Langenberg:** Resources. **Marc van Tuil:** Investigation. **Rutger RG Knops:** Resources. **Sheila Terwisscha van Scheltinga:** Resources. **Laura S Hiemcke-Jiwa:** Resources. **Uta Flucke:** Resources. **Johannes HM Merks:** Resources. **Max M van Noesel:** Resources. **Bastiaan BJ Tops:** Resources. **Jayne Y Hehir-Kwa:** Resources. **Patrick Kemmeren:** Resources. **Jan J Molenaar:** Resources. **Marc van de Wetering:** Resources. **Ruben van Boxtel:** Resources. **Jarno Drost:** Resources; writing – review and editing. **Frank CP Holstege:** Conceptualization; supervision; funding acquisition; writing – original draft.

In addition to the CRediT author contributions listed above, the contributions in detail are:

Conceptualization: MTM and FH; Methodology: MTM, MJAGK and WBB. Investigation: MTM, MJAGK, TS, WBB, EF-M, MB, JDe, CC, FM, and MT; Resources: MEMD, SE, KPSL, RRGK, STS, LSH-J, UF, JHMM, MMN, BBJT, JYH-K, PK, JJM, MW, RB and JDr; Data curation: MJAGK, TS, AJ and HHDK; Writing—Original Draft: MTM and FCPH; Writing—Review and Editing: MTM and JDr; Visualization: MTM, TS, JDe, and FM; Funding Acquisition: FCPH; Supervision: MTM and FCPH.

Disclosure and competing interests statement
The authors declare that they have no conflict of interest.

References

Abdullah KG, Bird CE, Buehler JD, Gattie LC, Savani MR, Sternisha AC, Xiao Y, Levitt MM, Hicks WH, Li W *et al* (2021) Establishment of patient-derived organoid models of lower grade glioma. *Neuro Oncol* 24: 612 – 623

Acanda De La Rocha AM, Fader M, Coats ER, Espinal PS, Berrios V, Saghira C, Sotto I, Shakya R, Janvier M, Khatib Z *et al* (2021) Clinical utility of functional precision medicine in the management of recurrent/relapsed childhood rhabdomyosarcoma. *JCO Precis Oncol* 5: 1659 – 1665

Alexandrov LB, Kim J, Haradhvala NJ, Huang MN, Tian Ng AW, Wu Y, Boot A, Covington KR, Gordenin DA, Bergstrom EN *et al* (2020) The repertoire of mutational signatures in human cancer. *Nature* 578: 94 – 101

Amary MFC, Berisha F, Bernardi FDC, Herbert A, James M, Reis-Filho JS, Fisher C, Nicholson AG, Tirabosco R, Diss TC *et al* (2007) Detection of SS18-SSX fusion transcripts in formalin-fixed paraffin-embedded neoplasms: analysis of conventional RT-PCR, qRT-PCR and dual color FISH as diagnostic tools for synovial sarcoma. *Mod Pathol* 20: 482 – 496

Belyea BC, Naini S, Bentley RC, Linardic CM (2011) Inhibition of the notch-hey1 axis blocks embryonal rhabdomyosarcoma tumorigenesis. *Clin Cancer Res* 17: 7324 – 7336

Bersani F, Taulli R, Accornero P, Morotti A, Miretti S, Crepaldi T, Ponzetto C (2008) Bortezomib-mediated proteasome inhibition as a potential strategy for the treatment of rhabdomyosarcoma. *Eur J Cancer* 44: 876 – 884

Bisogno G, De Salvo GL, Bergeron C, Gallego Melcón S, Merks JH, Kelsey A, Martelli H, Minard-Colin V, Orbach D, Glosli H *et al* (2019) Vinorelbine and continuous low-dose cyclophosphamide as maintenance chemotherapy in patients with high-risk rhabdomyosarcoma (RMS 2005): a multicentre, open-label, randomised, phase 3 trial. *Lancet Oncol* 20: 1566 – 1575

Bleijs M, Wetering M, Clevers H, Drost J (2019) Xenograft and organoid model systems in cancer research. *EMBO J* 38: e101654

Blokzijl F, Janssen R, van Boxtel R, Cuppen E (2018) MutationalPatterns: Comprehensive genome-wide analysis of mutational processes. *Genome Med* 10: 33

Boulay G, Cironi L, Garcia SP, Rengarajan S, Xing YH, Lee L, Awad ME, Naigles B, Iyer S, Broye LC *et al* (2021) The chromatin landscape of primary synovial sarcoma organoids is linked to specific epigenetic mechanisms and dependencies. *Life Sci Alliance* 4: e202000808

Brinkman EK, Chen T, Amendola M, Van Steensel B (2014) Easy quantitative assessment of genome editing by sequence trace decomposition. *Nucleic Acids Res* 42: e168

Brodin BA, Wennerberg K, Lidbrink E, Brosjö O, Potdar S, Wilson JN, Ma L, Moens LN, Hesla A, Porovic E *et al* (2019) Drug sensitivity testing on patient-derived sarcoma cells predicts patient response to treatment and identifies c-Sarc inhibitors as active drugs for translocation sarcomas. *Br J Cancer* 120: 435 – 443

Burningham Z, Hashibe M, Spector L, Schiffman JD (2012) The epidemiology of sarcoma. *Clin Sarcoma Res* 2: 14

Calandrini C, Schutgens F, Oka R, Margaritis T, Candelli T, Mathijsen L, Ammerlaan C, van Ineveld RL, Derakhshan S, de Haan S *et al* (2020) An

 © 2022 The Authors

organoid biobank for childhood kidney cancers that captures disease and tissue heterogeneity. *Nat Commun* 11: 1310

Casey DL, Wexler LH, Pitter KL, Samstein RM, Slotkin EK, Wolden SL (2020) Genomic determinants of clinical outcomes in rhabdomyosarcoma. *Clin Cancer Res* 26: 1135 – 1140

Clevers H (2016) Modeling development and disease with organoids. *Cell* 165: 1586 – 1597

Custers L, Khabirova E, Coorens THH, Oliver TRW, Calandrini C, Young MD, Vieira Braga FA, Ellis P, Mamanova L, Segers H *et al* (2021) Somatic mutations and single-cell transcriptomes reveal the root of malignant rhabdoid tumours. *Nat Commun* 12: 1 – 11

Danecek P, Auton A, Abecasis G, Albers CA, Banks E, DePristo MA, Handsaker RE, Lunter G, Marth GT, Sherry ST *et al* (2011) The variant call format and VCFtools. *Bioinformatics* 27: 2156 – 2158

Defachelles A-S, Bogart E, Casanova M, Merks JHM, Bisogno G, Calareso G, Gallego Melcon S, Gatz SA, Le Deley M-C, McHugh K *et al* (2021) Randomized phase II trial of vincristine-irinotecan with or without temozolomide, in children and adults with relapsed or refractory rhabdomyosarcoma: a European Paediatric Soft Tissue Sarcoma Study Group and Innovative Therapies for Children With Cancer. *J Clin Oncol* 39: 2979 – 2990

Dekkers JF, van Vliet EJ, Sachs N, Rosenbluth JM, Kopper O, Rebel HG, Wehrens EJ, Piani C, Visvader JE, Verissimo CS *et al* (2021) Long-term culture, genetic manipulation and xenotransplantation of human normal and breast cancer organoids. *Nat Protoc* 16: 1936 – 1965

Dias P, Chen B, Dilday B, Palmer H, Hosoi H, Singh S, Wu C, Li X, Thompson J, Parham D *et al* (2000) Strong immunostaining for myogenin in rhabdomyosarcoma is significantly associated with tumors of the alveolar subclass. *Am J Pathol* 156: 399 – 408

Dijkstra KK, Monkhorst K, Schipper LJ, Hartemink KJ, Smit EF, Kaing S, de Groot R, Wolkers MC, Clevers H, Cuppen E *et al* (2020) Challenges in establishing pure lung cancer organoids limit their utility for personalized medicine. *Cell Rep* 31: 107588

Dobbelstein M, Sørensen CS (2015) Exploiting replicative stress to treat cancer. *Nat Rev Drug Discov* 14: 405 – 423

Drost J, Van Jaarsveld RH, Ponsioen B, Zimberlin C, Van Boxtel R, Buijs A, Sachs N, Overmeer RM, Offerhaus GJ, Begthel H *et al* (2015) Sequential cancer mutations in cultured human intestinal stem cells. *Nature* 521: 43 – 47

Drummond CJ, Hanna JA, Garcia MR, Devine DJ, Heyrana AJ, Finkelstein D, Rehg JE, Hatley ME (2018) Hedgehog pathway drives fusion-negative rhabdomyosarcoma initiated from non-myogenic endothelial progenitors. *Cancer Cell* 33: 108 – 124

Enterline HT, Horn RC (1958) Alveolar rhabdomyosarcoma; a distinctive tumor type. *Am J Clin Pathol* 29: 356 – 366

Fumagalli A, Suijkerbuijk SJE, Begthel H, Beerling E, Oost KC, Snippert HJ, Van Rheenen J, Drost J (2018) A surgical orthotopic organoid transplantation approach in mice to visualize and study colorectal cancer progression. *Nat Protoc* 13: 235 – 247

Fusco P, Parisatto B, Rampazzo E, Persano L, Frasson C, Di Meglio A, Leslz A, Santoro L, Cafferata B, Zin A *et al* (2019) Patient-derived organoids (PDOs) as a novel in vitro model for neuroblastoma tumours. *BMC Cancer* 19: 1 – 11

Gao H, Korn JM, Ferretti S, Monahan JE, Wang Y, Singh M, Zhang C, Schnell C, Yang G, Zhang Y *et al* (2015) High-throughput screening using patient-derived tumor xenografts to predict clinical trial drug response. *Nat Med* 21: 1318 – 1325

Glosli H, Bisogno G, Kelsey A, Chisholm JC, Gaze M, Kolb F, McHugh K, Shipley J, Gallego S, Merks JHM *et al* (2021) Non-parameningeal head and neck rhabdomyosarcoma in children, adolescents, and young adults: Experience of the European paediatric Soft tissue sarcoma Study Group (EpSSG) – RMS2005 study. *Eur J Cancer* 151: 84 – 93

Grassi L, Alfonsi R, Francescangeli F, Signore M, De Angelis ML, Addario A, Costantini M, Flex E, Ciolfi A, Pizzi S *et al* (2019) Organoids as a new model for improving regenerative medicine and cancer personalized therapy in renal diseases. *Cell Death Dis* 10: 201

Greaves M, Maley CC (2012) Clonal evolution in cancer. *Nature* 481: 306 – 313

Haas BJ, Dobin A, Li B, Stransky N, Pochet N, Regev A (2019) Accuracy assessment of fusion transcript detection via read-mapping and de novo fusion transcript assembly-based methods. *Genome Biol* 20: 213

Hao Y, Hao S, Andersen-Nissen E, Mauck WM, Zheng S, Butler A, Lee MJ, Wilk AJ, Darby C, Zager M *et al* (2021) Integrated analysis of multimodal single-cell data. *Cell* 184: 3573 – 3587

Heerema-Mckenney A, Wijnaendts LCD, Pulliam JF, Lopez-Terrada D, McKenney JK, Zhu S, Montgomery K, Mitchell J, Marinelli RJ, Hart AAM *et al* (2008) Diffuse myogenin expression by immunohistochemistry is an independent marker of poor survival in pediatric rhabdomyosarcoma: a tissue microarray study of 71 primary tumors including correlation with molecular phenotype. *Am J Surg Pathol* 32: 1513 – 1522

Hettmer S, Wagers AJ (2010) Muscling in: uncovering the origins of rhabdomyosarcoma. *Nat Med* 16: 171 – 173

Hinson ARP, Jones R, Lisa LE, Belyea BC, Barr FG, Linardic CM (2013) Human rhabdomyosarcoma cell lines for rhabdomyosarcoma research: utility and pitfalls. *Front Oncol* 3: 183

Imle R, Kommoss FKF, Banito A (2021) Preclinical in vivo modeling of pediatric sarcoma—promises and limitations. *J Clin Med* 10: 1578

Jacob F, Salinas RD, Zhang DY, Nguyen PTT, Schnoll JG, Wong SZH, Thokala R, Sheikh S, Saxena D, Prokop S *et al* (2020) A patient-derived glioblastoma organoid model and biobank recapitulates inter- and intra-tumoral heterogeneity. *Cell* 180: 188 – 204

Jinek M, Chylinski K, Fonfara I, Hauer M, Doudna JA, Charpentier E (2012) A programmable dual-RNA-guided DNA endonuclease in adaptive bacterial immunity. *Science* 337: 816 – 821

Kashi VP, Hatley ME, Galindo RL (2015) Probing for a deeper understanding of rhabdomyosarcoma: insights from complementary model systems. *Nat Rev Cancer* 15: 426 – 439

Kersten K, Visser KE, Miltenburg MH, Jonkers J (2017) Genetically engineered mouse models in oncology research and cancer medicine. *EMBO Mol Med* 9: 137 – 153

Kim J, Koo BK, Knoblich JA (2020) Human organoids: model systems for human biology and medicine. *Nat Rev Mol Cell Biol* 21: 571 – 584

Kucab JE, Zou X, Morganella S, Joel M, Nanda AS, Nagy E, Gomez C, Degasperi A, Harris R, Jackson SP *et al* (2019) A compendium of mutational signatures of environmental agents. *Cell* 177: 821 – 836

Lee JM, Nair J, Zimmer A, Lipkowitz S, Annunziata CM, Merino MJ, Swisher EM, Harrell MI, Trepel JB, Lee MJ *et al* (2018) Prexasertib, a cell cycle checkpoint kinase 1 and 2 inhibitor, in BRCA wild-type recurrent high-grade serous ovarian cancer: a first-in-class proof-of-concept phase 2 study. *Lancet Oncol* 19: 207 – 215

Li J, Thompson TD, Miller JW, Pollack LA, Stewart SL (2008) Cancer incidence among children and adolescents in the United States, 2001–2003. *Pediatrics* 121: e1470-7

Liao Y, Smyth GK, Shi W (2019) The R package Rsubread is easier, faster, cheaper and better for alignment and quantification of RNA sequencing reads. *Nucleic Acids Res* 47: e47

Le Loarer F, Laffont S, Lesluyes T, Tirode F, Antonescu C, Baglin AC, Delespaul L, Soubeyran I, Hostein I, Pérot G *et al* (2019) Clinicopathologic and

Molecular Features of a Series of 41 Biphenotypic Sinonasal Sarcomas Expanding Their Molecular Spectrum. *Am J Surg Pathol* 43: 747 – 754

Lu J, Krepelova A, Rasa SMM, Annunziata F, Husak O, Adam L, Nunna S, Neri F (2021) Characterization of an in vitro 3D intestinal organoid model by using massive RNAseq-based transcriptome profiling. *Sci Rep* 11: 1 – 14

Ma CX, Cai S, Li S, Ryan CE, Guo Z, Schaiff WT, Lin L, Hoog J, Goiffon RJ, Prat A *et al* (2012) Targeting Chk1 in p53-deficient triple-negative breast cancer is therapeutically beneficial in human-in-mouse tumor models. *J Clin Invest* 122: 1541 – 1552

Manders F, Brandsma AM, de Kanter J, Verheul M, Oka R, van Roosmalen MJ, van der Roest B, van Hoeck A, Cuppen E, van Boxtel R (2022) MutationalPatterns: the one stop shop for the analysis of mutational processes. *BMC Genom* 23: 134

Manzella G, Schreck LD, Breunis WB, Molenaar J, Merks H, Barr FG, Sun W, Römmele M, Zhang L, Tchinda J *et al* (2020) Phenotypic profiling with a living biobank of primary rhabdomyosarcoma unravels disease heterogeneity and AKT sensitivity. *Nat Commun* 11: 1 – 15

Mascarenhas L, Lyden ER, Breitfeld PP, Walterhouse DO, Donaldson SS, Rodeberg DA, Parham DM, Anderson JR, Meyer WH, Hawkins DS (2019) Risk-based treatment for patients with first relapse or progression of rhabdomyosarcoma: a report from the Children's Oncology Group. *Cancer* 125: 2602 – 2609

Maura F, Degasperi A, Nadeu F, Leongamornlert D, Davies H, Moore L, Royo R, Ziccheddu B, Puente XS, Avet-Loiseau H *et al* (2019) A practical guide for mutational signature analysis in hematological malignancies. *Nat Commun* 10: 2969

Muraro MJ, Dharmadhikari G, Grün D, Groen N, Dielen T, Jansen E, van Gurp L, Engelse MA, Carlotti F, de Koning EJP *et al* (2016) A single-cell transcriptome atlas of the human pancreas. *Cell Syst* 3: 385 – 394

Obenchain V, Lawrence M, Carey V, Gogarten S, Shannon P, Morgan M (2014) VariantAnnotation: a Bioconductor package for exploration and annotation of genetic variants. *Bioinformatics* 30: 2076 – 2078

Pappo AS, Anderson JR, Crist WM, Wharam MD, Breitfeld PP, Hawkins D, Raney RB, Womer RB, Parham DM, Qualman SJ *et al* (1999) Survival after relapse in children and adolescents with rhabdomyosarcoma: a report from the intergroup rhabdomyosarcoma study group. *J Clin Oncol* 17: 3487 – 3493

Parekh S, Ziegenhain C, Vieth B, Enard W, Hellmann I (2018) zUMIs – a fast and flexible pipeline to process RNA sequencing data with UMIs. *Gigascience* 7: giy059

Patton RB, Horn RG (1962) Rhabdomyosarcoma: clinical and pathological features and comparison with human fetal and embryonal skeletal muscle. *Surgery* 52: 572 – 584

Perez EA, Kassira N, Cheung MC, Koniaris LG, Neville HL, Sola JE (2011) Rhabdomyosarcoma in children: a SEER population based study. *J Surg Res* 170: e243 – e251

Petljak M, Alexandrov LB, Brammeld JS, Price S, Wedge DC, Grossmann S, Dawson KJ, Ju YS, Iorio F, Tubio JMC *et al* (2019) Characterizing mutational signatures in human cancer cell lines reveals episodic APOBEC mutagenesis. *Cell* 176: 1282 – 1294

Pompili L, Porru M, Caruso C, Biroccio A, Leonetti C (2016) Patient-derived xenografts: a relevant preclinical model for drug development. *J Exp Clin Cancer Res* 35: 189

Ponce-Castañeda MV, García-Chéquer AJ, Eguía Aguilar P, Abundes-Ramírez MA, Hernández-Angeles A, Nieto-Martínez K, Gómez-Laguna L, Sadowinski-Pine S, Cabrera-Muñoz ML (2014) Detection of common chromosomal translocations in small round blue cell pediatric tumors. *Arch Med Res* 45: 143 – 151

Ritz C, Baty F, Streibig JC, Gerhard D (2015) Dose-response analysis using R. *PLoS ONE* 10: e0146021

Rogakou EP, Pilch DR, Orr AH, Ivanova VS, Bonner WM (1998) DNA double-stranded breaks induce histone H2AX phosphorylation on serine 139. *J Biol Chem* 273: 5858 – 5868

Rudzinski ER, Anderson JR, Hawkins DS, Skapek SX, Parham DM, Teot LA (2015) The World Health Organization classification of skeletal muscle tumors in pediatric rhabdomyosarcoma a report from the Children's Oncology Group. *Arch Pathol Lab Med* 139: 1281 – 1287

Saltsman JA, Hammond WJ, Narayan NJC, Requena D, Gehart H, Lalazar G, Laquaglia MP, Clevers H, Simon S (2020) A human organoid model of aggressive hepatoblastoma for disease modeling and drug testing. *Cancers (Basel)* 12: 1 – 18

Sato T, Stange DE, Ferrante M, Vries RGJ, Van Es JH, Van Den Brink S, Van Houdt WJ, Pronk A, Van Gorp J, Siersema PD *et al* (2011) Long-term expansion of epithelial organoids from human colon, adenoma, adenocarcinoma, and Barrett's epithelium. *Gastroenterology* 141: 1762 – 1772

Sato T, Vries RG, Snippert HJ, Van De Wetering M, Barker N, Stange DE, Van Es JH, Abo A, Kujala P, Peters PJ *et al* (2009) Single Lgr5 stem cells build crypt-villus structures in vitro without a mesenchymal niche. *Nature* 459: 262 – 265

Schindelin J, Arganda-Carreras I, Frise E, Kaynig V, Longair M, Pietzsch T, Preibisch S, Rueden C, Saalfeld S, Schmid B *et al* (2012) Fiji: an open-source platform for biological-image analysis. *Nat Methods* 9: 676 – 682

Shern JF, Chen L, Chmielecki J, Wei JS, Patidar R, Rosenberg M, Ambrogio L, Auclair D, Wang J, Song YK *et al* (2014) Comprehensive genomic analysis of rhabdomyosarcoma reveals a landscape of alterations affecting a common genetic axis in fusion-positive and fusion-negative tumors. *Cancer Discov* 4: 216 – 231

Shern JF, Selfe J, Izquierdo E, Patidar R, Chou H-C, Song YK, Yohe ME, Sindiri S, Wei J, Wen X *et al* (2021) Genomic classification and clinical outcome in rhabdomyosarcoma: a report from an International Consortium. *J Clin Oncol* 39: 2859 – 2871

Skapek SX, Ferrari A, Gupta AA, Lupo PJ, Butler E, Shipley J, Barr FG, Hawkins DS (2019) Rhabdomyosarcoma. *Nat Rev Dis Primers* 5: 1

Stewart E, Federico SM, Chen X, Shelat AA, Bradley C, Gordon B, Karlstrom A, Twarog NR, Clay MR, Bahrami A *et al* (2017) Orthotopic patient-derived xenografts of paediatric solid tumours. *Nature* 549: 96 – 100

Tickle T, Tirosh I, Georgescu C, Brown M, Haas B (2019) *inferCNV of the Trinity CTAT Project. Klarman Cell Observatory*. Cambridge, MA, USA: Broad Institute of MIT and Harvard https://github.com/broadinstitute/inferCNV

Wachtel M, Dettling M, Koscielniak E, Stegmaier S, Treuner J, Simon-Klingenstein K, Bühlmann P, Niggli FK, Schäfer BW (2004) Gene expression signatures identify rhabdomyosarcoma subtypes and detect a novel t(2;2)(q35;p23) translocation fusing PAX3 to NCOA1. *Cancer Res* 64: 5539 – 5545

Weber-Hall S, Anderson J, McManus A, Abe S, Nojima T, Pinkerton R, Pritchard-Jones K, Shipley J (1996) Gains, losses, and amplification of genomic material in rhabdomyosarcoma analyzed by comparative genomic hybridization. *Cancer Res* 56: 3220 – 3224

Van De Wetering M, Francies HE, Francis JM, Bounova G, Iorio F, Pronk A, Van Houdt W, Van Gorp J, Taylor-Weiner A, Kester L *et al* (2015) Prospective derivation of a living organoid biobank of colorectal cancer patients. *Cell* 161: 933 – 945

WHO (2020) *WHO classification of tumours: soft tissue and bone tumours*, 5th edn. Lyon: International Agency for Research on Cancer

Yamazaki S, Ohka F, Hirano M, Shiraki Y, Motomura K, Tanahashi K, Tsujiuchi T, Motomura A, Aoki K, Shinjo K *et al* (2021) Newly established patient-derived organoid model of intracranial meningioma. *Neuro Oncol* 23: 1936 – 1948

 © 2022 The Authors

Yan C, Brunson DC, Tang Q, Do D, Iftimia NA, Moore JC, Hayes MN, Welker AM, Garcia EG, Dubash TD *et al* (2019) Visualizing engrafted human cancer and therapy responses in immunodeficient zebrafish. *Cell* 177: 1903 – 1914

Yang J, Ren Z, Du X, Hao M, Zhou W (2014) The role of mesenchymal stem/progenitor cells in sarcoma: update and dispute. *Stem cell Investig* 1: 18

Yohe ME, Gryder BE, Shern JF, Song YK, Chou HC, Sindiri S, Mendoza A, Patidar R, Zhang X, Guha R *et al* (2018) MEK inhibition induces MYOG and remodels super-enhancers in RAS-driven rhabdomyosarcoma. *Sci Transl Med* 10: eaan4470

Yonish-Rouach E, Resnftzky D, Lotem J, Sachs L, Kimchi A, Oren M (1991) Wild-type p53 induces apoptosis of myeloid leukaemic cells that is inhibited by interleukin-6. *Nature* 352: 345 – 347

Yuki K, Cheng N, Nakano M, Kuo CJ (2020) Organoid models of tumor immunology. *Trends Immunol* 41: 652 – 664

License: This is an open access article under the terms of the Creative Commons Attribution License, which permits use, distribution and reproduction in any medium, provided the original work is properly cited.

Expanded View Figures

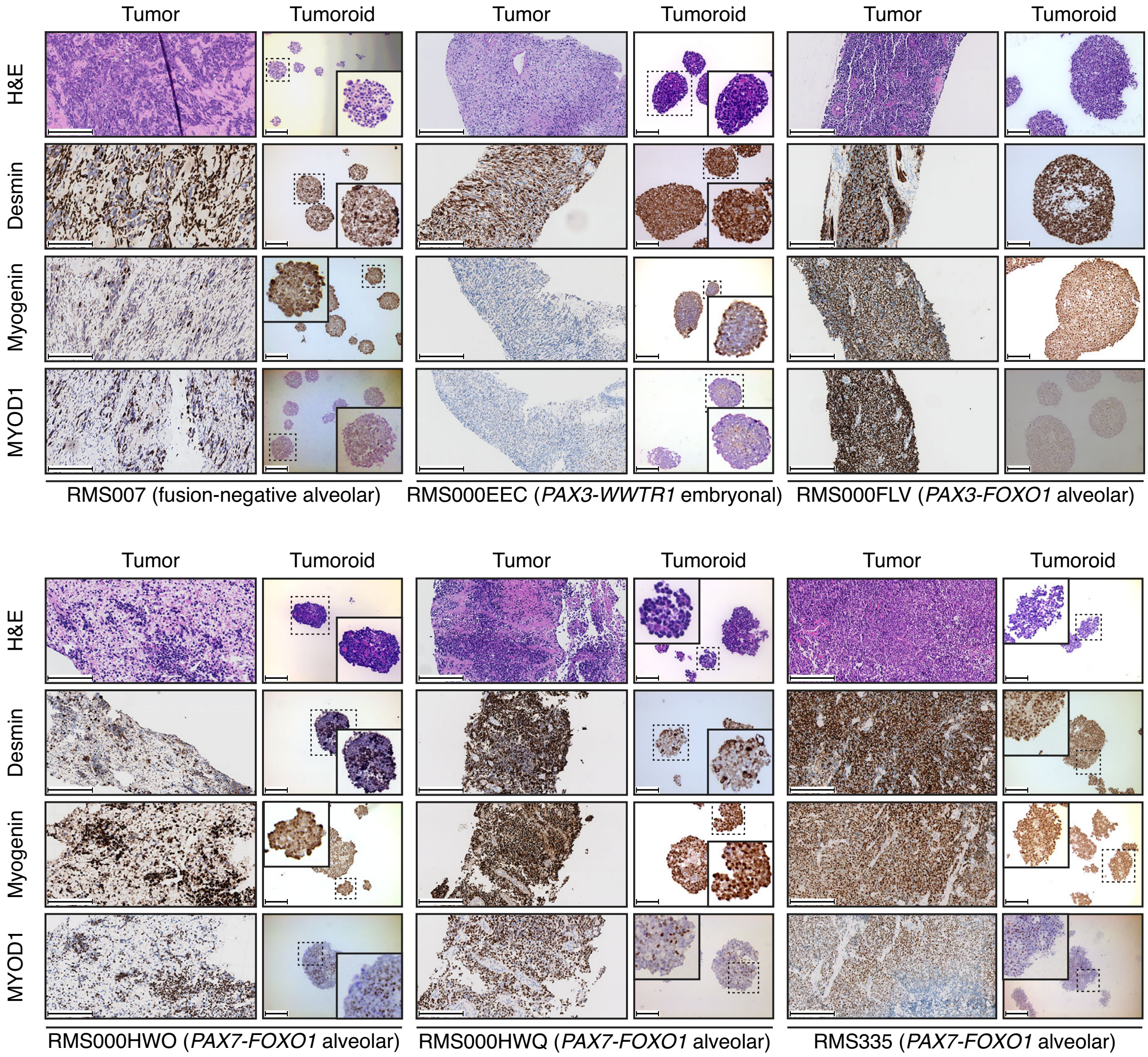

Figure EV1. Retained marker protein expression (additional figures).

Morphological (via H&E) and immunohistochemical (IHC) comparison of RMS tumors and derived RMS tumoroid models shows retained marker protein (Desmin, Myogenin, and MYOD1) expression and cellular morphology. Scale bars equal 200 µm (RMS007, RMS000HWO, RMS335) or 100 µm (RMS000EEC, RMS000FLV, RMS000HWQ). For RMS tumoroid models that form small spheres a zoom-in image of a sphere was inserted.

 © 2022 The Authors

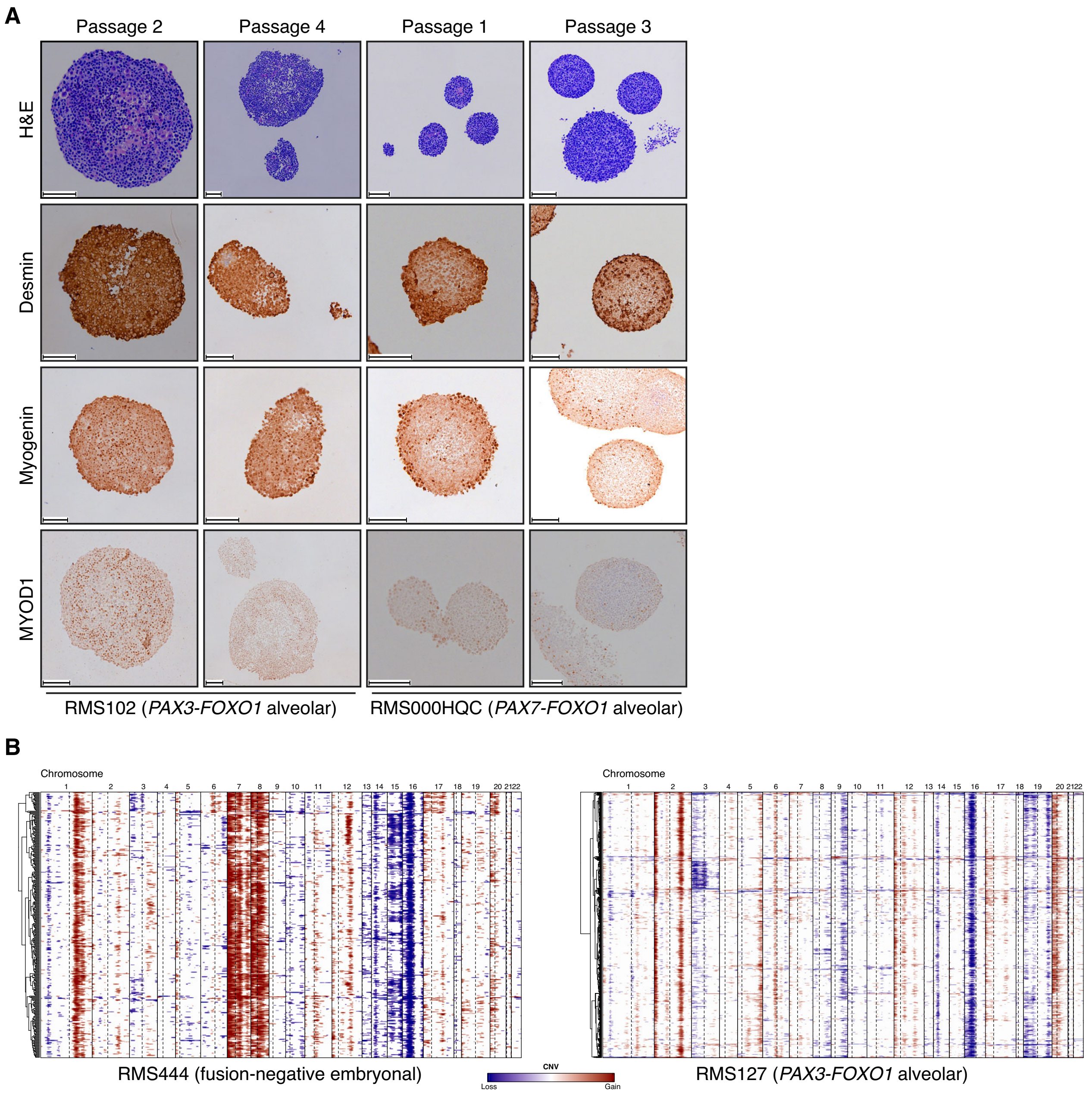

Figure EV2. Marker protein expression during early passages of culturing and single-cell RNA-seq inferred per cell CNV profiles.

A H&E and immunohistochemistry (IHC) stainings of early passage RMS102 (passage 2 and passage 4 – passage 6 used for drug screening) and RMS000HQC (passage 1 and passage 3 – passage 5 used for drug screening). Scale bars equal 100 μm.

B Heatmaps showing the inferred CNV profiles of single cells (*y*-axis) from the RMS127 and RMS444 tumoroid models, respectively. Chromosome arms are delineated by dotted lines where applicable.

Figure EV3. RMS tumoroid models molecularly resemble the tumor they are derived from (additional figures).

A Examples of circular copy number plots of fusion-negative RMS with embryonal (RMS000ETY) or alveolar (RMS007) histology. Outer circle depicts the tumoroid model, inner circle depicts the tumor.

B Examples of circular copy number plots of fusion-positive RMS with alveolar histology and *PAX3-FOXO1* fusion (RMS410) or *PAX7-FOXO1* fusion (RMS000HQC). Outer circle depicts the tumoroid model, inner circle depicts the tumor.

C Contribution of averaged single base substitution (SBS) profiles for RMS tumors (upper row) and tumoroid models (lower row).

D Clustered correlogram of SBS profiles detected in RMS tumors (T) and tumoroid models (O).

E Comparison of total number of mutations (synonymous and nonsynonymous) in RMS tumors and tumoroid models per fusion-type (fusion-negative $n = 4$, fusion-positive = 15, Wilcoxon test, $P = 0.15$ for tumors, $P = 0.02$ for tumoroid models). Data are shown as boxplots: The central line in the box represents the median; the upper and lower limits of the box represent the third and first quartile, respectively; the whiskers represent the minimum (bottom) and maximum (top) values excluding outliers, which are plotted as individual points.

 © 2022 The Authors

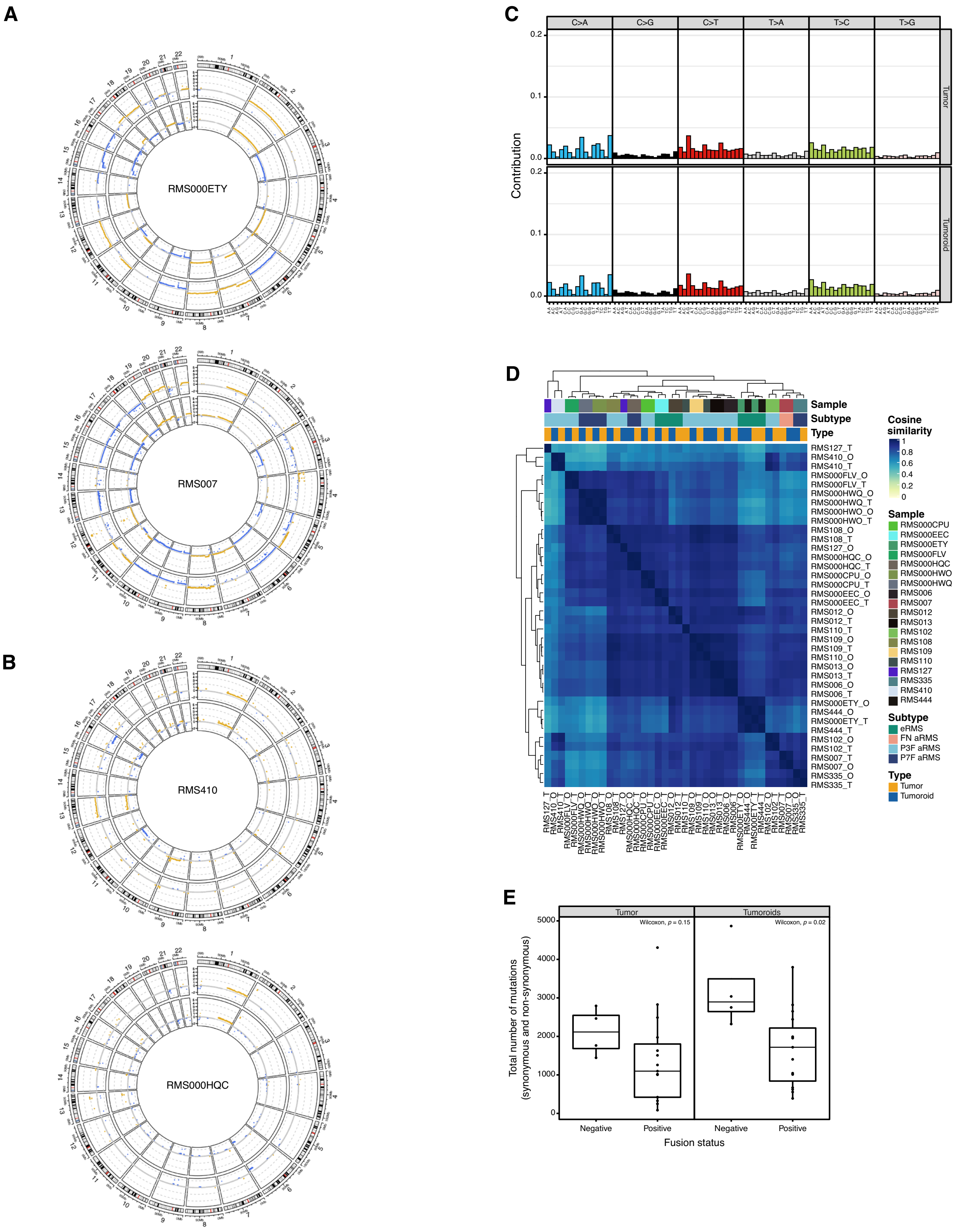
A
RMS000ETY
RMS007
B
RMS410
RMS000HQC
C
C>A
C>G
C>T
T>A
T>C
T>G
Contribution
Tumor
Tumoroid
D
Sample
Subtype
Type
Cosine similarity
Sample
RMS000CPU
RMS000EEC
RMS000ETY
RMS000FLV
RMS000HQC
RMS000HWO
RMS000HWQ
RMS006
RMS007
RMS012
RMS013
RMS102
RMS108
RMS109
RMS110
RMS127
RMS335
RMS410
RMS444
Subtype
eRMS
FN aRMS
P3F aRMS
P7F aRMS
Type
Tumor
Tumoroid
E
Tumor
Tumoroids
Wilcoxon, p = 0.15
Wilcoxon, p = 0.02
Total number of mutations (synonymous and non-synonymous)
Negative
Positive
Fusion status

Figure EV3.

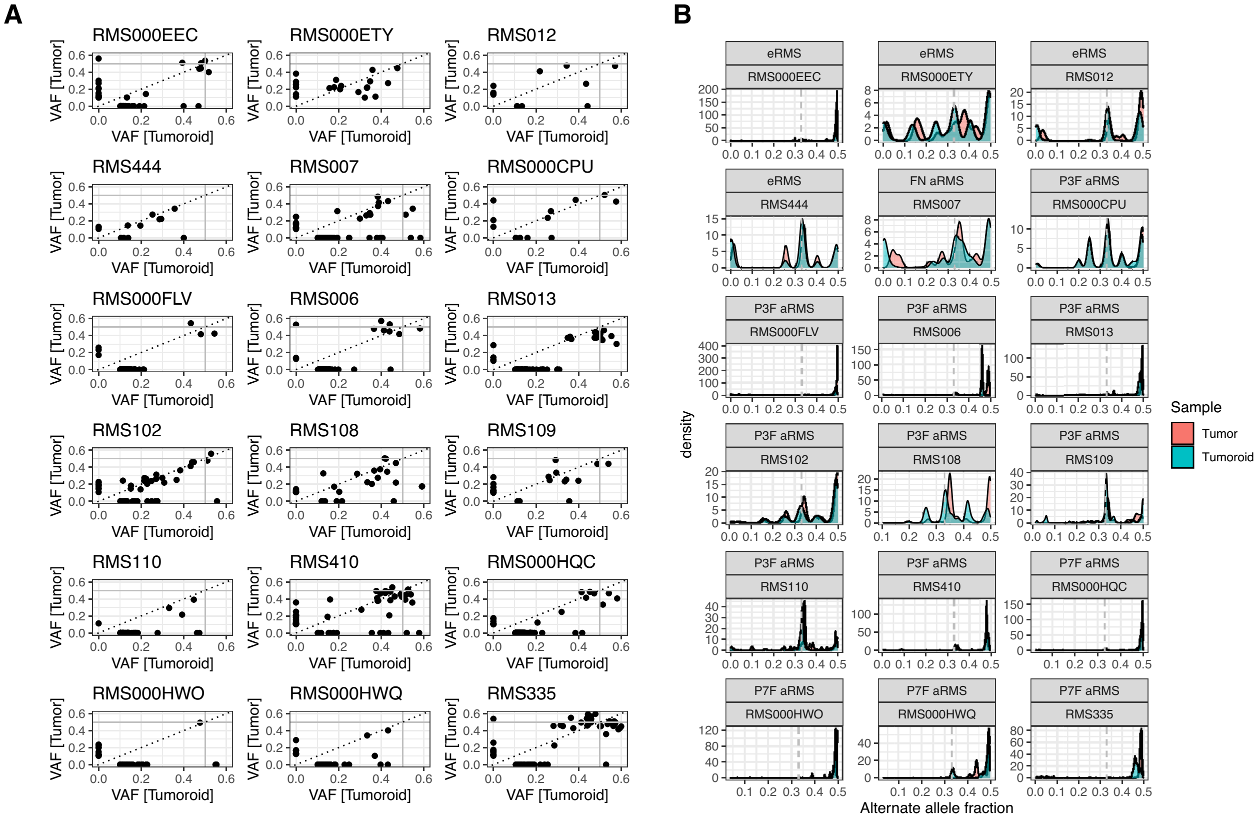

Figure EV4. Established RMS tumoroid models maintain the clonal composition of the RMS tumor they were derived from to a large extent.

A Correlogram of variant allele fraction (VAF) of single-nucleotide variants (SNVs) detected in RMS tumors and derived tumoroid models per pair.

B Density plots of alternate allele (B-allele) fraction over the entire genome per RMS tumor (red) and derived RMS tumoroid model (blue) per pair.

 © 2022 The Authors

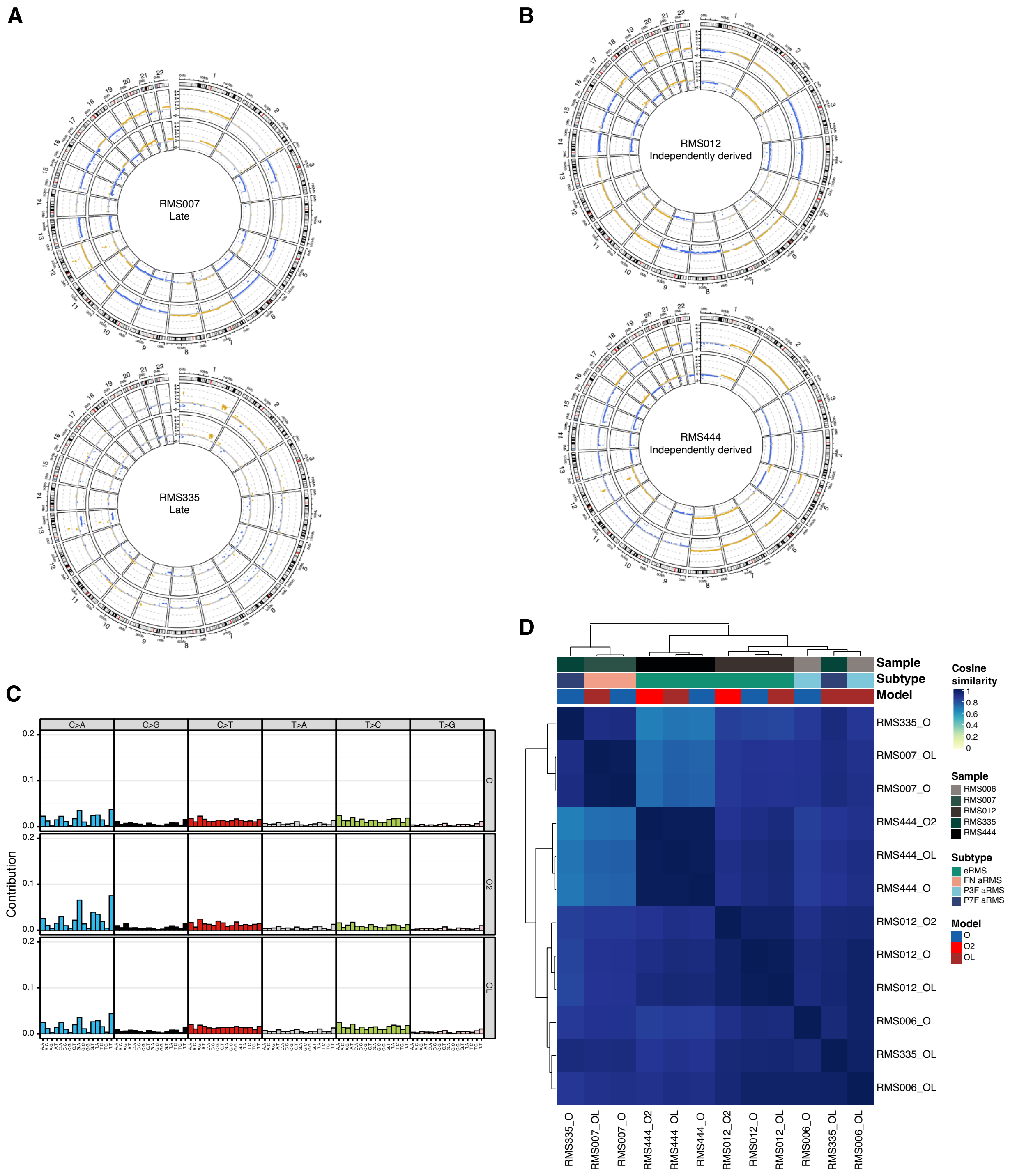

Figure EV5. Genetic and transcriptional stability of tumoroid models over time (additional figures).

A Circular copy number plots of standard and late passage RMS tumoroid models of a fusion-negative alveolar (RMS007) and a *PAX7-FOXO1* positive alveolar (RMS335) RMS. Outer circle depicts the standard passage tumoroid, inner circle depicts the late passage tumoroid model.

B Circular copy number plots of standard passage and independently derived RMS tumoroid models of two fusion-negative embryonal RMS (RMS012 and RMS444). Outer circle depicts the standard passage tumoroid, inner circle depicts the independently derived tumoroid model.

C Contribution of averaged single base substitution (SBS) profiles for standard (O), independently derived (O2), and late passage (OL) RMS tumoroid models.

D Clustered correlogram of SBS profiles detected in standard (O), independently derived (O2), and late passage (OL) RMS tumoroid models.

A

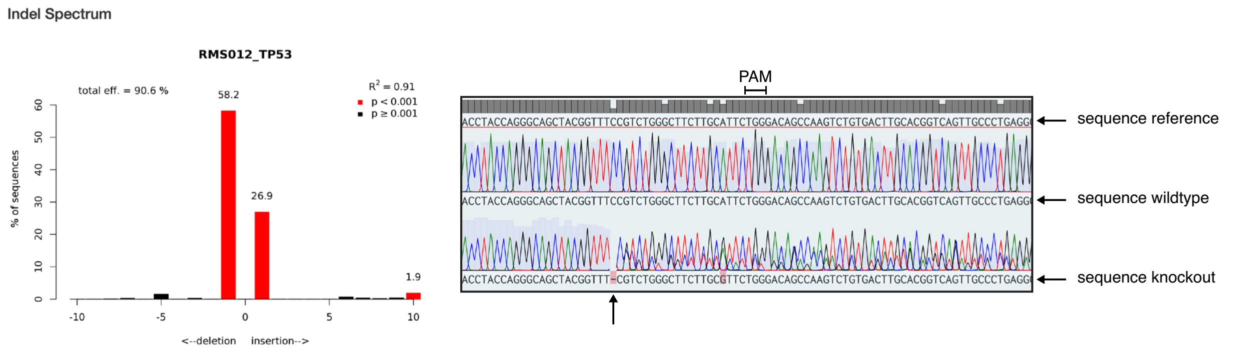

B

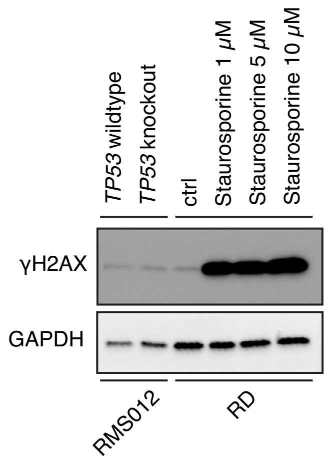

Figure EV6. RMS tumoroid models can be molecularly edited using CRISPR/Cas9 with P53-deficient eRMS being more sensitive to the checkpoint kinase inhibitor prexasertib (additional figures).

A Left panel: Inferred Indel Spectrum from Sanger sequencing using the TIDE tool (https://tide.nki.nl). Right panel: Sanger sequencing around Cas9 cut site (arrow at the bottom) in *TP53* gene. Protospacer Adjacent Motif (PAM) sequence indicated.

B Western Blotting analysis of γH2AX (Ser-139) as proxy for DNA double-strand breaks in untreated RMS012 tumoroid cells (*TP53* wildtype and *TP53* knockout). RMS cell line RD treated with staurosporine served as positive control while GAPDH served as loading control.

Source data are available online for this figure.

 © 2022 The Authors

Appendix

Table of contents

- Appendix Figure S1: Correlation analysis of Area Under the Curve (AUC) values from technical replicates of drug screenings

Appendix Figure S1

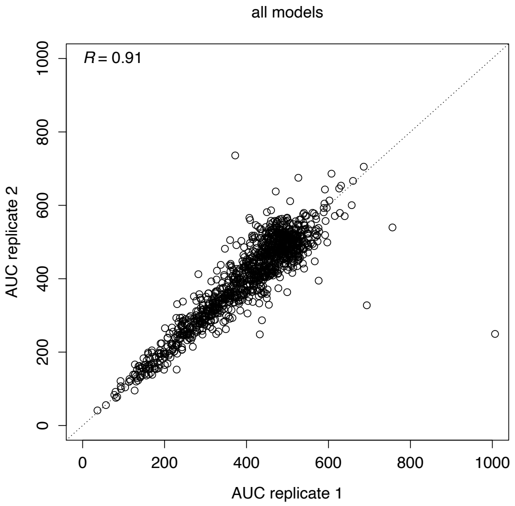

Appendix Figure S1 legend: Correlation analysis of Area Under the Curve (AUC) values (n = 165) from technical replicates of drug screenings of RMS007, RMS109, RMS110, RMS000EEC, RMS000FLV, RMS000HQC, and RMS000HWQ. Overall, the correlation coefficient (*R*) is 0.91 (Pearson).
