# Supplementary material for: Mesenchymal tumor organoid models recapitulate rhabdomyosarcoma subtypes
Source: EMBO Mol Med. 2022 Aug 2;14(10):e16001. doi: 10.15252/emmm.202216001 (PMC9549731; doi:10.15252/emmm.202216001)
Supplement: Supplementary file 9 — Source Data for Figure 6 [file EMMM-14-e16001-s002.zip › figure_6/6B/6B_readme.rtf]

Left side: P53 detectionRight side: GAPHD detectionLane 1: RMS012 TP53 wild type cellsLane 2: RMS012 TP53 knockout cellsLane 3: control cellsSame for both replicates (rep1 and rep2)
