# Supplementary material for: Mesenchymal tumor organoid models recapitulate rhabdomyosarcoma subtypes
Source: EMBO Mol Med. 2022 Aug 2;14(10):e16001. doi: 10.15252/emmm.202216001 (PMC9549731; doi:10.15252/emmm.202216001)
Supplement: Supplementary file 10 [file EMMM-14-e16001-s008.pdf]

EMBO Molecular Medicine

Mesenchymal tumor organoid models recapitulate rhabdomyosarcoma subtypes

Michael Meister, Marian Groot Koerkamp, Terezinha de Souza, Willemijn Breunis, Ewa Frazer-Mendelewska, Mariël Brok, Jeff DeMartino, Freek Manders, Camilla Calandrini, Hindrik Kerstens, Alex Janse, M. Dolman, Selma Eising, Karin Langenberg, Marc van Tuil, Rutger Knops, Sheila Terwisscha van Scheltinga, Laura Hiemcke-Jiwa, Uta Flucke, Johannes Merks, Max van Noesel, Bastiaan Tops, Jayne Hehir-Kwa, Patrick Kemmeren, Jan Molenaar, Marc van de Wetering, Ruben van Boxtel, Jarno Drost, and Frank Holstege
DOI: 10.15252/emmm.202216001

Corresponding authors: Jarno Drost (J.Drost@prinsesmaximacentrum.nl) , Frank Holstege (F.C.P.Holstege@prinsesmaximacentrum.nl)

Review Timeline:

Transfer from Review Commons:	11th Mar 22
Editorial Decision:	7th Apr 22
Revision Received:	18th Jun 22
Editorial Decision:	27th Jun 22
Revision Received:	11th Jul 22
Accepted:	12th Jul 22

Editor: Lise Roth

Transaction Report:

Review COMMONS

This manuscript was transferred to EMBO Molecular Medicine following peer review at Review Commons.

(Note: With the exception of the correction of typographical or spelling errors that could be a source of ambiguity, letters and reports are not edited. Depending on transfer agreements, referee reports obtained elsewhere may or may not be included in this compilation. Referee reports are anonymous unless the Referee chooses to sign their reports.)

Review #1

1. Evidence, reproducibility and clarity:

Evidence, reproducibility and clarity (Required)

Summary:

Meister et al. set out to develop a new organoid preclinical model of rhabdomyosarcoma (RMS). The authors comment that this system would be beneficial for preclinical modeling because it has the ability to maintain the tumor's molecular characteristics. The authors then proved that organoids derived from multiple RMS subtypes resembled their parent tumors using RT-qPCR for characteristic markers, histopathology, copy number profiles, mutational signature analyses, and transcriptional profiling. Importantly, the authors performed long term studies to show that the organoids remain stable over multiple passages and do not change their mutational landscape dramatically. Finally, the authors tested their organoids with known RMS therapeutics and for their ability to be engineered with the CRISPR/Cas9 system. Not surprisingly, the authors found their organoids sensitive to known RMS therapeutics and were successfully able to generate TP53-/- organoids with CRISPR/Cas9, underscoring this organoid system in translatable use. This report nicely describes a method for the establishment of human RMS organoid culture systems that can be leveraged for preclinical testing.

Major Comments:

1. The authors provided a foundational validation of their organoids through various methods, and their protocol stands to impact the field of RMS biology. To validate the organoids as recapitulating the primary human tumors, the authors perform analysis on the bulk organoid and bulk human primary tumors. The authors showed through sequencing efforts that the bulk mutational profile and transcriptional profiles do not dramatically change between the parent tumor and organoids. This analysis was done well; however, the authors fail to rigorously illustrate that the organoids maintain tumor cell heterogeneity of the primary human tumors. To rigorously validate the organoid system, the authors should illustrate the organoid culture conditions do not alter the heterogeneity of cells (cell plasticity) compared to that of the primary tumor. A formal assessment of the cellular plasticity in the organoids to the primary tumor would determine how the organoid system either maintains or shifts the cancer cell plasticity because of changes in microenvironment (Oncogene, 2020, 39: 2055-2068). The addition scRNA-seq would illustrate whether the organoids maintain the same populations as the primary tumor or bias for the propagation of specific cell populations at a single cell level and provide more rigorous information about every cell type present.
2. The authors took great strides to show that the organoids respond to therapeutics similarly to primary tumors. However, Figure 5A could be more transparent with more data labelled in the graph instead of just in the app and the implications of the variable responses could have been explored in the discussion section. Furthermore, for this model to be clinically relevant for pharmacokinetic studies, propagation in mice needs to be shown.
3. Figure 1 is well put together to graphically demonstrate the process by which organoids were obtained and manipulated. Figure 1B, however, as a graphical summary is a little

confusing, and the information would be greatly enhanced by the addition of a comprehensive table. Furthermore, additional information could be added to the table to make it a more inclusive and impactful addition to the paper.
4. It is quite impactful that the authors were able to actively engineer the organoids with CRISPR/Cas9 and accurately delete TRP53, but controls were not represented in the figure. The experiment should have included a sgRNA targeting a pan-essential gene as a positive control and a non-targeting sgRNA as a negative control. We recommend addition of both controls to the experiment outlined in Figure 6 to increase the validity and rigor of the data presented.
5. Although the authors provide an insight into a useful preclinical RMS model, the paper lacks mechanistic insight besides cursory description of the model.

Minor Comments

1. Figure 3C and 4B are not transparent in their labels and could be altered so that every line has an associated gene in the publication. Furthermore, there are sample specific differences that could be explored in the discussion.
2. In Supplementary Figure 1, higher magnification inserts are needed to get a closer look at the IHC. Furthermore, the white balance is not the same in all the images and needs to be corrected prior to publication. The difference in white balance can clearly be seen in the last panels depicting IHC for RMS335, where the MYOD1 staining has a yellow background whereas the H&E staining has a white background.
3. The authors mentioned in line 202 that some of their organoids contain the novel fusion of PAX3 and WWTR1, but this fusion is not indeed novel as it has previously been seen in biphenotypic sinonasal sarcoma (Am J Surg Pathol 2019, 43:747-754).

2. Significance:

Significance (Required)

As has been mentioned previously, this research is impactful to the field of RMS biology because the authors were successfully able to use organoid technology, which has not previously been reported. The authors do a great job of listing current RMS modelling techniques and explaining how their system addresses the pitfalls of the others. Furthermore, this protocol could be expanded to the development of other organoid systems for other sarcomas. The rhabdomyosarcoma field and larger sarcoma community would be keenly interested in this work. It is clear that this system has the potential for use in pre-clinical settings as well as in high-throughput screens, but further validation and increased rigor is required on both fronts.

It is astounding and the authors should be complimented that they were able to show a median time from patient to drug screen was 81 days! This has enormous potential such as rapid translation of therapies and personalized medicine. That said, the authors must first refine the heterogeneity of the organoids and demonstrate how the organoids reflect the phenotypic and cellular plasticity of the parent tumors. Furthermore, the authors ought to be careful when making priority claims. The authors state that this is the first system to use organoids but should recognize the advances demonstrated by Manzella et al. (Nat Commun, 2020, 11:4629). Additionally, the authors state that this is the first demonstration of pre-clinical models harboring FGFR4V550L mutations; this fails to recognize the prior reported

work by several groups (Chen et al., Cancer Cell, 2013, 24:710-24; Manzella et al., Nat Commun, 2020, 11:4629; McKinnon et al., Oncogene, 2018, 37:2630-2644).

3. How much time do you estimate the authors will need to complete the suggested revisions:

Estimated time to Complete Revisions (Required)

(Decision Recommendation)

Between 3 and 6 months

Review #2

1. Evidence, reproducibility and clarity:

Evidence, reproducibility and clarity (Required)

This manuscript describes the possibility to generate a collection of pediatric rhabdomyosarcoma (RMS) tumor organoid models comprising broad spectrum subtypes from highly aggressive to extremely rare. The authors were able very successfully establish 19 RMS models from 46 pediatric RMS patient samples with 41 % efficiency. All RMS tumoroid models were thoroughly characterized and retained the molecular characteristics of the tumor they are derived from as well as they displayed genetic stability over time. Most of the tested tumors showed long-term propagation potential, reaching passage 40 and remaining stable. Though, establishing time for RMS tumoroid models varied with a median time from acquisition of the tumor sample to successful drug screening being 81 days, highly aggressive tumors were established in as little as 27 days. Also, authors shown us in elegant manner the suitability of RMS tumoroid models for research in two specific ways: via drug screening and CRISPER/Cas9 genome editing.

2. Significance:

Significance (Required)

In summary, the author's work made significant progress in 3D culture and tumor organoid models of mesenchymal origin, being the first collection of tumoroid models from mesenchymal malignant tumors and the second thoroughly characterized tumoroid collection specific for pediatric cancers. Without doubt, biobanked collection of RMS tumoroids will be useful for drug screening as well as molecular editing. Also, these models will be a useful resource for future research and in preclinical and clinical testing therapeutics for RMS. In the future, organoids generated from patients with RMS may lead to precise and personalized treatment.

3. How much time do you estimate the authors will need to complete the suggested revisions:

Estimated time to Complete Revisions (Required)

(Decision Recommendation)

Less than 1 month

Review #3

1. Evidence, reproducibility and clarity:

Evidence, reproducibility and clarity (Required)

Meister et al., describe their methodology in establishing what they term organoid or tumoroid 2D/3D cultures derived from samples of rhabdomyosarcoma (RMS) patient tumours. They have success to varying degrees across the subtypes with greater success in those more clinically aggressive. Their analyses of markers, the somatic genetics and gene expression profiles suggest that they are largely representative of RMS and the tumor samples from which they were derived. Their utility in drug screening and manipulation by knocking out TP53 by CRISP/Cas9 is also demonstrated. The conclusion is that this represents a useful approach for generating patient derived models and a unique resource for preclinical analyses and other research into RMS.

This is a major piece of work that is well written and presented. The link to interrogate the data worked. I have only a few comments.

Major comments

The authors describe the models derived as organoids/tumoroids implying that multiple cell types are represented potentially recreating the tumor microenvironment. Can the authors comment more specifically and demonstrate the extent to which cell types in addition to the tumor cells are represented, viable and are organized through analyses of the original and tumoroid sections (extend fig 2C/supplementary fig) and via analyses of the RNAseq data?

Does the quantification from the RT-qPCR analyses for the MYOD1, MYOG and Desmin of the models match that in the samples from which they were derived? Does the RNAseq that was performed on tumour and the culture at the time of the drug screen tie in with this?

How do the frequencies of SNVs compare with recent studies? Or are the numbers in the risk groups not appropriately represented?

Minor comments

The number of models and success rates would be useful to indicate in the abstract.

It would be helpful to define the SBS1, 5,and 18 in the figure legends. Do the age related signatures in any way correlate with patient age or aggressivness of tumors?

Page 13 line 300 just because the RH30 cell line has TP53 mutation doesn't mean that it was acquired in culture - unless there is specific evidence that supports this.

2. Significance:

Significance (Required)

The significance of this study is in describing how a relatively large number of models of RMS were established plus increasing awareness of the biobank resource and associated data that has been created. The approach, although used in more ad hoc reports of smaller numbers of RMS, represents a useful development for mesenchymal tumors versus the more established development of such models in epithelial cancers. Although a lower success rate than xenografts, it is a useful and practical cost-effective alternative for preclinical testing and research. Likely interest to a speciaclist audience for those involved in the RMS, sarcoma and pediatric cancer field.

Referees cross-commenting

OK with the balance of comments for the authors to address. I think the extent to which they are prepared to address the heterogeneity issue, and the results of this for the models, is likely to affect the impact of their study.

3. How much time do you estimate the authors will need to complete the suggested revisions:

Estimated time to Complete Revisions (Required)

(Decision Recommendation)

Between 1 and 3 months

Full Revision

Manuscript number: RC-2022-01227
Corresponding author(s): Frank Holstege

1. General Statements

The reviewers are enthusiastic. They agree with the claims made and comment favorably with regard to the impact as well on the short- and long-term potential for translation. All three go out of their way to emphasize positive aspects. A variety of questions were raised and we submit a complete revision with point-by-point replies that addresses all of these. This includes addressing tumor organoid (tumoroid) plasticity (reviewer #1) and composition/heterogeneity (reviewer #3) by incorporating single cell data as well as other analyses. We thank the reviewers for the thorough feedback. The additional data, analyses and clarifications strengthen the study.

To keep the rebuttal as short as possible we have only copied the reviewers' concerns/questions, not the favorable comments. The copied remarks are in normal font. Our replies are in italics. Each question is accompanied by a reply and a brief description of changes made in response.

2. Point-by-point description of the revisions

Reviewer #1, Major Comment #1: **"**The authors provided a foundational validation of their organoids through various methods, and their protocol stands to impact the field of RMS biology. To validate the organoids as recapitulating the primary human tumors, the authors perform analysis on the bulk organoid and bulk human primary tumors. The authors showed through sequencing efforts that the bulk mutational profile and transcriptional profiles do not dramatically change between the parent tumor and organoids. This analysis was done well; however, the authors fail to rigorously illustrate that the organoids maintain tumor cell heterogeneity of the primary human tumors. To rigorously validate the organoid system, the authors should illustrate the organoid culture conditions do not alter the heterogeneity of cells (cell plasticity) compared to that of the primary tumor. A formal assessment of the cellular plasticity in the organoids to the primary tumor would determine how the organoid system either maintains or shifts the cancer cell plasticity because of changes in microenvironment (Oncogene, 2020, 39: 2055-2068). The addition scRNA-seq would illustrate whether the organoids maintain the same populations as the primary tumor or bias for the propagation of specific cell populations at a single cell level and provide more rigorous information about every cell type present."

Reply: *The reviewer's question is whether the tumor cells in the tumoroid culture have the same degree of plasticity and are therefore as heterogeneous in culture as they are in the tumors that*

they are derived from. We agree that evaluating the heterogeneity of tumor cells in the tumoroid culture is desirable. This would ensure that the procedure has not simply selected for a single type of tumor cell. We have therefore generated single-cell RNA sequencing (scRNA-seq) data of tumoroid cells as suggested. It is important to point out that a complete inventory of RMS tumor cell heterogeneity by scRNA-seq has not been published as yet. Such an undertaking, i.e., scRNA-seq of a large cohort of RMS tumors, is an entire study in itself and lies outside the scope of this study. It would also not be feasible due to limited sample material for many of the tumors used here. Nevertheless, as is being alluded to by the reviewer, there is ample evidence of tumor cell heterogeneity in primary RMS tumors based on previous studies using immunohistochemistry (for example the well-known heterogeneity in expression of RMS marker proteins such as Myogenin, MyoD1 and Desmin). As shown in the figure below (included in the revision as Fig. 2D), when cultured as tumoroid models, examples from both of the main tumor types (FP-RMS sample RMS127 and FN-RMS sample RMS444) show a large degree of heterogeneity in expression of the known, heterogeneously expressed tumor cell markers Myogenin (MYOG gene), MyoD1 (MYOD1 gene) and Desmin (DES gene). Comparison with the cell cycle marker Ki-67 (MKI67 gene) shows that this heterogeneity is not due the cells being present in different cell cycle phases.

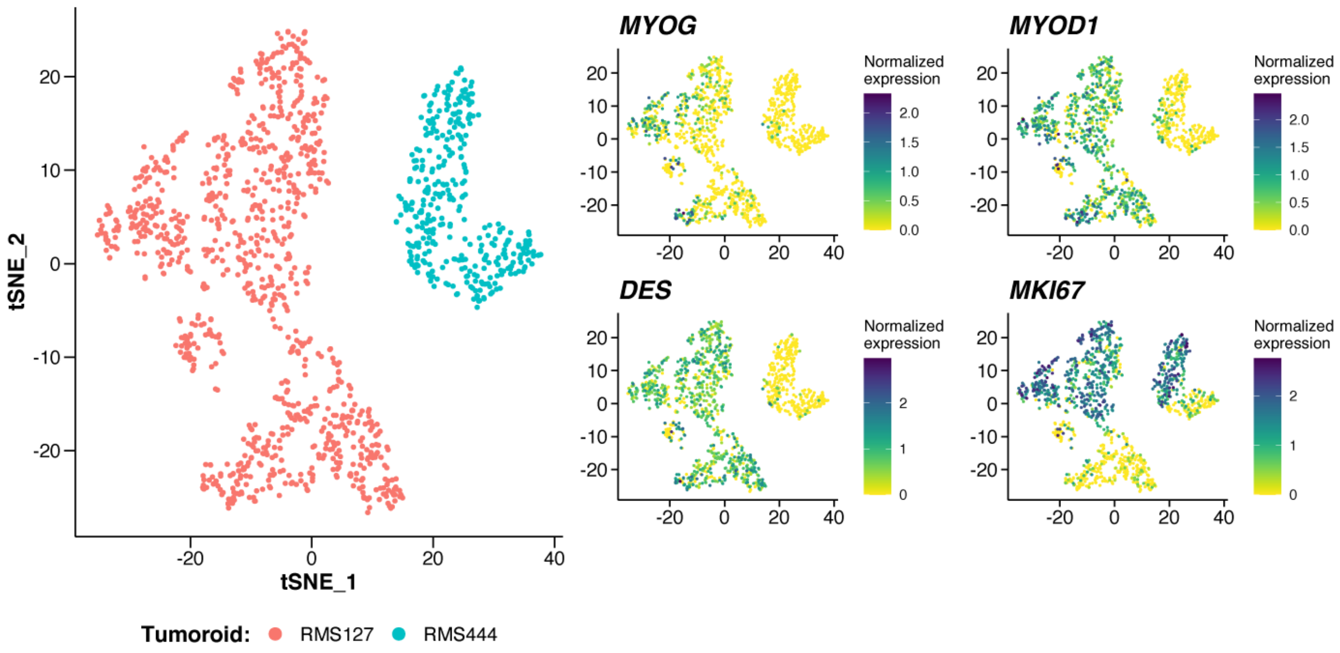

Tumor cell heterogeneity in the tumoroid culture is further indicated by the heterogeneous CNV patterns derived from the tumoroid scRNA-seq data (Figure below, included in revision as Suppl. Fig. 1B).

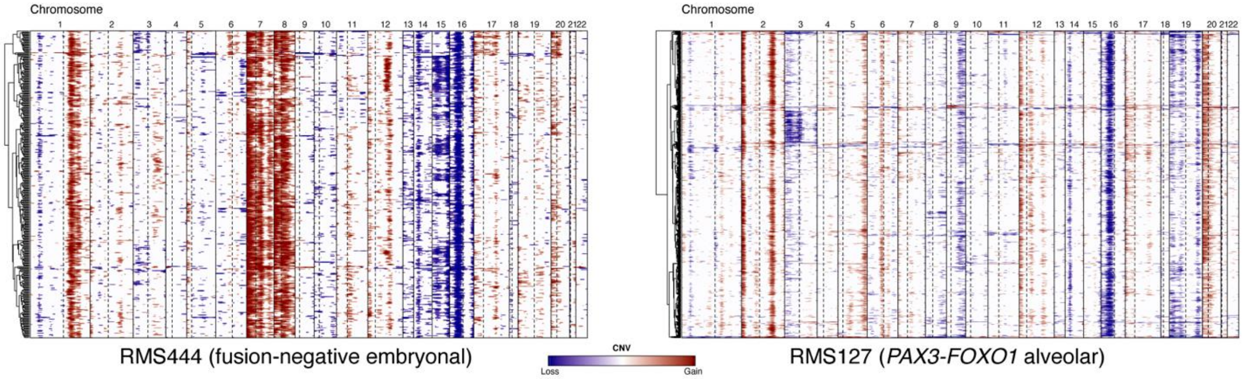

Both the CNV analysis and the scRNA-seq marker gene expression indicate that the tumoroid culture conditions neither stringently select for a single type of tumor cell, nor drive the tumor cells into a uniform expression pattern phenotype, consistent with maintaining plasticity, even after the 7 (RMS127) and 5 (RMS444) passages. These are good indications of retained plasticity/heterogeneity. Additionally, we make it clear in the revised version that a more exhaustive answer would benefit from having a complete cohort of tumor scRNA-seq data to first determine the degree of heterogeneity exhibited by RMS tumors for all genes.

The related question of tumoroid cellular composition, with regard to the presence of non-tumor cells, is addressed in response to reviewer #3, major comment #1.

Changes: *Addition of Fig. 2D and Suppl. Fig. 1B with figure captions. Additional text in the Results and the Discussion sections. Additions to the Methods for the generation and analysis of the scRNA-seq data.*

Reviewer #1, Major Comment #2: “The authors took great strides to show that the organoids respond to therapeutics similarly to primary tumors. However, Figure 5A could be more transparent with more data labelled in the graph instead of just in the app and the implications of the variable responses could have been explored in the discussion section. Furthermore, for this model to be clinically relevant for pharmacokinetic studies, propagation in mice needs to be shown.”

Reply:
- We have made Fig. 5A more transparent by adding the drug names.
- The different response between FN-RMS and FP-RMS subtypes for certain drugs is known and the implication that the models reflect this is discussed more thoroughly now as suggested.
- We agree that animal experiments are imperative for pharmacokinetic studies of new drugs. However, most of the drugs that were included here, especially the ones highlighted, have already been evaluated in early phase clinical trials in adults and/or children. The pharmacokinetic data for humans is therefore already available for these drugs, making

additional animal studies for pharmacokinetics of these drugs redundant. For future studies, various types of animal studies are likely to be required and we make this clear in the Discussion, also emphasizing that in general, tumoroid models do propagate in mice. To address this specifically for RMS, we have started a collaboration to generate PDX mouse models derived from RMS tumor and tumoroid samples in parallel. Anecdotally we can state here that at least 50% propagate. However, since we wish to investigate this systematically and with a complete set of tumoroid models, it is not prudent to wait for these results before publishing the current study. This would delay making the protocols, findings and tumoroid models available to the scientific community and as our (and many other groups') work exemplifies, tumoroid models can yield important findings on their own.
Changes: *Drug names added to rows in Fig. 5A. The Discussion has been expanded to include the differential response of tumor subtypes and tumoroids to different drugs and to include the uses (including pharmacokinetics) of different types of models has been expanded.*

Reviewer #1, Major Comment #3: "Figure 1 is well put together to graphically demonstrate the process by which organoids were obtained and manipulated. Figure 1B, however, as a graphical summary is a little confusing, and the information would be greatly enhanced by the addition of a comprehensive table. Furthermore, additional information could be added to the table to make it a more inclusive and impactful addition to the paper."

Reply: *We agree.*
Changes: *A new Table 1 has been added as a separate file with a corresponding revised legend in the main document.*

Reviewer #1, Major Comment #4: "It is quite impactful that the authors were able to actively engineer the organoids with CRISPR/Cas9 and accurately delete TRP53, but controls were not represented in the figure. The experiment should have included a sgRNA targeting a pan-essential gene as a positive control and a non-targeting sgRNA as a negative control. We recommend addition of both controls to the experiment outlined in Figure 6 to increase the validity and rigor of the data presented."

Reply: *We respectfully note that all appropriate controls were done. This included a non-targeting sgRNA as negative control (see Methods lines 1137 to 1140). As also explained in Fig. 6A, the strategy for generating a P53 knock-out involved selection through nutlin-3 exposure, whereby cells wildtype for P53 are selected against. As described (Methods lines 1144 to 1146), cells transfected with the non-targeting sgRNA plasmid indeed died upon nutlin-3 exposure. A sgRNA against a pan-essential gene was not included in this strategy since the nutlin-3 already kills all cells with a wildtype P53. Finally, we draw attention to the fact that the success of the approach was assessed by Western Blotting (Fig. 6B) and Sanger sequencing (Suppl. Fig. 6A).*
Changes: *None.*

Reviewer #1, Major Comment #5: "Although the authors provide an insight into a useful preclinical RMS model, the paper lacks mechanistic insight besides cursory description of the model."

Reply: *Insight into a wide variety of different molecular and cellular mechanisms will be exciting to explore in future studies. This publication is indeed focused on describing an approach that works for RMS, and therefore showing for the first time that this works systematically for mesenchymal-derived tumors. In addition, the study describes key characteristics of the tumoroid models that are important to establish their validity as models and that are essential to demonstrate before making the tumoroid models available to the wider scientific community in order to perform the further mechanistic analyses. The word cursory is in contrast to the many positive comments made by this reviewer and the other two reviewers with regard to the extensive characterization.*

Changes*: None.*

Reviewer #1, Minor Comment #1: "Figure 3C and 4B are not transparent in their labels and could be altered so that every line has an associated gene in the publication. Furthermore, there are sample specific differences that could be explored in the discussion."

Reply: *We agree.*

Changes: *Gene names have been added for every row in both figures. The Discussion now incorporates the observed differences.*

Reviewer #1, Minor Comment #2: "In Supplementary Figure 1, higher magnification inserts are needed to get a closer look at the IHC. Furthermore, the white balance is not the same in all the images and needs to be corrected prior to publication. The difference in white balance can clearly be seen in the last panels depicting IHC for RMS335, where the MYOD1 staining has a yellow background whereas the H&E staining has a white background."

Reply: *We agree.*

Changes: *Higher magnification inserts have now been provided throughout Suppl. Fig. 1A. The white balance has been corrected.*

Reviewer #1, Minor Comment #3: "The authors mentioned in line 202 that some of their organoids contain the novel fusion of PAX3 and WWTR1, but this fusion is not indeed novel as it has previously been seen in biphenotypic sinonasal sarcoma (Am J Surg Pathol 2019, 43:747-754)."

Reply: *We rephrased this to clarify that this is the first report of such a fusion in RMS, rather than in general.*
Changes: *The corresponding sentence has been rephrased.*

Reviewer #1, Comment within the Significance Statement: "The authors state that this is the first system to use organoids but should recognize the advances demonstrated by Manzella et al. (Nat Commun, 2020, 11:4629). Additionally, the authors state that this is the first demonstration of pre-clinical models harboring FGFR4V550L mutations; this fails to recognize the prior reported work by several groups (Chen et al., Cancer Cell, 2013, 24:710-24; Manzella et al., Nat Commun, 2020, 11:4629; McKinnon et al., Oncogene, 2018, 37:2630-2644)."

Reply: *We had in fact already recognized the advances described by Manzella et al. which was referenced in two places in the original submission (current lines 100 and 388). We thank the reviewer for pointing out the previous work done on an RMS cell line that harbored an FGFR4 p.V550L mutation.*
Changes: *We rephrased the corresponding passages concerning the FGFR4 mutation.*

We thank reviewer #1 for all the comments. This has resulted in many improvements.

Reviewer #2:
We thank reviewer #2 for the positive comments. There are no major/minor queries to address.

Reviewer #3, Major Comment #1: "The authors describe the models derived as organoids/tumoroids implying that multiple cell types are represented potentially recreating the tumor microenvironment. Can the authors comment more specifically and demonstrate the extent to which cell types in addition to the tumor cells are represented, viable and are organized through analyses of the original and tumoroid sections (extend fig 2C/supplementary fig) and via analyses of the RNAseq data?"

Reply: *We use the term tumor organoid or tumoroids as coined by the field in general. This indeed indicates a degree of self-organization such as the three-dimensional growth in spheres and the propagation of a heterogeneous population of tumor cells (see comment #1, reviewer 1) for example. In general, however, tumoroids do not include growth of a non-tumor cell microenvironment inter-woven with the (different types of) tumor cells. Exceptions to this are very early passage tumoroids that are not yet stable and which may still contain non-tumor cells, or specialized co-culture conditions that are currently being actively sought to allow for co-culture of tumor cells within a non tumor cell microenvironment. It is therefore not anticipated that late passage tumoroid models will have non-tumor cells. The basis of the technology is that the defined set of growth factors in the medium mimics the tumor stimulating conditions of the*

non-tumor cell microenvironment. Since the mixed presence of tumor and non-tumor cells generally gives rise to one (frequently the non-tumour cell) outgrowing the other, it is often considered the hallmark of an unsuccessful tumoroid.

The reviewer therefore raises an important point that we have failed to make clear. We have addressed this in two ways. We emphasize that the scRNA-seq data that are now included in response to reviewer #1, comment #1 do not indicate the presence of any non-tumor cells (as expected). In addition, this aspect of tumor organoid technology is explained better in the Introduction.
Changes: *The results section has been expanded with the description of the scRNA-seq data emphasizing the expected lack of non-tumor cells and the introductory section on tumor organoid technology has been improved to make it clear that currently this generally involves growth of different types of tumor cells only.*

Reviewer #3, Major Comment #2: “Does the quantification from the RT-qPCR analyses for the MYOD1, MYOG and Desmin of the models match that in the samples from which they were derived? Does the RNAseq that was performed on tumor and the culture at the time of the drug screen tie in with this?”

Reply: *The answer is yes. The figure below shows tumor and tumoroid bulk RNA seq of those genes also analyzed by RT-qPCR (i.e., DES, MYOG, and MYOD1). Note that this is also the same stage as for the drug screening. As can be appreciated, the expression of these markers is generally very comparable between tumors and the derived tumoroid models. Note that this also constitutes a nice independent (albeit indirect) verification of the similar degree of heterogeneity issue raised by Reviewer #1 (comment #1). Expression of the markers was lower in the tumoroid models of RMS000HQC and RMS000ETY compared to the primary tumor. In line with this, expression of these genes was also already lower in the early passages of the culture as determined by RT-qPCR (Fig. 2A). Nevertheless, copy-number analysis inferred from whole-genome sequencing showed that the resulting tumoroid models are indeed tumor cells (Suppl. Fig. 2A top panel and Suppl. Fig. 2B lower panel).*

We therefore conclude that the expression of probed marker genes is generally comparable between tumor and tumoroid and that early passage RT-qPCR based expression analysis of these markers can be reflective of the expression in the fully established model.

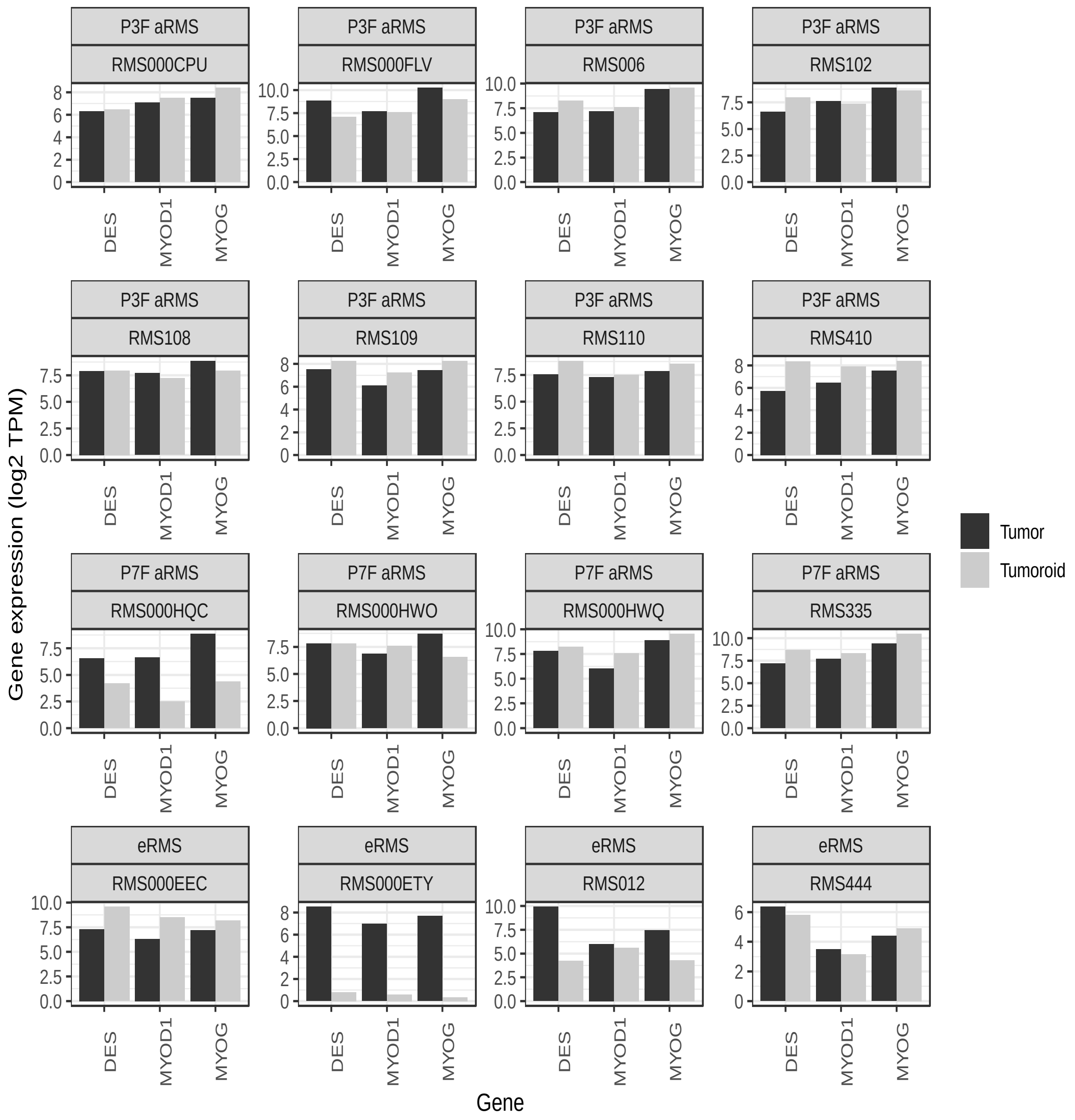

Changes: *None. The expression data are already available within the interactive browser-based Shiny App.*

Reviewer #3, Major Comment #3: "How do the frequencies of SNVs compare with recent studies? Or are the numbers in the risk groups not appropriately represented?"

Reply: *The SNV frequencies are quite comparable to recent studies, with similar differences between risk groups, all as depicted in the figure below, now included as Suppl. Fig 2E in the revision.*

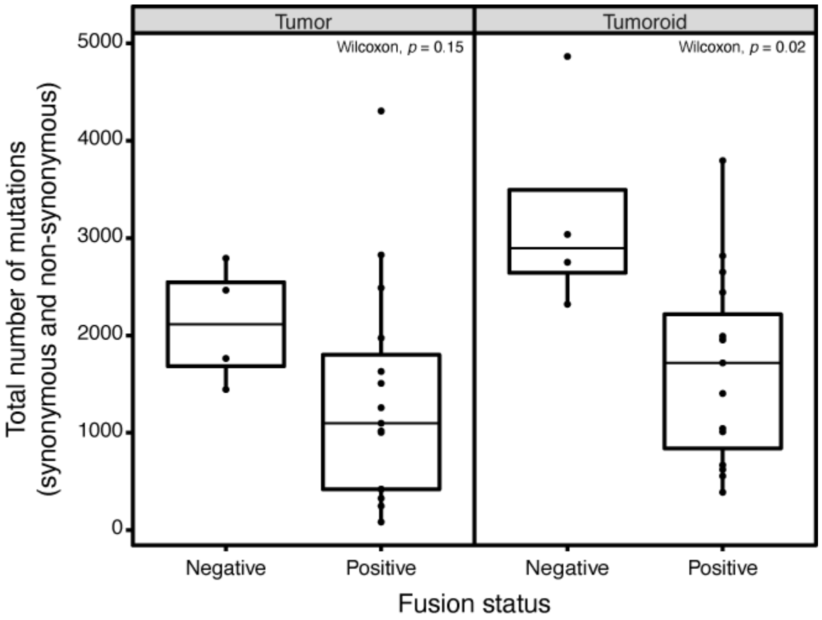

The SNV frequency was calculated from our WGS data following the procedure from the most recent report in pediatric cancer (https://www.biorxiv.org/content/10.1101/2021.09.28.462210v1). Across tumor and tumoroid models we found a somatic mutation frequency of SNVs with a VAF of >0.3 ranging from 0.03 to 1.92 mut/MB (median 0.70 mut/MB) which is comparable to the reported somatic mutation frequency in the afore-mentioned study (median 0.9 mut/MB in RMS). Concerning the risk groups, a recent study (https://pubmed.ncbi.nlm.nih.gov/31699828/) found a significant difference in the tumor mutational burden between fusion-negative (FN) and fusion-positive (FP) RMS (2.6 mut/MB vs. 1.0 mut/MB, respectively) with a higher mutational burden associated with poorer outcome. In our study, the FN-RMS tumoroid models also show a higher mutation frequency compared to the FP-RMS tumoroid models (FN 4 vs. FP 15, p = 0.02, Wilcoxon). Such a difference is also found between the original tumors but without statistical difference (FN 4 vs. FP 15, p = 0.15, Wilcoxon) likely related to the small sample sizes. This underscores the representative nature of the tumoroid models and is of obvious interest to include. We have made the appropriate changes.

Changes: *To include these analyses in the manuscript, we added a new Suppl. Fig. 2E with corresponding Suppl. Fig. legend and a new paragraph in the main text.*

Reviewer #3, Minor Comment #1: “The number of models and success rates would be useful to indicate in the abstract.”

Reply: We agree.
Changes: We added this information to the abstract.

Reviewer #3, Minor Comment #2: "It would be helpful to define the SBS1, 5,and 18 in the figure legends. Do the age related signatures in any way correlate with patient age or aggressivness of tumors?"

Reply:
- Agreed. The definitions of SBS1, 5, and 18 have now been included the legends of Fig. 3B and 4A.
- The age-related signatures SBS1 (but not SBS5) shows a weak albeit significant correlation with patient age only in RMS tumoroid models but not in RMS tumors. Furthermore, concerning aggressiveness, FP-RMS tumors and tumoroid models show a significantly higher contribution of SBS1 (but not SBS5) to their overall somatic mutation frequency compared to FN-RMS tumors and tumoroid models. However, since FP-RMS tumor samples were obtained from older patients (median 14 years versus median 6 years in FN-RMS tumor samples), this observation could also be related to the patient-age and not primarily to the fusion-status. The heterogeneity of samples (e.g., primary therapy-naïve samples versus relapse and therefore pre-treated samples) and the relatively low sample number could be explanations for the lack of a stronger correlation in general.
Changes: *Added definitions of SBS1, 5, and 18 in the legends of Fig. 3B and 4A. Added text in the Results section to indicate the observed correlations.*

Reviewer #3, Minor Comment #3: "Page 13 line 300 just because the RH30 cell line has TP53 mutation doesn't mean that it was acquired in culture - unless there is specific evidence that supports this."

Reply: *We thank the reviewer for this rectification. To our knowledge, there is indeed no specific evidence that this cell line acquired the TP53 mutation during culturing or whether the mutation was already present in the primary tumor the cell line was derived from.*
Changes: *The corresponding statement has been removed.*

We thank reviewer #3 for all the comments. This has resulted in many improvements.

Besides the changes described above, additional minor changes were made:

We have moved the interactive, browser-based Shiny app to a server that is managed by our institute instead of having it hosted on shinyapps.io. We include the new URL in line 556.

The data upload to the European Genome-Phenome Archive (EGA) of the data from the initial submission has been completed and the raw sequencing data can now be accessed. The data upload of the scRNA-seq data generated for the revision is currently ongoing. We have therefore adapted and renamed the "Bulk sequencing data availability" section in the Methods in the manuscript (lines 1043 to 1050).

We updated the code available at https://github.com/teresouza/rms2018-009 following the additional analyses performed for the revision.

Supplementary Table 1: The values for row "RMS000FLV" for columns "sample_body_site" and "primary_site_specific" were corrected as this tumor was located in the upper leg and not the upper arm of the patient. Furthermore, we added patient numbers as in the new Table 1 and corrected spelling errors. This does not change any of the conclusions in the manuscript.

Figure 6A: The protein "P53" was spelled without capital "P" in the initial version. We corrected this.

We included the recently described Zebrafish RMS PDX models (https://pubmed.ncbi.nlm.nih.gov/31031007/) in the Discussion of RMS models. See lines 507 to 510.

With the addition of Fig. 2D, the figure legends of Fig. 2A and 2B were moved to the side (Fig. 2A) or below (Fig. 2B) the figure. With the addition of the single-cell copy-number plots, Suppl. Fig. 1 was divided in Suppl. Fig. 1A and 1B.

Some of the original scale bars in Fig. 2C and Suppl. Fig. 1A were incorrectly labelled and this has now been corrected. This does not change any of the conclusions.

Minor corrections in the sections Affiliations, Financial support, Author contributions and Conflict of Interests.

7th Apr 2022

Dear Dr. Holstege,

Thank you for the submission of your manuscript to EMBO Molecular Medicine. We have now received the enclosed reports from the three referees who were invited to review your manuscript (and who had initially reviewed it for Review Commons).

As you will see from the reports below, while referees #1 and #3 are satisfied with your revisions, referee #2 (initially referee #1) still raises a major concern that should be addressed for further consideration of your manuscript in EMM.

In a following correspondence and upon your request, this referee further clarified the nature of his/her concern and stated: "Tumor populations are not a single clone. It is now clear that solid tumors including sarcomas are a heterogenous population representing of cells representing different clonal populations and cell states. This heterogeneity is exquisitely illustrated by single cell analysis of primary human tumors. If the authors aim to develop a system that recapitulates the primary human tumor, they should illustrate that the tumor cell heterogeneity of the primary tumor is maintained in their organoid system. It tempting to speculate and reasonable to assume just as in adherent culture there will be a selection for clones through passage in organoids. If they propose to leverage the organoid system to inform treatment, it would mandate knowledge that the system maintains the heterogeneity of the clonal diversity of the primary tumor. In the previous comment, I acknowledged that they might not have the primary tumors to perform this analysis, but suggested an alternative by determining the clonal heterogeneity of the organoids from passage 1 to the timepoint of drug analysis. This comment is not referring to the tumor microenvironment, but to the actual clonal nature of the tumor cells themselves."

If you feel you can satisfactorily address this point, you may wish to submit a revised version of your manuscript. The revised manuscript will once again be subject to review and we cannot guarantee at this stage that the eventual outcome will be favorable. Should you find that the requested revisions are not feasible and prefer, therefore, to submit your paper elsewhere, we would welcome a message to this effect.

When submitting your revised manuscript, please carefully review the instructions that follow below. We perform an initial quality control of all revised manuscripts before re-review; failure to include requested items will delay the evaluation of your revision.

We require:

1) A .docx formatted version of the manuscript text (including legends for main figures, EV figures and tables). Please make sure that the changes are highlighted to be clearly visible.

2) Individual production quality figure files as .eps, .tif, .jpg (one file per figure). For guidance, download the 'Figure Guide PDF' (https://www.embopress.org/page/journal/17574684/authorguide#figureformat).

3) A .docx formatted letter INCLUDING the reviewers' reports and your detailed point-by-point responses to their comments. As part of the EMBO Press transparent editorial process, the point-by-point response is part of the Review Process File (RPF), which will be published alongside your paper.

4) A complete author checklist, which you can download from our author guidelines (https://www.embopress.org/page/journal/17574684/authorguide#submissionofrevisions). Please insert information in the checklist that is also reflected in the manuscript. The completed author checklist will also be part of the RPF.

5) It is mandatory to include a 'Data Availability' section after the Materials and Methods. Before submitting your revision, primary datasets produced in this study need to be deposited in an appropriate public database, and the accession numbers and database listed under 'Data Availability'. Please remember to provide a reviewer password if the datasets are not yet public (see https://www.embopress.org/page/journal/17574684/authorguide#dataavailability).
In case you have no data that requires deposition in a public database, please state so in this section. Note that the Data Availability Section is restricted to new primary data that are part of this study.

6) For data quantification: please specify the name of the statistical test used to generate error bars and P values, the number (n) of independent experiments (specify technical or biological replicates) underlying each data point and the test used to calculate p-values in each figure legend. The figure legends should contain a basic description of n, P and the test applied. Graphs must include a description of the bars and the error bars (s.d., s.e.m.). Please indicate exact p values in your figures or figure legends.

7) We would also encourage you to include the source data for figure panels that show essential data. Numerical data should be provided as individual .xls or .csv files (including a tab describing the data). For blots or microscopy, uncropped images should

be submitted (using a zip archive if multiple images need to be supplied for one panel). Additional information on source data and instruction on how to label the files are available at
.

8) We replaced Supplementary Information with Expanded View (EV) Figures and Tables that are collapsible/expandable online. A maximum of 5 EV Figures can be typeset. EV Figures should be cited as 'Figure EV1, Figure EV2" etc... in the text and their respective legends should be included in the main text after the legends of regular figures.
- For the figures that you do NOT wish to display as Expanded View figures, they should be bundled together with their legends in a single PDF file called *Appendix*, which should start with a short Table of Content. Appendix figures should be referred to in the main text as: "Appendix Figure S1, Appendix Figure S2" etc.
- Additional Tables/Datasets should be labeled and referred to as Table EV1, Dataset EV1, etc. Legends have to be provided in a separate tab in case of .xls files. Alternatively, the legend can be supplied as a separate text file (README) and zipped together with the Table/Dataset file.
See detailed instructions here:
.

9) The paper explained: EMBO Molecular Medicine articles are accompanied by a summary of the articles to emphasize the major findings in the paper and their medical implications for the non-specialist reader. Please provide a draft summary of your article highlighting
- the medical issue you are addressing,
- the results obtained and
- their clinical impact.
This may be edited to ensure that readers understand the significance and context of the research. Please refer to any of our published articles for an example.

10) Conflict of interest: We updated our journal's competing interests policy in January 2022 and request authors to consider both actual and perceived competing interests. Please review the policy https://www.embopress.org/competing-interests and update your competing interests if necessary.

11) Every published paper now includes a 'Synopsis' to further enhance discoverability. Synopses are displayed on the journal webpage and are freely accessible to all readers. They include a short stand first (maximum of 300 characters, including space) as well as 2-5 one-sentences bullet points that summarizes the paper. Please write the bullet points to summarize the key NEW findings. They should be designed to be complementary to the abstract - i.e. not repeat the same text. We encourage inclusion of key acronyms and quantitative information (maximum of 30 words / bullet point). Please use the passive voice. Please attach these in a separate file or send them by email, we will incorporate them accordingly.
Please also suggest a striking image or visual abstract to illustrate your article as a PNG file 550 px wide x 300-600 px high.

12) As part of the EMBO Publications transparent editorial process initiative (see our Editorial at http://embomolmed.embopress.org/content/2/9/329), EMBO Molecular Medicine will publish online a Review Process File (RPF) to accompany accepted manuscripts.
In the event of acceptance, this file will be published in conjunction with your paper and will include the anonymous referee reports, your point-by-point response and all pertinent correspondence relating to the manuscript. Let us know whether you agree with the publication of the RPF and as here, if you want to remove or not any figures from it prior to publication.
Please note that the Authors checklist will be published at the end of the RPF.

EMBO Molecular Medicine has a "scooping protection" policy, whereby similar findings that are published by others during review or revision are not a criterion for rejection. Should you decide to submit a revised version, I do ask that you get in touch after three months if you have not completed it, to update us on the status.

I look forward to receiving your revised manuscript.

With kind regards,

Lise

Lise Roth, PhD
Editor
EMBO Molecular Medicine

***** Reviewer's comments *****

Referee #1 (Remarks for Author):

Dear Sir/Madam,

In our first review, we did not raise any major questions to the authors of the manuscript. In addition, the authors have fixed some problems in the revised manuscript that were submitted by other reviewers. It strengthened the manuscript and made the paper more suitable to a publishable high standard. So, our feedback about the acceptability of the revised manuscript is very positive.

We found that the questions studied in this manuscript are very interesting, opening avenues to further questions and further research. This publication is focused on describing of new methodology in establishing RMS tumoroids, mesenchymal-derived tumors. In addition, the paper describes key characteristics of the tumoroid models that are important in the process of establishing tumoroids. So, this new approach of making tumoroid models will benefit future studies to better understand the different molecular and cellular mechanisms of cancers and is likely to be of interest to a wide range of readers.

Referee #2 (Comments on Novelty/Model System for Author):

The authors have developed a method to generate organoid cultures from pediatric rhabdomyosarcoma patient tumors. Their method is efficient and rapid and could be of interest and utility to the field. However, the validation provided does not adequately illustrate that he organoids maintain the heterogeneous characteristics of the primary as claimed. This would be necessary not only to illustrate that the system is better than typical adherent cell lines, but to insure that there was no significant selective pressure of their culturing conditions. Furthermore, this study provides no mechanistic insight into RMS biology or treatment, but is simply a new method. A recent manuscript in EMBO Molecular Medicine reported a similar culture system but went further to perform a drug screen identified a Hsp90 inhibitor and then went further to validate the biology and translated the findings back to humans. This current manuscript is not near the rigor of the recent paper.
EMBO Mol Med (2022)14:e14552
https://doi.org/10.15252/emmm.202114552

Referee #2 (Remarks for Author):

The authors have now added single cell RNA-seq data but missed the point of our original question. The question posed to the authors was to not to perform a rigorous single cell investigation into the organoids, but rather to illustrate whether the organoids displayed the cellular heterogeneity of the original paper as claimed. As well, illustrating how the heterogeneity is maintained (or not) over time would be critical to characterization of the organoid model. If the primary tumors are unavailable, performing assessment of heterogeneity over time in organoid culture would address if there is selective pressure for organoid growth. The increased success and rapid establishment of the reported organoid models is a major advantage over traditional cell lines, but the utility of data from the cell organoid in drug screens to be translated back to patients is likely going to be dependent on how well the passaged organoid reflect the original tumor. This remains unclear. All other major and minor comments were addressed.

Referee #3 (Remarks for Author):

I think my and other comments have been adequately addressed

***

Rev_Com_number: RC-2022-01227
New_manu_number: EMM-2022-16001
Corr_author: Holstege
Title: Mesenchymal tumor organoid models recapitulate rhabdomyosarcoma subtypes

Rebuttal letter

Comment by referee #2 (initially #1):

"Tumor populations are not a single clone. It is now clear that solid tumors including sarcomas are a heterogenous population representing of cells representing different clonal populations and cell states. This heterogeneity is exquisitely illustrated by single cell analysis of primary human tumors. If the authors aim to develop a system that recapitulates the primary human tumor, they should illustrate that the tumor cell heterogeneity of the primary tumor is maintained in their organoid system. It tempting to speculate and reasonable to assume just as in adherent culture there will be a selection for clones through passage in organoids. If they propose to leverage the organoid system to inform treatment, it would mandate knowledge that the system maintains the heterogeneity of the clonal diversity of the primary tumor. In the previous comment, I acknowledged that they might not have the primary tumors to perform this analysis, but suggested an alternative by determining the clonal heterogeneity of the organoids from passage 1 to the timepoint of drug analysis. This comment is not referring to the tumor microenvironment, but to the actual clonal nature of the tumor cells themselves."

We thank the reviewer for this helpful comment. We addressed the question of retained clonal heterogeneity on the genetic level using two different approaches. First, we determined clonal heterogeneity on the genetic level by extensive analyses of matching whole-genome sequencing (WGS) data of the primary rhabdomyosarcoma (RMS) tumors and the derived RMS tumoroid models. We compared variant allele fractions (VAFs) of pathogenic single-nucleotide variants (SNVs) between matching tumor and tumoroid models at the time of drug screening. By restricting the analysis to SNVs in genomic regions without copy-number alterations or loss of heterozygosity, we could identify SNVs characterizing the major clone in the RMS tumor sample (VAF ~ 0.5). Consequently, SNVs with a lower VAF in the tumor represent subclones. Importantly, VAFs of major as well as subclonal SNVs are largely retained in the majority of the derived tumoroid models at similar VAFs, indicating that our models do retain the clonal composition of the tumor they were derived from to a high extent. We present these new analyses in Expanded View Fig. 3B and discuss it in the text as follows:

"Tumors are composed of different tumor cell clones that can undergo processes such as clonal expansion, genetic diversification as well as clonal selection (Greaves & Maley, 2012). To assess to what degree established RMS tumoroid models reflect the clonal composition of the tumor they were derived from, we performed two analyses on the matching WGS data.
First, we compared the VAFs of SNVs in coding regions which showed no copy-number alterations or loss of heterozygosity (LOH) (Figure EV3B). For the majority of samples, SNVs with a VAF of around 0.5 (indicating a major clone in the population given the filtering criteria for SNVs described above) can be detected in tumor and tumoroid, indicating that major clones are retained. Furthermore, the presence of SNVs with VAFs of below 0.5 indicate subclonal populations. Again, such SNVs with matching VAFs in tumor and tumoroid samples can be detected, indicating that subclonal populations are also largely retained in the established RMS tumoroid models. In addition, a subset of SNVs present in the tumoroid with a high VAF is not present in the tumor with the chosen cut-offs, indicating that our method may enrich for such clones. Importantly, none of these SNVs are found in reported oncogenic driver genes, indicating that these are likely passenger mutations." (lines 313 to 329)

"A subset of SNVs present in the tumor are not detected in the established RMS tumoroid which can be due to (i) this clone not being present in the tissue piece that was used for the establishment of the tumoroid, and/or

(ii) the respective clone was present in the piece but got lost over the course of culturing and/or (iii) the sequencing depth for the tumoroid model was not sufficient to pick up this mutation (30X coverage in the tumoroid model versus 90X coverage in the tumor, respectively).” (lines 571 to 577)

For convenience, we also included the figure panel below (the afore-mentioned new Expanded View Fig. 3B):

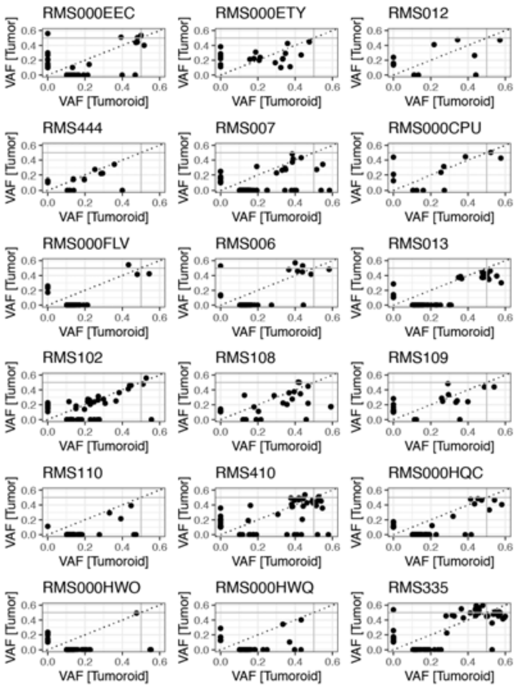

Second, we compared the fraction of alternate alleles (B-alleles) per tumor/tumoroid pair in the new Expanded View Figure 3, panel C. Patterns present in the tumor are retained in the derived tumoroid, indicating that the relative contributions of clones with varying B-allele fractions is retained in the established models. We included the following text in the manuscript:

“In the second analysis performed on the matching WGS data, fractions of alternate alleles (B-alleles) were compared between matching tumors and tumoroid models (Figure EV3C). Importantly, patterns present in the tumor are retained in the derived tumoroid, indicating that the relative contributions of clones with varying B-allele fractions is retained in the established models. Minor shifts between the peaks are likely the result of the tumor sample being impure, i.e., containing normal cells, whereas the tumoroid models consist purely of tumor cells.
Taken together, these analyses indicate that established RMS tumoroid models maintain the genetic characteristics including the clonal composition of the RMS tumor they were derived from to a large extent.” (lines 330 to 339)

For convenience, we also included the figure panel below (the afore-mentioned new Expanded View Fig. 3C):

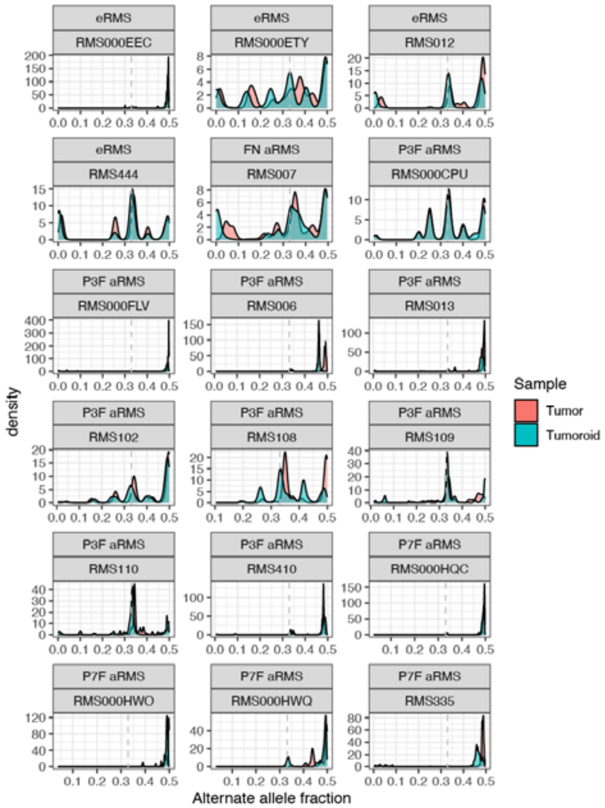

Finally, we performed additional H&E as well as immunohistochemical (IHC) stainings of two different passages of two RMS tumoroid models of early passages (i.e., before drug screening) to verify that the RMS tumoroids maintain cellular heterogeneity. Importantly, the observed heterogeneity of marker protein expression was highly concordant with the primary tumor as well as the tumoroid model at the time of drug screening, indicating that there are no major changes concerning the composition of tumor cells acquired over the course of expanding the culture for drug screening. We included these analyses in the new Expanded View Figure 3, panel A and discuss them in the manuscript as follows:

"Lastly, additional H&E and IHC stainings performed on two different passages of two tumoroid models (i.e., after the acquisition of the tumor sample and before the drug screening was performed) (Figure EV3A) show a high concordance concerning the heterogeneity of marker protein expression when compared to the primary tumor and the tumoroid model at the time of drug screening (Fig. 2C). This indicates that there are no major changes concerning the composition of tumor cells acquired over the course of expanding the culture for drug screening." (lines 171 to 177)

For convenience, we also included the figure panel below (the afore-mentioned new Expanded View Fig. 3A):

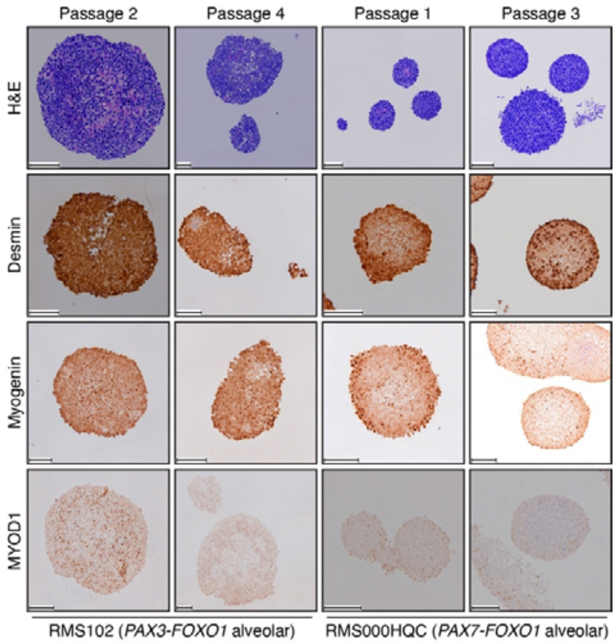

In summary, we provide evidence that our RMS tumoroid models indeed retain clonal heterogeneity as discussed by the referee.

Changes in the manuscript to account for these additional analyses, to conform with the publication standards of *EMBO Molecular Medicine*, and to perform minor corrections (e.g., author affiliations) were highlighted in the revised manuscript. Furthermore, with the addition of a new Expanded View Figure 3 (see above), previous Supplementary Figures were relabeled (previous Suppl. Fig. 3 to 5 are now Expanded View Figures 4 and 5 and Appendix Figure S1, respectively).

27th Jun 2022

Dear Dr. Drost,

Thank you for the submission of your manuscript to EMBO Molecular Medicine. We have now received the enclosed report from referee #2 who re-reviewed your manuscript. As you will see, this referee is now supportive of publication, and I am therefore pleased to inform you that we will be able to accept your manuscript once the following editorial points will be addressed:

1/ Main manuscript text:
- Please address the queries from our data editors in track changes mode in the main manuscript file labelled 'Data edited MS file'. Kindly use this file for any further modification, remove the highlighted text and only keep in track changes mode any new modification.
- Please replace "Summary" by "Abstract".
- We can accommodate a maximum of 5 keywords, please remove one of the six keywords currently listed.
- The material and methods section should be placed after the discussion and before the Acknowledgements. The grants and financial support should be part of the Acknowledgements.
- We updated our journal's competing interests policy in January 2022 and request authors to consider both actual and perceived competing interests. Please review the policy https://www.embopress.org/competing-interests and update your competing interests if necessary.
- In the Material and Methods, please include a statement that informed consent was obtained from all subjects and that the experiments conformed to the principles set out in the WMA Declaration of Helsinki and the Department of Health and Human Services Belmont Report.
- The Data Availability section should be placed at the end of the Material and Methods section. Please remove the sentence "the process of uploading ... before final publication" once all data are deposited. Please remove "Code for single-cell RNA sequencing analysis is available upon request".

2/ Figures:
- Table 1 should be an editable word file.
- Table EV1 might be best displayed as Dataset EV1 as there are many columns.
- Please make sure that the figures are referenced chronologically in the text (Fig. EV3A currently called out before Fig. EV1B).
- We would also encourage you to include the source data for figure panels that show essential data. Numerical data should be provided as individual .xls or .csv files (including a tab describing the data). For blots or microscopy, uncropped images should be submitted (using a zip archive if multiple images need to be supplied for one panel). Additional information on source data and instruction on how to label the files are available at
.

3/ Checklist:
Please correct "Code Availability section" by "Data Availability section".

4/ The Paper Explained:
Thank you for providing a TPE. Please include it in the main manuscript file, and structure it into three parts: Problem/Results/Impact (no references).
This may be edited to ensure that readers understand the significance and context of the research. Please refer to any of our published articles for an example.

5/ Thank you for providing a synopsis. Please also provide a synopsis image or visual abstract as a PNG/TIFF/JPEG file 550 px wide x 300-600 px high.

6/ As part of the EMBO Publications transparent editorial process initiative (see our Editorial at http://embomolmed.embopress.org/content/2/9/329), EMBO Molecular Medicine will publish online a Review Process File (RPF) to accompany accepted manuscripts.
This file will be published in conjunction with your paper and will include the anonymous referee reports, your point-by-point response and all pertinent correspondence relating to the manuscript. Let us know whether you agree with the publication of the RPF and as here, if you want to remove or not any figures from it prior to publication.
Please note that the Authors checklist will be published at the end of the RPF.

I look forward to receiving your revised manuscript.

Yours sincerely,

Lise Roth

Lise Roth, PhD
Senior Editor
EMBO Molecular Medicine

***** Reviewer's comments *****

Referee #2 (Comments on Novelty/Model System for Author):

The authors provide a new organoid/tumorsphere system that obviates the need for prior establishment of patient-derived xenografts in mice. This could accelerate pre-clinical testing.

Referee #2 (Remarks for Author):

Thank you for your rigorous response and congratulations on the nice body of work. I look forward to seeing how your system extends our knowledge of the biology of and therapies for RMS.

***
Rev_Com_number: RC-2022-01227
New_manu_number: EMM-2022-16001-V2
Corr_author: Drost
Title: Mesenchymal tumor organoid models recapitulate rhabdomyosarcoma subtypes

The authors performed the requested editorial changes.

12th Jul 2022

Dear Dr. Drost,

Thank you for submitting the revised files. I am pleased to inform you that your manuscript is accepted for publication and is now being sent to our publisher to be included in the next available issue of EMBO Molecular Medicine!

Please note that I slightly modified your "Paper Explained" as follows:
Problem: Rhabdomyosarcomas (RMS) are the most diagnosed soft tissue sarcomas in children and adolescents. Despite advances in treatment, prognosis for high-risk RMS patients remains poor, in part due to preclinical models that insufficiently recapitulate the disease.
Results: We describe the first collection of well-characterized RMS tumor organoid (tumoroid) preclinical models, which constitutes the first collection of tumoroids derived from purely malignant mesenchymal tumors (i.e., sarcomas) and only the second tumoroid model collection of pediatric cancers. Established models cover all major subtypes and clinical presentations of RMS, and faithfully recapitulate the molecular biology of the primary tumor sample they were derived from. Of note, RMS tumoroid models not only retain key driver mutations but also transcriptional and clonal heterogeneity in vitro. RMS tumoroid models can be rapidly established and expanded to perform drug screening as fast as 27 days after sample acquisition (median 81 days), indicating their potential for precision medicine approaches to inform treatment decisions. Lastly, RMS tumoroid models are amenable to CRISPR/Cas9-based editing to generate biologically relevant mutants like P53 deficient fusion-negative RMS (FN-RMS), recently shown to have worse prognosis than P53 proficient FN-RMS.
Impact: RMS tumoroid models will be valuable additions to the repertoire of preclinical models of RMS to ultimately improve treatment and prognosis of patients suffering from this highly aggressive disease.

Please let me know as soon as possible if you agree with it or if you would like to amend it.

We would like to remind you that as part of the EMBO Publications transparent editorial process initiative, EMBO Molecular Medicine will publish a Review Process File online to accompany accepted manuscripts. If you do NOT want the file to be published or would like to exclude figures, please immediately inform the editorial office via e-mail.

Congratulations on your interesting work!

With kind regards,

Lise Roth

Lise Roth, Ph.D
Senior Editor
EMBO Molecular Medicine

Follow us on Twitter @EmboMolMed

EMBO Press Author Checklist

Corresponding Author Name: Jarno Drost, PhD; Frank Holstege, Prof.	
Journal Submitted to: EMBO Molecular Medicine	
Manuscript Number: EMM-2022-16001	

USEFUL LINKS FOR COMPLETING THIS FORM
The EMBO Journal - Author Guidelines
EMBO Reports - Author Guidelines
Molecular Systems Biology - Author Guidelines
EMBO Molecular Medicine - Author Guidelines

Reporting Checklist for Life Science Articles (updated January 2022)
This checklist is adapted from Materials Design Analysis Reporting (MDAR) Checklist for Authors. MDAR establishes a minimum set of requirements in transparent reporting in the life sciences (see Statement of Task: 10.31222/osf.io/9sm4x). Please follow the journal's guidelines in preparing your manuscript.
Please note that a copy of this checklist will be published alongside your article.

Abridged guidelines for figures
1. Data
The data shown in figures should satisfy the following conditions:
- the data were obtained and processed according to the field's best practice and are presented to reflect the results of the experiments in an accurate and unbiased manner.
- ideally, figure panels should include only measurements that are directly comparable to each other and obtained with the same assay.
- plots include clearly labeled error bars for independent experiments and sample sizes. Unless justified, error bars should not be shown for technical replicates.
- if n<5, the individual data points from each experiment should be plotted. Any statistical test employed should be justified.
- Source Data should be included to report the data underlying figures according to the guidelines set out in the authorship guidelines on Data Presentation.

2. Captions
Each figure caption should contain the following information, for each panel where they are relevant:
- a specification of the experimental system investigated (eg cell line, species name).
- the assay(s) and method(s) used to carry out the reported observations and measurements.
- an explicit mention of the biological and chemical entity(ies) that are being measured.
- an explicit mention of the biological and chemical entity(ies) that are altered/varied/perturbed in a controlled manner.
- the exact sample size (n) for each experimental group/condition, given as a number, not a range;
- a description of the sample collection allowing the reader to understand whether the samples represent technical or biological replicates (including how many animals, litters, cultures, etc.).
- a statement of how many times the experiment shown was independently replicated in the laboratory.
- definitions of statistical methods and measures:
  - common tests, such as t-test (please specify whether paired vs. unpaired), simple χ2 tests, Wilcoxon and Mann-Whitney tests, can be unambiguously identified by name only, but more complex techniques should be described in the methods section;
  - are tests one-sided or two-sided?
  - are there adjustments for multiple comparisons?
  - exact statistical test results, e.g., P values = x but not P values < x;
  - definition of 'center values' as median or average;
  - definition of error bars as s.d. or s.e.m.

Please complete ALL of the questions below.
Select "Not Applicable" only when the requested information is not relevant for your study.

Materials

Newly Created Materials	Information included in the manuscript?	In which section is the information available? (Reagents and Tools Table, Materials and Methods, Figures, Data Availability Section)
New materials and reagents need to be available; do any restrictions apply?	Not Applicable	

Antibodies	Information included in the manuscript?	In which section is the information available? (Reagents and Tools Table, Materials and Methods, Figures, Data Availability Section)
For **antibodies** provide the following information:<br>- Commercial antibodies: RRID (if possible) or supplier name, catalogue number and or/clone number<br>- Non-commercial: RRID or citation	Yes	Materials & Methods section

DNA and RNA sequences	Information included in the manuscript?	In which section is the information available? (Reagents and Tools Table, Materials and Methods, Figures, Data Availability Section)
**Short novel DNA or RNA including primers, probes**: provide the sequences.	Yes	Materials & Methods section

Cell materials	Information included in the manuscript?	In which section is the information available? (Reagents and Tools Table, Materials and Methods, Figures, Data Availability Section)
**Cell lines:** Provide species information, strain. Provide accession number in repository **OR** supplier name, catalog number, clone number, and/**OR** RRID.	Yes	Materials & Methods section
**Primary cultures:** Provide species, strain, sex of origin, genetic modification status.	Yes	Throughout the manuscript (e.g., Fig. 1B, Table 1 etc.)
Report if the cell lines were recently **authenticated** (e.g., by STR profiling) and tested for mycoplasma contamination.	Yes	Materials & Methods section

Experimental animals	Information included in the manuscript?	In which section is the information available? (Reagents and Tools Table, Materials and Methods, Figures, Data Availability Section)
**Laboratory animals or Model organisms:** Provide species, strain, sex, age, genetic modification status. Provide accession number in repository **OR** supplier name, catalog number, clone number, **OR** RRID.	Not Applicable	
**Animal observed in or captured from the field:** Provide species, sex, and age where possible.	Not Applicable	
Please detail **housing and husbandry conditions**.	Not Applicable	

Plants and microbes	Information included in the manuscript?	In which section is the information available? (Reagents and Tools Table, Materials and Methods, Figures, Data Availability Section)
**Plants:** provide species and strain, ecotype and cultivar where relevant, unique accession number if available, and source (including location for collected wild specimens).	Not Applicable	
**Microbes:** provide species and strain, unique accession number if available, and source.	Not Applicable	

Human research participants	Information included in the manuscript?	In which section is the information available? (Reagents and Tools Table, Materials and Methods, Figures, Data Availability Section)
If collected and within the bounds of privacy constraints report on age, sex and gender or ethnicity for all study participants.	Yes	Materials & Methods section

Core facilities	Information included in the manuscript?	In which section is the information available? (Reagents and Tools Table, Materials and Methods, Figures, Data Availability Section)
If your work benefited from core facilities, was their service mentioned in the acknowledgments section?	Yes	Acknowledgements

Design

Study protocol	Information included in the manuscript?	In which section is the information available? (Reagents and Tools Table, Materials and Methods, Figures, Data Availability Section)
If study protocol has been **pre-registered, provide DOI in the manuscript**. For clinical trials, provide the trial registration number **OR** cite DOI.	Not Applicable	
Report the **clinical trial registration number** (at ClinicalTrials.gov or equivalent), where applicable.	Not Applicable	

Laboratory protocol	Information included in the manuscript?	In which section is the information available? (Reagents and Tools Table, Materials and Methods, Figures, Data Availability Section)
Provide DOI OR other citation details if **external detailed step-by-step protocols** are available.	Not Applicable	

Experimental study design and statistics	Information included in the manuscript?	In which section is the information available? (Reagents and Tools Table, Materials and Methods, Figures, Data Availability Section)
Include a statement about **sample size** estimate even if no statistical methods were used.	Not Applicable	
Were any steps taken to minimize the effects of subjective bias when allocating animals/samples to treatment (e.g. **randomization procedure**)? If yes, have they been described?	Not Applicable	
Include a statement about **blinding** even if no blinding was done.	Not Applicable	
Describe **inclusion/exclusion criteria** if samples or animals were excluded from the analysis. Were the criteria pre-established?<br><br>If sample or data points were omitted from analysis, report if this was due to attrition or intentional exclusion and provide justification.	Not Applicable	
For every figure, are **statistical tests** justified as appropriate? Do the data meet the assumptions of the tests (e.g., normal distribution)? Describe any methods used to assess it. Is there an estimate of variation within each group of data? Is the variance similar between the groups that are being statistically compared?	Yes	Materials & Methods section

Sample definition and in-laboratory replication	Information included in the manuscript?	In which section is the information available? (Reagents and Tools Table, Materials and Methods, Figures, Data Availability Section)
In the figure legends: state number of times the experiment was **replicated** in laboratory.	Yes	Respective figure legends
In the figure legends: define whether data describe **technical or biological replicates**.	Yes	Respective figure legends

Ethics

Ethics	Information included in the manuscript?	In which section is the information available? (Reagents and Tools Table, Materials and Methods, Figures, Data Availability Section)
Studies involving **human participants**: State details of **authority granting ethics approval** (IRB or equivalent committee(s), provide reference number for approval.	Yes	Materials & Methods section
Studies involving **human participants**: Include a statement confirming that **informed consent** was obtained from all subjects and that the experiments conformed to the principles set out in the WMA Declaration of Helsinki and the Department of Health and Human Services Belmont Report.	Yes	Materials & Methods section
Studies involving **human participants:** For publication of **patient photos**, include a statement confirming that consent to publish was obtained.	Not Applicable	
Studies involving experimental **animals**: State details of **authority granting ethics approva**l (IRB or equivalent committee(s), provide reference number for approval. Include a statement of compliance with ethical regulations.	Not Applicable	
Studies involving **specimen and field samples:** State if relevant **permits** obtained, provide details of authority approving study; if none were required, explain why.	Not Applicable	

Dual Use Research of Concern (DURC)	Information included in the manuscript?	In which section is the information available? (Reagents and Tools Table, Materials and Methods, Figures, Data Availability Section)
Could your study fall under dual use research restrictions? Please check biosecurity documents and list of **select agents and toxins** (CDC): https://www.selectagents.gov/sat/list.htm	Not Applicable	
If you used a select agent, is the security level of the lab appropriate and reported in the manuscript?	Not Applicable	
If a study is subject to dual use research of concern regulations, is the name of the **authority granting approval and reference number** for the regulatory approval provided in the manuscript?	Not Applicable	

Reporting

The MDAR framework recommends adoption of discipline-specific guidelines, established and endorsed through community initiatives. Journals have their own policy about requiring specific guidelines and recommendations to complement MDAR.

Adherence to community standards	Information included in the manuscript?	In which section is the information available? (Reagents and Tools Table, Materials and Methods, Figures, Data Availability Section)
State if relevant guidelines or checklists (e.g., **ICMJE, MIBBI, ARRIVE, PRISMA**) have been followed or provided.	Not Applicable	
For **tumor marker prognostic studies,** we recommend that you follow the **REMARK** reporting guidelines (see link list at top right). See author guidelines, under 'Reporting Guidelines'. Please confirm you have followed these guidelines.	Not Applicable	
For **phase II and III randomized controlled trials**, please refer to the **CONSORT** flow diagram (see link list at top right) and submit the CONSORT checklist (see link list at top right) with your submission. See author guidelines, under 'Reporting Guidelines'. Please confirm you have submitted this list.	Not Applicable	

Data Availability

Data availability	Information included in the manuscript?	In which section is the information available? (Reagents and Tools Table, Materials and Methods, Figures, Data Availability Section)
Have **primary datasets** been deposited according to the journal's guidelines (see 'Data Deposition' section) and the respective accession numbers provided in the Data Availability Section?	Yes	Data Availability section
Were **human clinical and genomic datasets** deposited in a public access-controlled repository in accordance to ethical obligations to the patients and to the applicable consent agreement?	Yes	Data Availability section
Are **computational models** that are central and integral to a study available without restrictions in a machine-readable form? Were the relevant accession numbers or links provided?	Yes	Data Availability section
If publicly available data were reused, provide the respective **data citations in the reference list**.	Not Applicable	
